# Supplementary material for: Comprehensive Lichenometabolomic Exploration of Ramalina conduplicans Vain Using UPLC-Q-ToF-MS/MS: An Identification of Free Radical Scavenging and Anti-Hyperglycemic Constituents
Source: Molecules. 2022 Oct 9;27(19):6720. doi: 10.3390/molecules27196720 (PMC9570585; doi:10.3390/molecules27196720)
Supplement: Supplementary file 1 [file molecules-27-06720-s001.zip › molecules-1905222-supplementary.pdf]

Comprehensive Lichenometabolomic Exploration of *Ramalina conduplicans* Vain using UPLC-Q-ToF-MS/MS: An Identification of Free Radical Scavenging and Anti-Hyperglycemic Constituents

Tatapudi Kiran Kumar [1,2,†], Bandi Siva [1,†], Ajay Anand [1], Komati Anusha [1], Satish Mohabe [3,4], Araveeti Madhusudana Reddy [3], Françoise Le Devehat [5], Ashok Kumar Tiwari [1,2], Joël Boustie [5,*] and Katragadda Suresh Babu [1,2,*]

[1] *Centre for Natural Products & Traditional Knowledge, CSIR-Indian Institute of Chemical Technology, Uppal Road, Tarnaka, Hyderabad 500007, India*

[2] *Academy of Scientific and Innovative Research (AcSIR), Ghaziabad 201002, India*

[3] *Department of Botany, Yogi Vemana University, Vemanapuram, Kadapa 516003, India*

[4] *Faculty of Sciences & IT, Madhyanchal Professional University, Ratibad, Bhopal 462044, India*

[5] *Institut des Sciences Chimiques de Rennes, Université Rennes, CNRS, ISCR-UMR6226, 35000 Rennes, France*

* To whom correspondence should be addressed. Tel: +91-40-27191881; Fax: +91-40-27160512;

E-Mail: suresh@iict.res.in (Dr. K. S. Babu), joel.boustie@univ-rennes1.fr (J.B.)

† Equal contribution.

Materials and Methods (with additional details)

General

The NMR spectra were recorded on a Bruker Evans III HD 400 MHz spectrometer at 400 MHz for ^{1}H and 100 MHz for ^{13}C respectively, using 1 pulse programme at 250 C. Samples were dissolved in Acetone-d6 and Trimethylsilane (TMS) was used as internal standard. ^{1}H NMR experiment of each sample recorded with 32 scans and ^{13}C NMR experiment with 68 scans. The chemical shifts are expressed as δ values in parts per million (ppm) and the coupling constants (J) are given in hertz (Hz). The 2D experiments (1H–1H COSY, HSQC and NOESY) were performed using standard Bruker microprograms. Mass data were acquired on Xevo™ G2 XS-ESI-QTof mass spectrometer (Waters Corp., Manchester, UK). For thin layer chromatography (TLC) analysis, precoated Merck plates (silica gel 60 F_{254}) were utilized. Silica gel (100-200 mesh) (Qing-dao Marine Chemical, Inc., Qingdao, China) was chosen for column chromatographic separation. Semi-preparative chromatography was performed on Gilson HPLC (Middleton, WI, USA) instrument equipped with 321 binary pump, GX-281 liquid handler and UV-155 detector with X Select HSS T3 (250 mm × 100 mm, 5 µm) (Waters Corp., Ireland) as stationary phase using Trilution LC v2.1 platform. Formic acid (Optima™Mass spec grade) from Thermo Fisher Scientific (Geel, Belgium), HPLC grade acetonitrile and (LiChrosolv) from Merck (Darmstadt, Germany) and Ultra-pure water (Millipore system, Massachusetts, USA) were used.

Instrumental UPLC conditions

Instrumental conditions were setup as per our recent report (Reddy et al., 2019) with slight modifications. Chromatographic separation was performed on Acquity H Class UPLC system (Waters, Milford, MA, USA) with a conditioned auto sampler, using an ACQUITY UPLC CSH Phenyl-Hexyl column (100 mm × 2.1 mm id., 1.7 µm particle size) (Waters, Milford, MA, USA). Column temperature was maintained at 40°C. High-resolution masses of secondary metabolites were measured after UPLC separation. Mobile phase consisting of water with 0.1% formic acid in water (solvent A) and acetonitrile with 0.1% formic acid (solvent B) was pumped at flow rate of 0.4 mL/min. Gradient elution program was as follows: 0 min, 5% B; 3.00 min, 20% B; 5.00min, 35% B; 7.50 min, 50% B; 10.00 min, 70% B; 12.50 min, 95% B; 17.00 min 95% B; 21.00 min 5% B. Equilibration time was 4.0 min and injection volume was 2 µL. LC-QTof-MS^E mode was applied to analyze the samples in both TIC as well as MS/MS mode where collision energy was ramped at 15 eV – 45 eV. Eluted compounds were detected from *m/z* 50 to 1200 using Xevo G2-XS Q-Tof mass spectrometer (Waters, Manchester, UK), which was connected to Electro spray ionization (ESI) interface with negative ion mode using the following instrument settings, capillary voltage, 2.0 KV; sample cone, 40 V; source temperature, 120 °C; desolvation temperature 350 °C; cone gas flow rate 50 L/h; desolvation gas (N_2) flow rate 850 L/h, argon as CID gas for MS/MS experiments. All analyses were performed using the lock spray, which ensured accuracy and reproducibility. Leucine - Enkephalin (5 ng/mL) was used as lock mass, generating a reference ion in negative mode at *m/z* 554.2615 introduced by a lock spray at 10 µL/min for accurate mass acquisition. Data acquisition was achieved using MassLynx v4.1. Acquiring data in this manner provided information of intact precursor ions as well as fragment ions.

Lichen Sample collection and identification

The lichen, *Ramalina conduplicans* was collected from tree bark in Bichpuri Range, Bijrani Zone of Corbett National Park, alt. N 29°26′40″ E79°04′06 (1283 m.) in the month of May 2019.The morphological features of lichen thallus and ascomata were observed under Magnüs MS 24/13, spot tests for color reaction were carried out by 10% aqueous solution of potassium hydroxide (K), Steiner's stable *p*-phenylene diamine solution (PD) and calcium hypochlorite solution (C). For anatomical investigation of fruiting bodies light microscope of ZEISS Axiostar was used. The lichen substances were identified with thin Layer Chromatography in solvent system 'A' following White and James, 1985. The standard literatures [1] were referred for identification of lichen samples. Voucher specimen (Satish Mohabe & A.Madhusudhana Reddy 7658YVUH) of species was deposited at the Herbarium, Department of Botany, Yogi Vemana University, Kadapa, Andhra Pradesh. Corresponding data are shown in supporting information.

Extraction and isolation

The sorted-out lichen *Ramalina conduplicans* (300 g) was shade dried, powdered and extracted with acetone (6L) at room temperature for 48 h. Resulting acetone extract was evaporated to dryness under reduced pressure affording syrupy residue (20 g). This crude extract was subjected to gradient column chromatography (SiO_2, 60–120 mesh), eluting with hexane/EtOAc mixture of increasing polarity with 10% intervals yielded 8 fractions. These 8 fractions were reconstituted in acetonitrile and subjected to UPLC Q-ToF MS^E. Based on TIC profile, we selected fractions 3–6 for purification (mass profile shown in supporting information, discussion in results section). All these fractions were subjected to semi-preparative HPLC (X Select HSST3 OBD Prep Column 5 µm, 10 mm × 250 mm, 0.1% formic acid with water (solvent A) and acetonitrile (solvent B) as mobile phase at flow rate 4 mL/min, detected at 254 nm. Since the mobile phase composition aligns with diligent composition, the interference of the solvent UV spectra with compound UV spectra is minimal. We also used control for avoiding the solvent matrix in the analysis. The method uses MS as additional tool to confirm the eluted compounds from UV. Semi-preparative HPLC were conducted by gradient elution programs to obtain compounds as follows: Fraction 3 (quantity 70 mg , loop volume 250 µL was eluted by 0 min, 5%B; 5 min, 5%B; 10.00 min, 35% B; 16.00 min, 60% B; 25 min, 95% B; 30 min, 95% B; 5% B; 30.50 min, 5% B; 35.00 min. to yield **3** (homosekikaic acid, 7 mg), **7** (divaricatic acid, 4 mg), **11** (divaricatinic acid, 3 mg), **12** (olivetolic acid, 5 mg), and **14** (atranol,2 mg).Fraction 4 (quantity 50 mg , loop volume 250 µL was eluted by 0 min, 30%B; 5 min, 30%B; 10.00 min, 50% B; 23.00 min, 95%B; 27.00 min, 95% B; 27.50 min, 30% B; 30% B; 30.00 min. at flow rate 4 mL/min, detected at 254 nm to yield **10** (methyldivaricatinate, 3 mg), **8** (decarboxydivaricatic acid, 5 mg), **9** (decarboxystenosporic acid, 2 mg), and **4** (hyperhomosekikaic acid, 1 mg). Fraction 5 (quantity 25 mg, loop volume 250 µL was eluted by 0 min, 5% B; 8.50 min, 30% B; 15.00 min, 50% B; 22.00 min, 95% B; 28.00 min, 95% B; 29.0 min, 5% B; 5% B; 35.00 min at flow rate 4 mL/min, detected at 254 nm) to yield **13** (divarinolmonomethylether, 3 mg) and compound **5** (2 mg). Fraction 6 (quantity 40 mg, loop volume 250 µL was eluted by 0 min, 10% B; 8.50 min, 40% B; 18.00 min, 55% B; 25.00 min, 75% B; 32.00 min, 95% B; 36.0 min, 95% B; 10% B; 37.00 min, 10% B; 42.00 min at flow rate 4 mL/min, detected at 254 nm) to yield **1** (sekikaic acid, 5 mg), **2** (4-*O*-methylnorhomosekikaic acid, 7 mg) and 2,4-di-*O*-methyldivaric acid **6** (2 mg).

In-vitro Antihyperglycemic and antioxidant assay

DPPH radical scavenging activity

DPPH radical scavenging assay was carried out as previously reported [2]. Scavenging of 2,2-diphenyl-1-picryhydrazyl (DPPH) radicals by the Acetone extract (AE) (50 μg of 2 mg/mL solution dissolved in DMSO) and compounds (**1-14**) (50 μg of 2 mg/mL solution dissolved in DMSO) was measured in 100 mM Tris-HCl buffer (pH 7.4) by recording absorbance at 517 nm spectrophotometrically. Ascorbic acid (50 μg of 2 mg/mL solution dissolved in DMSO) served as standard. Results were expressed as % scavenging and calculated by using formula: A_c-A_t)/100*A_c, where A_c was the absorbance of control and A_t, the absorbance of test sample. Different concentrations of compounds were evaluated to obtain 50% scavenging activity (SC_{50}). The SC_{50} was calculated based on equation obtained from regression analysis.

ABTS radical scavenging activity

Scavenging of 2,2′-azino-bis(3-ethylbenzothiazoline-6-sulphonic acid) radical cation ($ABTS^{+\cdot}$) was performed as per earlier method [3]. Acetone extract (AE) (20 μg of 2 mg/mL solution dissolved in DMSO) and compounds (**1-14**) (20 μg of 2 mg/mL solution dissolved in DMSO) were incubated with $ABTS^{+\cdot}$ solution in 6.8 mM phosphate buffer (pH 8.0) as described earlier. Decoloration of $ABTS^{+\cdot}$ solution was determined by measuring absorbance at 734 nm spectrophotometrically. Ascorbic acid (20 μg of 2 mg/mL solution dissolved in DMSO) served as standard. The activity was expressed as % scavenging and calculated as follows: (A_c-A_t)/100*A_c, where A_c was the absorbance of control and A_t, the absorbance of test sample. SC_{50} of compounds was calculated as per above formula.

Free radical induced DNA damage

Protective effect of acetone extract (AE) and Compounds (**1–14**) on oxidative DNA damage was evaluated as per the previous method [4]. 2 μL calf-thymus DNA mixed with 5 μL of 39 mM Tris buffer (pH 7.4) and 5 μL (10 μg) acetone extract and compounds (**1–14**) (10 μg of 2 mg/mL solution dissolved in DMSO) mixture was incubated at room temperature for 20 min. Reaction was initiated by adding 5 μL $FeCl_3$ (500 μM) and 10 μL H_2O_2 (0.8 M) and incubated for 10 min at 37 °C. Reaction was stopped by adding 3 μL DNA loading dye. Finally, mixture was subjected to 0.8% agarose gel electrophoresis in TAE (40 mM Tris, 20 mM acetic acid and 0.5 M EDTA) buffer (pH 7.2). 3 μL of Ethidium bromide was added to agarose solution to stain DNA bands. Image was viewed under trans illuminating UV light and photographed (Bio-Rad ChemiDocTM XRS with Image LabTM Software). Band intensity of DNA was measured by using ImageJ software.

Intestinal α-glucosidase inhibition

Intestinal α-glucosidase enzyme inhibition assay was performed as per previous method [2]. 20 μL (40 μg) of acetone extract and compounds (**1–14**) (40 μg of 2 mg/mL solution dissolved in DMSO) were incubated with 50 μL of rat intestinal α-glucosidase enzyme (89.93 mM, prepared in 0.9% NaCl) in 100 mM Phosphate buffer (pH 6.8) for 10 min. After incubation period, 50 μL of substrate (4-nitroplenyl α-D-glucopyranoside) solution was added. Release of *p*-nitrophenol from substrate was measured by recording absorbance at 405 nm spectrophotometrically. Acarbose (40 μg of 2 mg/mL solution dissolved in DMSO) was taken as standard. The activity was expressed and calculated as follows: (A_c-A_t)/100*A_c, where A_c was the absorbance of control and A_t, the absorbance of test sample.

Statistical analysis

Comparisons within the groups were done applying one way ANOVA followed by post-test; Tukey's Multiple comparison test Statistical significance was set at $p < 0.05$. Data analysis was performed by using GraphPad PRISM Version 5.01 (GraphPad Software Inc. California, USA).

Table S1: LC-QToF-MS^E data of compounds from acetone extract of *R. conduplicans*

Ret time	Mol. Wt. with Adduct *m/z* Adduct	Formula	Error (Δppm)	Fragment ions	Compound name
7.05	387.0358 [M − H]$^-$	$C_{18}H_{11}O_{10}$	1.6	343.0457 ($C_{17}H_{11}O_8$), 325.0363 ($C_{17}H_9O_7$), 313.0357 ($C_{16}H_9O_7$), 299.0560 ($C_{16}H_{11}O_6$), 269.0454 ($C_{15}H_9O_5$), 243.0301 ($C_{13}H_7O_5$), 241.0507 ($C_{14}H_9O_4$), 227.0350 ($C_{13}H_7O_4$), 185.0608 ($C_{12}H_9O_2$), 121.0292 ($C_7H_5O_2$).	Salazinic acid$^{\&}$
7.50	195.0657 [M − H]$^-$	$C_{10}H_{11}O_4$	0.0	151.0756 ($C_9H_{11}O_2$).	Divaric acid$^{\&}$
8.00	151.0396	$C_8H_7O_3$	0.7	123.0445 ($C_7H_7O_2$), 105.0339 (C_7H_5O) 81.0331 (C_5H_5O).	Atranol (**14**)*
8.53	309.1704 [M − H]$^-$	$C_{17}H_{25}O_5$	0.6	265.1805 ($C_{16}H_{25}O_3$), 209.1540($C_{13}H_{21}O_2$), 152.0835 ($C_9H_{12}O_2$).	ND
9.13	223.0970 [M − H]$^-$	$C_{12}H_{15}O_4$	0.0	179.1070 ($C_{11}H_{15}O_2$), 137.0967 ($C_9H_{13}O$).	Olivetolic acid (**12**)*
9.22	223.0969 [M − H]$^-$	$C_{12}H_{15}O_4$	−0.4	179.1071 ($C_{11}H_{15}O_2$), 165.0915 ($C_9H_{13}O$), 151.0764 ($C_9H_{11}O_2$), 137.0968 ($C_9H_{13}O$).	Methyldivaric atinate (**10**)
9.26	209.0813 [M − H]$^-$	$C_{11}H_{13}O_4$	−0.5	165.0913($C_{10}H_{13}O_2$), 150.0679 ($C_9H_{10}O_2$), 122.0363 ($C_7H_6O_2$).	Divaricatinic acid (**11**)

9.36	165.0914 [M − H]$^-$	$C_{10}H_{13}O_2$	−1.2	150.0678 ($C_9H_{10}O_2$).	Divarinolmonomethylether (**13**)*
10.29	295.2263 [M − H]$^-$	$C_{18}H_{31}O_3$	−3.4	277.2149($C_{18}H_{29}O_2$), 233.1534($C_{15}H_{21}O_2$), 195.1378($C_{12}H_{19}O_2$), 171.1055($C_9H_{15}O_3$), 167.0712($C_9H_{11}O_3$), 138.0320 ($C_7H_6O_3$).	ND
11.07	343.1554 [M − H]$^-$	$C_{20}H_{23}O_5$	2.6	299.1650($C_{19}H_{23}O_3$), 283.1335($C_{18}H_{19}O_3$), 177.0549($C_{10}H_9O_3$), 165.0915($C_{10}H_{13}O_2$), 151.0761($C_9H_{11}O_2$).	Decarboxy divaricatic acid (**8**)*
11.10	373.0934 [M − H]$^-$	$C_{19}H_{17}O_8$	2.9	195.1020($C_{11}H_{15}O_3$), 177.0163($C_9H_5O_4$), 163.0386($C_9H_7O_3$), 133.0263($C_8H_5O_2$), 119.0502 (C_8H_7O).	Atranorin[&]
11.25	343.0824 [M − H]$^-$	$C_{18}H_{15}O_7$	1.7	328.0587 ($C_{17}H_{12}O_7$), 259.0608($C_{14}H_{11}O_5$), 231.0657 ($C_{13}H_{11}O_4$)	Usnic acid[&]
11.81	371.1965 [M − H]$^-$	$C_{21}H_{27}O_3$	1.5	205.0864($C_{12}H_{13}O_3$), 179.1073 ($C_{11}H_{15}O_2$), 137.0966 ($C_9H_{13}O$).	Decarboxystenosporic acid (**9**)*
11.88	401.1983 [M − H]$^-$	$C_{23}H_{29}O_6$	2.0	209.1175($C_{12}H_{17}O_3$), 194.0952($C_{11}H_{14}O_3$), 166.0912 ($C_{10}H_{13}O_2$).	Compound -5[#]
11.95	223.0966 [M − H]$^-$	$C_{12}H_{15}O_4$	−1.8	209.0809 ($C_{11}H_{13}O_4$), 165.0915 ($C_{10}H_{13}O_2$), 150.0677($C_9H_{11}O_2$), 122.0365($C_7H_6O_2$).	2,4-di-*O*-methyldivaric acid (**6**)*
12.43	387.1443 [M − H]$^-$	$C_{21}H_{23}O_7$	−0.3	195.0656($C_{10}H_{11}O_4$), 177.0550 ($C_{10}H_9O_3$), 151.0756($C_9H_{11}O_2$),	Divaricatic acid (**7**) *

				133.0649(C_9H_9O).	
12.51	431.1686 [M − H]$^-$	$C_{23}H_{27}O_8$	−2.3	253.1070($C_{13}H_{17}O_5$), 195.0647 ($C_{10}H_{11}O_4$), 177.0528($C_{10}H_9O_3$), 151.0754 ($C_9H_{11}O_2$).	4’-*O*-Methyl norhomosekikaic acid (**2**)*
12.71	417.1547 [M − H]$^-$	$C_{22}H_{25}O_8$	−0.5	225.0761($C_{11}H_{13}O_5$), 209.0813($C_{11}H_{13}O_4$), 181.0861($C_{10}H_{13}O_3$), 166.0628 ($C_9H_{10}O_3$), 165.0913($C_{10}H_{13}O_2$), 150.0678($C_9H_{10}O_2$), 137.0235($C_7H_5O_3$), 122.0365 ($C_7H_6O_2$).	Sekikaic acid (**1**)*
13.17	415.1759 [M − H]$^-$	$C_{23}H_{27}O_7$	0.5	223.0971($C_{12}H_{15}O_4$), 205.0865 ($C_{12}H_{13}O_3$), 179.1071($C_{11}H_{15}O_2$), 161.0965 ($C_{11}H_{13}O$)	ND
13.42	445.1860 [M − H]$^-$	$C_{24}H_{29}O_8$	−0.4	253.1075($C_{13}H_{17}O_5$), 209.0813 ($C_{11}H_{13}O_4$), 194.0941($C_{11}H_{14}O_3$), 165.0913($C_{10}H_{13}O_2$), 150.0679 ($C_9H_{10}O_2$), 137.0237($C_7H_5O_3$), 122.0367($C_7H_6O_2$).	Homosekikaic acid (**3**)*
14.10	473.2178 [M − H]$^-$	$C_{26}H_{33}O_8$	1.5	253.1075($C_{13}H_{17}O_5$), 237.1127 ($C_{13}H_{17}O_4$), 209.0816 ($C_{12}H_{17}O_3$)	Hyper homosekikaic acid (**4**)*

ND = Not determined, * = Isolated compounds # = tentatively assigned new compound, & = identified in the extraction concluded through ms/ms and compare with the in-house sample.

Physicochemical data of isolated compounds:

4.4.1. Sekikaic acid (**1**) : White amorphous powder; IR (KBr) ν_{max} : 3538, 3489, 1710, 1426, 1360, 1221 cm^{-1}; ^{1}H NMR (400 MHz, Acetone-d_6): δ 6.70 (1H, s), 6.47 (1H, d, *J* = 2.69 Hz), 6.41 (1H, d, *J* = 2.69 Hz), 3.94 (3H, s), 3.87 (3H, s), 3.06 – 2.98 (4H, m), 1.80 – 1.64 (4H, m), 1.00 (3H, t, *J* = 7.33, 14.67 Hz), 0.93 (3H, t, *J* = 7.21, 14.67 Hz); ^{13}C NMR (100 MHz, Acetone-d_6): δ 174.60, 170.24, 166.79, 166.43, 157.78, 157.44, 150.11, 147.87, 126.57, 112.45, 108.04, 107.43, 106.31, 100.71, 57.57, 56.85, 40.28, 40.26, 27.06, 26.71, 15.58, 15.53 ppm. HRESIMS (*m/z*): 417.1553 $[M-H]^-$ (calcd for $C_{22}H_{25}O_8$, 417.1549).

4.4.2. 4′-*O*-methylnorhomosekikaic acid (**2**): White amorphous powder; IR (KBr) ν_{max} : 3626, 3598, 3010, 2253, 1713,1435, 1370, 1224,1040, 918 cm^{-1}; ^{1}H NMR (400 MHz, Acetone-d_6): δ 6.71(1H, s), 6.41 (1H, d, *J* = 2.44 Hz), 6.31 (1H, *J* = 2.44 Hz), 3.94 (3H, s), 3.08 – 3.04 (2H, m), 2.99 – 2.95 (2H, m), 1.79 – 1.72 (2H, m), 1.70 – 1.63 (2H, m), 1.42 – 1.37 (4H, m), 1.30 – 1.25 (2H, m), 0.95 – 0.89 (6H,m); ^{13}C NMR (100MHz, Acetone-d_6): δ 174.99, 170.37, 166.91, 164.61, 158.14, 157.52, 150.67, 148.11, 126.63, 113.07, 107.99, 107.41, 105.58, 102.75, 57.57, 40.32, 38.32, 33.81, 33.73, 26.68, 24.13, 15.60, 15.33 ppm. HRESIMS (*m/z*): 431.1706 $[M-H]^-$ (calcd for $C_{23}H_{27}O_8$, 431.1706).

2.4.3. Homosekikaic acid (**3**): White amorphous powder; IR (KBr) ν_{max}: 3781, 3634, 3595, 3008, 2946, 2295, 2253, 1714, 1435, 1371,1223, 1040, 919, 748 cm^{-1}; ^{1}H NMR (400 MHz, Acetone-d_6): δ 6.69 (1H, s), 6.46 (1H, d, *J* = 2.59 Hz), 6.40 (1H, d, *J* = 2.59 Hz), 3.93 (3H, s), 3.86 (3H, s), 3.06 – 3.03 (2H, m), 3.01–2.99 (2H, m), 1.78–1.72 (2H, m), 1.71– 1.63 (2H, m), 1.41– 1.36 (4H, m), 0.94– 0.89 (6H, m); ^{13}C NMR (100 MHz, Acetone-d_6): δ 174.65, 170.23, 166.74, 166.40, 158.06, 157.41, 150.08, 148.15, 126.56, 112.42, 107.93, 107.48, 106.36, 100.71, 57.55, 56.84, 40.23, 38.28, 33.79, 33.70, 26.71, 24.12, 15.57, 15.33 ppm. HRESIMS (*m/z*): 445.1859 $[M-H]^-$ (calcd for $C_{24}H_{29}O_8$, 445.1862).

2.4.4. Hyperhomosekikaic acid (**4**): Pale yellow amorphous powder, ^{1}H NMR (400 MHz, Acetone-d_6): δ 6.72 (1H, d, *J* = 1.8 Hz), 6.47 (1H, d, *J* = 2.5 Hz), 6.41(1H, d, *J* = 2.5 Hz), 3.87 (3H, s), 3.83 (3H, s), 3.01 – 2.96 (2H, m); 2.64 – 2.59 (2H, m); 1.76 – 1.62 (6H, m), 0.95 (3H, t, *J* = 7.5), 0.95 (3H, t, *J* = 7.3); HRESIMS (*m/z*): 473.2176 $[M-H]^-$ (calcd for $C_{26}H_{33}O_8$, 473.2175).

2.4.5. Compound **5**: White gummy liquid, ^{1}H NMR (400 MHz, Acetone-d_6) and ^{13}C NMR (100 MHz, Acetone-d_6): see in table-1. HRESIMS (*m/z*): 401.1961 $[M-H]^-$ (calcd for $C_{23}H_{29}O_6$, 401.1964).

2.4.6. 2,4-di-*O*-methyldivaric acid (**6**): yellow gum; IR (KBr) ν_{max}: 3537, 3003, 1711,1426,1360, 1221, 1093 cm^{-1}; ^{1}H NMR (400 MHz, Acetone-d_6): δ 6.46 (1H, d, *J* = 2.56 Hz) , 6.40 (1H, d, *J* = 2.56 Hz), 3.93 (3H, s) , 3.86 (3H, s) , 3.01 – 2.97 (2H, m), 1.77 – 1.72 (2H, m) , 0.91 (3H, t , *J* = 7.33 Hz); ^{13}C NMR (100 MHz, Acetone-d_6): δ 150.08, 112.49, 100.76, 57.85, 56.88, 40.29, 26.74, 15.30 ppm. HRESIMS *(m/z)*: 223.0975 $[M-H]^-$ (calcd for $C_{12}H_{15}O_4$, 223.0970).

2.4.7. Divaricatic acid (**7**): White amorphous powder; IR (KBr) ν_{max}: 3673, 3638, 3597, 3005, 2943, 2293, 2253, 1714, 1436, 1370, 1223, 1040, 918, 748 cm^{-1}; ^{1}H NMR (400 MHz, Acetone-d_6): δ 6.78 (1H, s), 6.75 (1H, s), 6.46 (1H, s), 6.41 (1H, s), 3.86 (3H, s), 3.00 (2H, t, *J* = 7.78, 15.41Hz), 2.95 (2H, t, *J* = 7.78, 15.56 Hz), 1.72 – 1.64 (4H, m), 0.98 – 0.93 (6H, m); ^{13}C NMR (100 MHz, Acetone-d_6): δ 174.08, 170.74, 166.93, 166.50, 165.76, 155.90, 149.96, 149.46, 117.49, 112.46, 112.40, 110.19, 106.55, 100.80, 56.89, 40.27, 39.65, 26.91, 26.68, 15.48, 15.36 ppm. HRESIMS *(m/z)*: 387.1451 $[M-H]^-$ (calcd for $C_{21}H_{23}O_7$, 387.1444).

2.4.8. Decarboxydivaricatic acid (**8**): White amorphous powder; ^{1}H NMR (400 MHz, Acetone-d_6): δ 11.24 (1H, s), 6.78 (1H, s), 6.75 (1H, s), 6.46 (1H, s), 6.41(1H, s), 3.86 (3H, s), 3.00 (2H, t, *J* = 7.78, 15.41 Hz), 2.56 (2H, t, *J* = 7.78, 15.56 Hz), 1.72 – 1.64 (4H, m), 0.98 – 0.93 (6H, m); ^{13}C NMR (100 MHz, Acetone-d_6): δ 171.53, 167.14, 166.40, 159.99, 152.91, 149.55, 147.10, 115.05, 114.60, 112.49, 108.31, 106.56,

100.79, 56.87, 40.41, 39.38, 27.01, 25.97, 15.49, 14.99 ppm. HRESIMS *(m/z)*: 343.1554 $[M - H]^-$ (calcd for $C_{20}H_{23}O_5$, 343.1545).

2.4.9. Decarboxystenosporic acid (**9**): White amorphous powder; ^1H NMR (400 MHz, Acetone-d_6): δ 11.24 (1H, s) , 6.67 – 6.65 (1H, m), 6.63 – 6.61 (1H, m), 6.60 – 6.58 (1H, m), 6.46 (1H, d *J* = 2.56 Hz), 6.40 (1H, d, *J* = 2.69 Hz), 3.87 (3H, s), 2.99 – 2.95 (2H, m) , 2.59 (2H, t, *J* = 7.58, 15.40 Hz), 1.75 – 1.68 (2H, m), 1.66 – 1.59 (2H, m), 1.37 – 1.32 (4H, m), 0.96 (6H, t, *J* = 7.33, 14.67 Hz); ^{13}C NMR (100 MHz, Acetone-d_6): δ 171.51, 167.11, 166.38, 159.99, 152.91, 149.54, 147.34, 114.98, 114.54, 112.48, 108.25, 106.57, 100.78, 56.87, 40.40, 37.28, 33.13, 32.60, 27.01, 24.12, 15.49, 15.27 ppm. HRESIMS (*m/z):* 371.1862 $[M - H]^-$ (calcd for $C_{22}H_{27}O_5$, 371.1858).

2.4.10. Methyldivaricatinate (**10**): White amorphous powder; ^1H NMR (400 MHz, Acetone-d_6): δ 11.53 (1H, s) , 6.37 (1H, d, *J* = 2.69 Hz) , 6.32 (1H, d, *J* = 2.69 Hz), 3.95 (3H, s) , 3.82 (3H, s) , 2.87– 2.83 (2H, m), 1.61 – 1.51 (2H, m) , 0.95 (3H, t , *J* = 7.45, 14.79 Hz); ^{13}C: (100 MHz, Acetone-d_6): δ 173 .57, 167.04, 165.95, 149.39, 112.13, 106.71, 100.69, 56.74, 53.39, 40.26, 26.72, 15. 45 ppm. HRESIMS *(m/z)*: 223.0964 $[M - H]^-$ (calcd for $C_{12}H_{15}O_4$, 223.0970).

2.4.11. Divaricatinic acid (**11**): White amorphous powder; ^1H NMR (400 MHz, Acetone-d_6): δ 6.35 (1H, d, *J* = 2.56 Hz), 6.32 (1H, d, *J* = 2.56 Hz), 3.82 (3H, s), 2.95 – 2.91 (2H, m), 1.65 – 1.56 (2H, m), 0.93 (3H, t, *J* = 7.33, 14.67 Hz); ^{13}C NMR (100 MHz, Acetone-d_6): δ 174.89, 167.90, 165.99, 150.07, 111.97, 106.13, 100.54, 56.70, 40.13, 26.81, 15.44 ppm. HRESIMS *(m/z)*: 209.0805 $[M - H]^-$ (calcd for $C_{11}H_{13}O_4$, 209.0814).

2.4.12. Olivetolic acid (**12**): White amorphous powder; ^1H NMR (400 MHz, Acetone-d_6): δ 6.28(1H, d, *J* = 2.4 Hz), 6.21 (1H, d, *J* = 2.4 Hz), 2.93 – 2.89 (2H, m) , 1.61 – 1.54 (2H, m), 1.34 – 1.32 (4H, m), 0.88 (3H, t, *J* = 6.8, 14.0 Hz); ^{13}C NMR (100 MHz, Acetone-d_6): δ 175.00, 167.88, 164.28, 150.79, 112.51, 105.36, 102.56, 38.12, 33.70, 33.45, 24.11, 15.30 ppm. HRESIMS (*m/z*): 223.0961 $[M - H]^-$ (calcd for $C_{12}H_{15}O_4$, 223.0970).

2.4.13. Divarinolmonomethylether (**13**): White amorphous powder; ^1H NMR (400 MHz, Acetone-d_6): δ 6.29 (1H, d, *J* = 2.5 Hz), 6.26 (1H, d, *J* = 2.45 Hz), 6.23 (1H, d, *J* = 2.4 Hz), 3.72 (3H, s), 2.48 – 2.44 (2H, m) , 1.62 – 1.56 (2H, m), 0.93(3H, t, *J* = 6.7, 14.5 Hz); ^{13}C NMR (100 MHz, Acetone-d_6): δ 162.81, 160.24, 146.60, 109.73, 107.18, 100.49, 56.24, 39.76, 26.07, 15.04 ppm. HRESIMS (*m/z):* 165.0916 $[M - H]^-$ (calcd for $C_{10}H_{13}O_2$, 165.0916).

2.5.14. Atranol (**14**): White amorphous powder; ^1H NMR (400 MHz, Acetone-d_6): δ ppm 10.28 (s, 1H), 6.24 (s, 2H), 2.03 (s, 3H); ^{13}C NMR (100 MHz, Acetone-d_6): 195.18, 164.03 (2C), 152.56, 110.27, 109.42, 109.42, 23.38 ppm. HRESIMS (*m/z)*: 151.0399 $[M - H]^-$ (calcd for $C_8H_7O_3$, 151.0395).

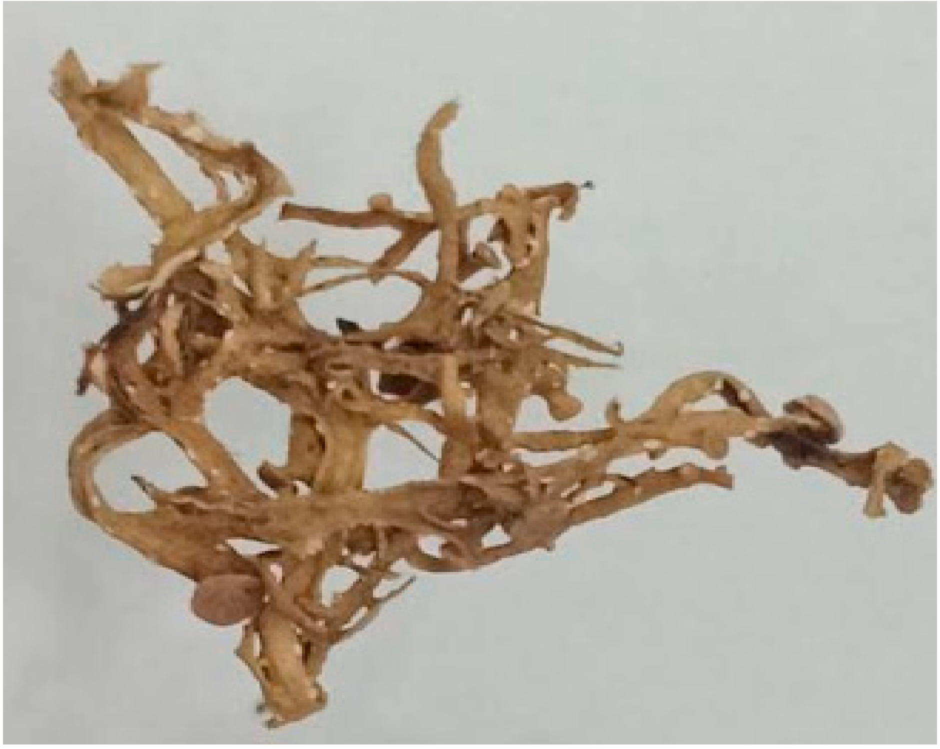

Figure S1: The authentic *Ramalina conduplicans.*

1 2 3 4

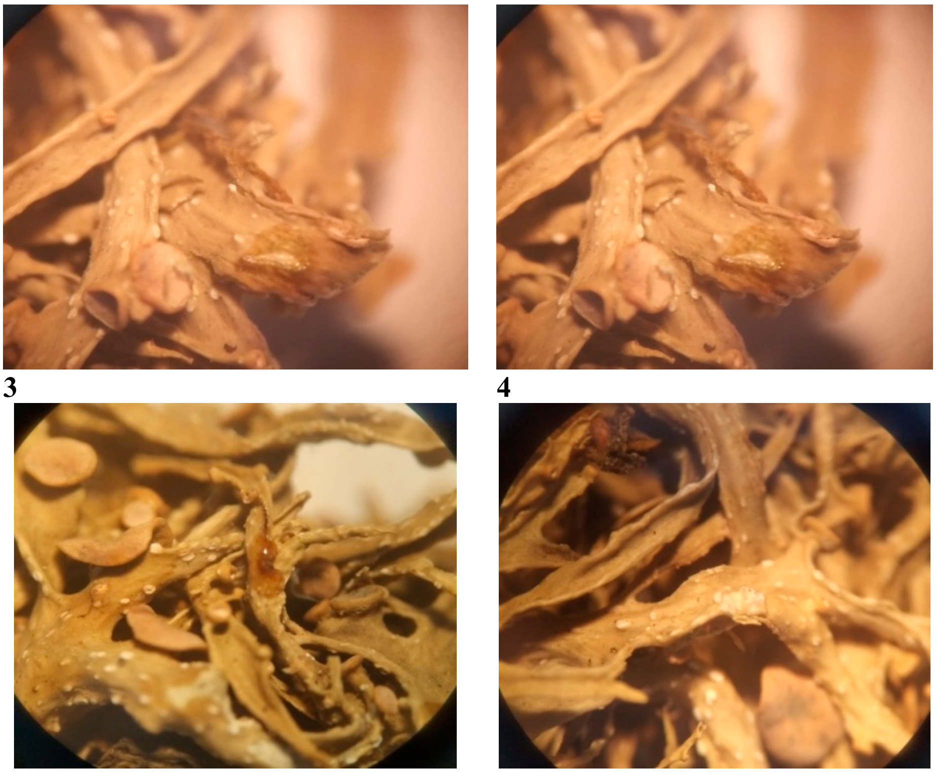

Figure S2: The spot tests for identification of *Ramalinaconduplicans.* 1 = after K test (pale yellow), 2 = after C test (negative), 3 = after KC test (negative), 4 = after P test (negative).

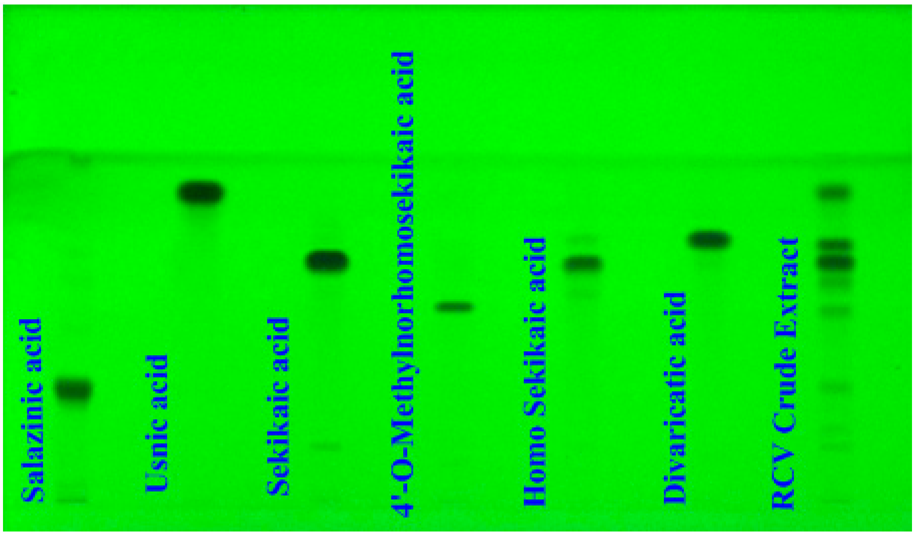

Figure S3: Depicts the HPTLC profiles for *R. conduplicans* extract along the standard compounds at 254 nm, TLC system: toluene/dioxane/acetic acid (72.50:24.20:3.30).

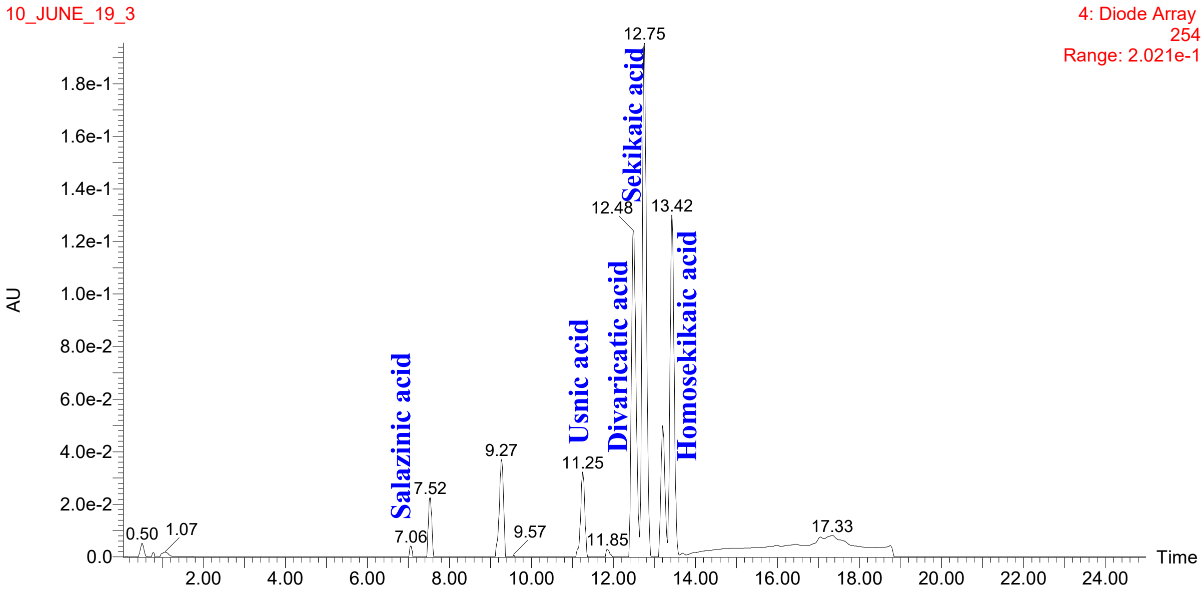

Figure S4: UPLC-PDA chromatogram of acetone extract of *R. conduplicans.*

A)

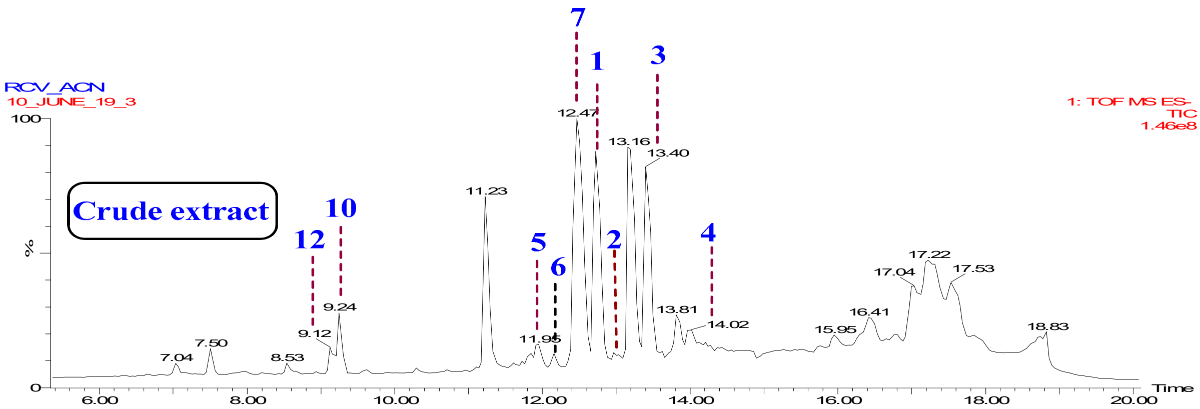
RCV_ACN
10_JUNE_19_3
1: TOF MS ES-
TIC
1.46e8
Crude extract
100
%
0
7
1
3
12
10
5
6
2
4
7.04
7.50
8.53
9.12
9.24
11.23
11.95
12.47
13.16
13.40
13.81
14.02
15.95
16.41
17.04
17.22
17.53
18.83
6.00
8.00
10.00
12.00
14.00
16.00
18.00
20.00
Time

B)

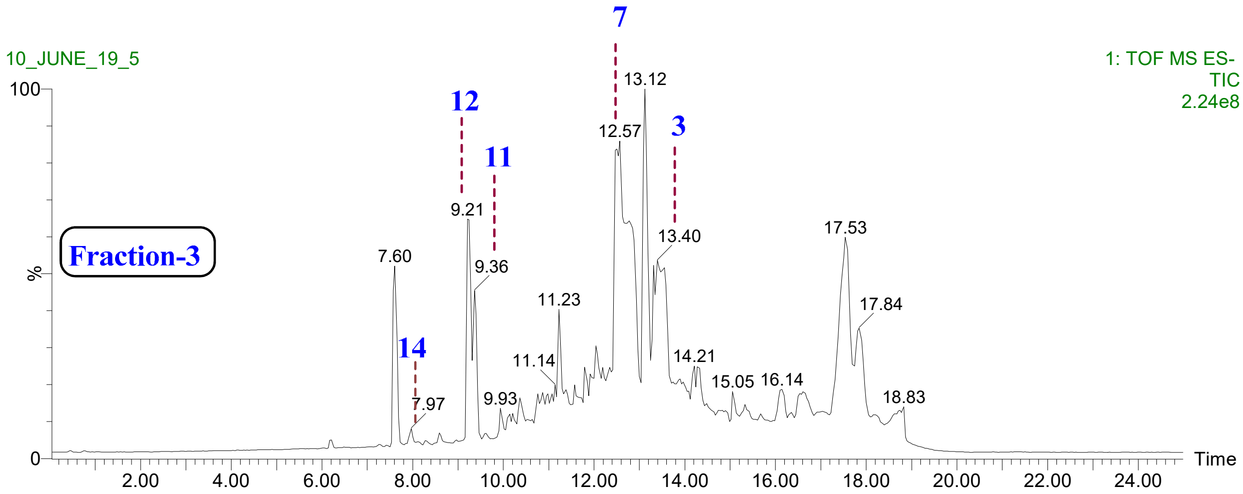
10_JUNE_19_5
1: TOF MS ES-
TIC
2.24e8
Fraction-3
100
%
0
7
12
11
3
14
7.60
7.97
9.21
9.36
9.93
11.14
11.23
12.57
13.12
13.40
14.21
15.05
16.14
17.53
17.84
18.83
2.00
4.00
6.00
8.00
10.00
12.00
14.00
16.00
18.00
20.00
22.00
24.00
Time

C)

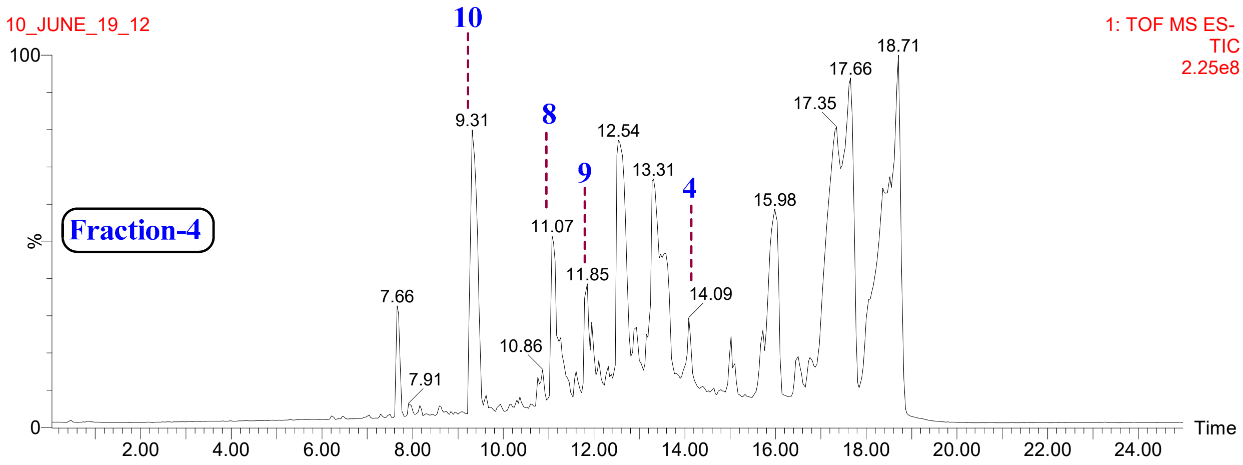
10_JUNE_19_12
1: TOF MS ES-
TIC
2.25e8
Fraction-4
100
%
0
10
8
9
4
7.66
7.91
9.31
10.86
11.07
11.85
12.54
13.31
14.09
15.98
17.35
17.66
18.71
2.00
4.00
6.00
8.00
10.00
12.00
14.00
16.00
18.00
20.00
22.00
24.00
Time

D)

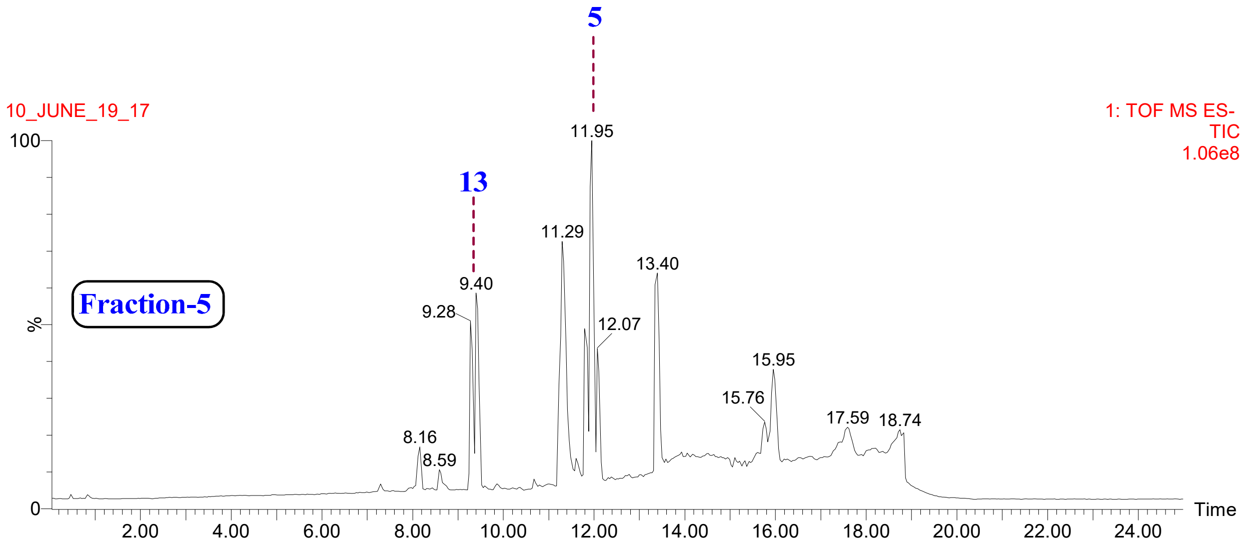
5
13
10_JUNE_19_17
1: TOF MS ES-
TIC
1.06e8
Fraction-5
100
%
0
11.95
11.29
13.40
9.40
9.28
12.07
15.95
15.76
17.59
18.74
8.16
8.59
Time
2.00
4.00
6.00
8.00
10.00
12.00
14.00
16.00
18.00
20.00
22.00
24.00

E)

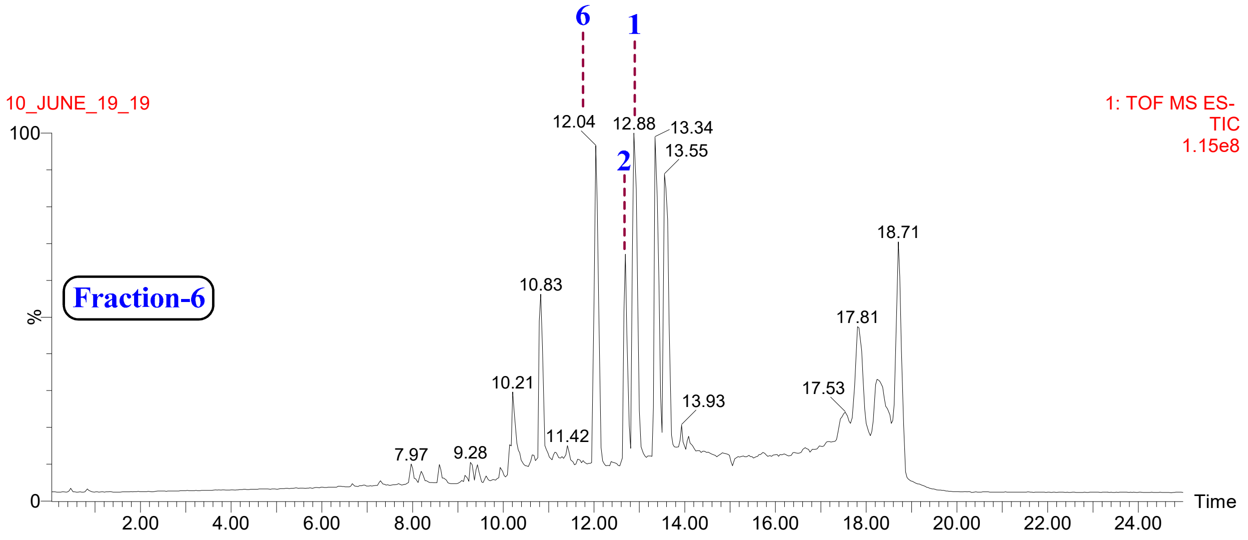
6
1
2
10_JUNE_19_19
1: TOF MS ES-
TIC
1.15e8
Fraction-6
100
%
0
12.04
12.88
13.34
13.55
18.71
10.83
17.81
10.21
17.53
13.93
11.42
7.97
9.28
Time
2.00
4.00
6.00
8.00
10.00
12.00
14.00
16.00
18.00
20.00
22.00
24.00

F)

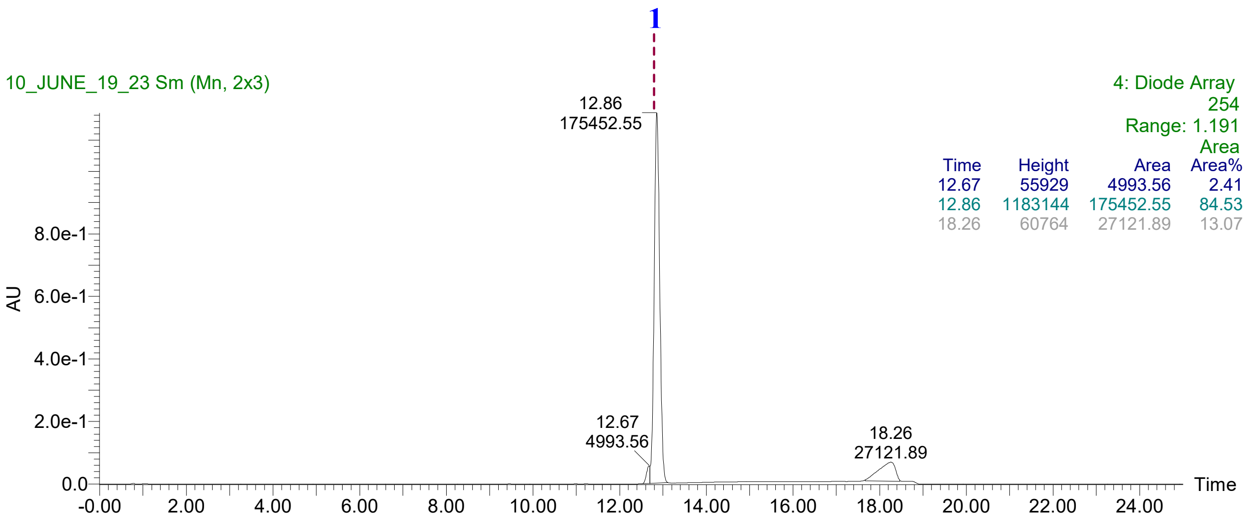
1
10_JUNE_19_23 Sm (Mn, 2x3)
4: Diode Array
254
Range: 1.191
Area
12.86
175452.55
Time Height Area Area%
12.67 55929 4993.56 2.41
12.86 1183144 175452.55 84.53
18.26 60764 27121.89 13.07
AU
8.0e-1
6.0e-1
4.0e-1
2.0e-1
0.0
12.67
4993.56
18.26
27121.89
Time
-0.00
2.00
4.00
6.00
8.00
10.00
12.00
14.00
16.00
18.00
20.00
22.00
24.00

G)

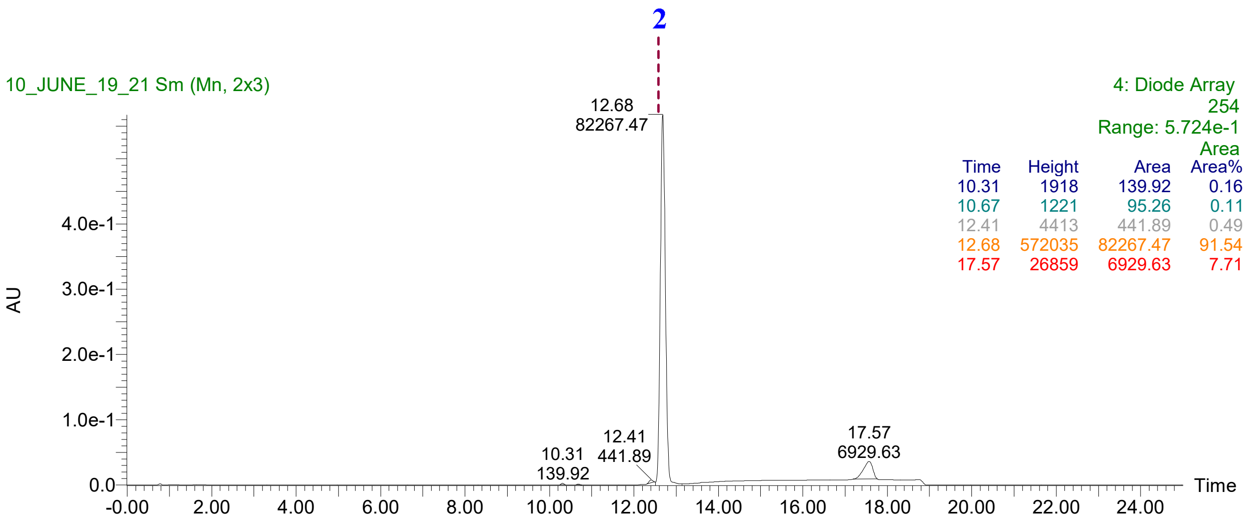
2
10_JUNE_19_21 Sm (Mn, 2x3)
4: Diode Array
254
Range: 5.724e-1
Area
Time Height Area Area%
10.31 1918 139.92 0.16
10.67 1221 95.26 0.11
12.41 4413 441.89 0.49
12.68 572035 82267.47 91.54
17.57 26859 6929.63 7.71
12.68
82267.47
12.41
441.89
10.31
139.92
17.57
6929.63
AU
4.0e-1
3.0e-1
2.0e-1
1.0e-1
0.0
-0.00
2.00
4.00
6.00
8.00
10.00
12.00
14.00
16.00
18.00
20.00
22.00
24.00
Time

H)

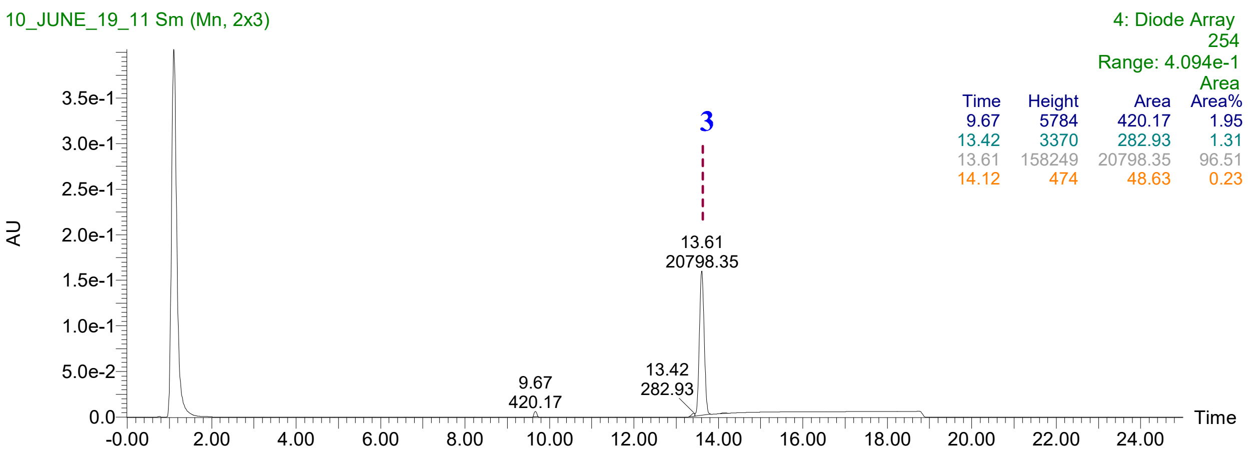
10_JUNE_19_11 Sm (Mn, 2x3)
4: Diode Array
254
Range: 4.094e-1
Area
Time Height Area Area%
9.67 5784 420.17 1.95
13.42 3370 282.93 1.31
13.61 158249 20798.35 96.51
14.12 474 48.63 0.23
3
13.61
20798.35
13.42
282.93
9.67
420.17
AU
3.5e-1
3.0e-1
2.5e-1
2.0e-1
1.5e-1
1.0e-1
5.0e-2
0.0
-0.00
2.00
4.00
6.00
8.00
10.00
12.00
14.00
16.00
18.00
20.00
22.00
24.00
Time

I)

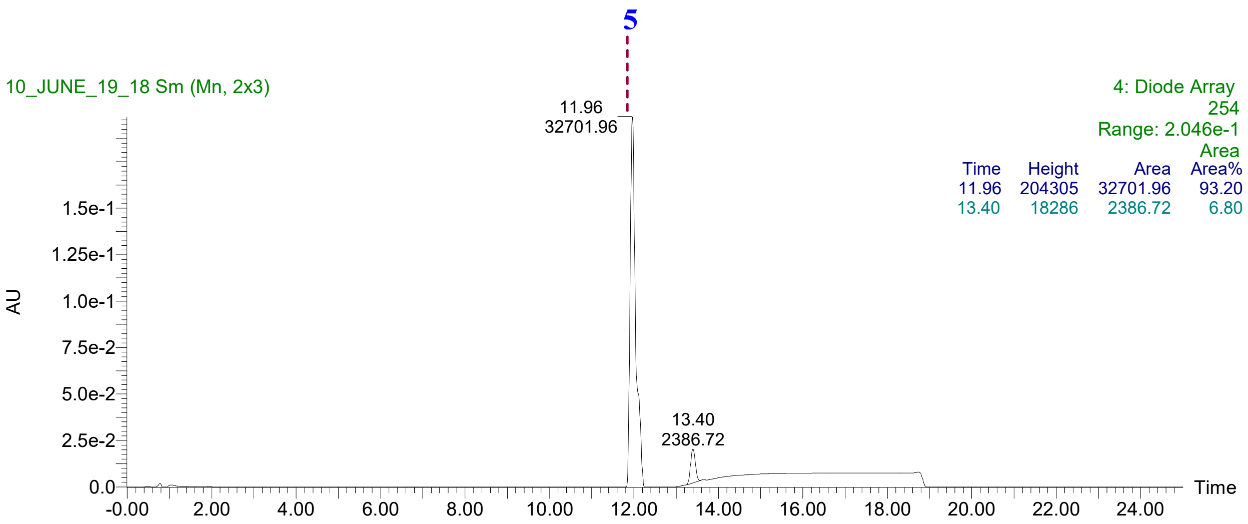
5
10_JUNE_19_18 Sm (Mn, 2x3)
4: Diode Array
254
Range: 2.046e-1
Area
Time Height Area Area%
11.96 204305 32701.96 93.20
13.40 18286 2386.72 6.80
11.96
32701.96
13.40
2386.72
AU
1.5e-1
1.25e-1
1.0e-1
7.5e-2
5.0e-2
2.5e-2
0.0
-0.00
2.00
4.00
6.00
8.00
10.00
12.00
14.00
16.00
18.00
20.00
22.00
24.00
Time

J)

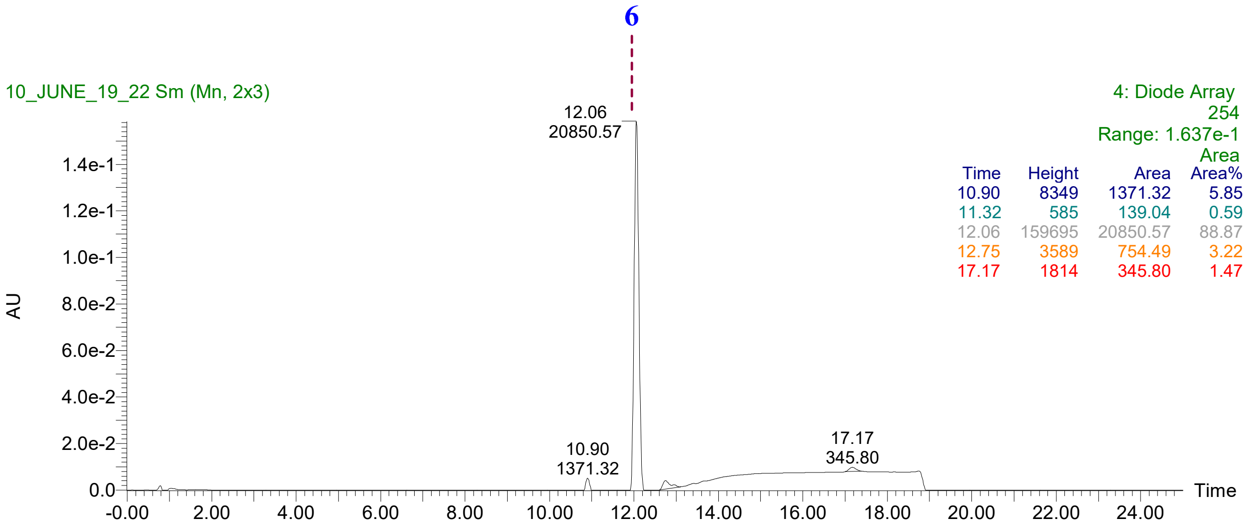
6
10_JUNE_19_22 Sm (Mn, 2x3)
4: Diode Array
254
Range: 1.637e-1
Area
Time Height Area Area%
10.90 8349 1371.32 5.85
11.32 585 139.04 0.59
12.06 159695 20850.57 88.87
12.75 3589 754.49 3.22
17.17 1814 345.80 1.47
12.06
20850.57
10.90
1371.32
17.17
345.80
AU
Time

K)

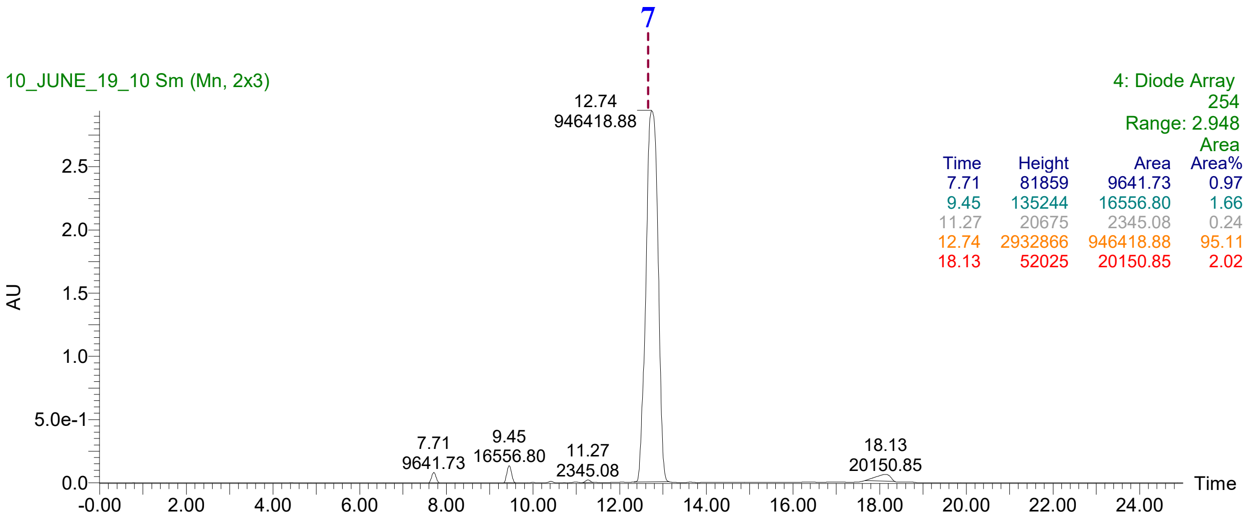
7
10_JUNE_19_10 Sm (Mn, 2x3)
4: Diode Array
254
Range: 2.948
Area
Time Height Area Area%
7.71 81859 9641.73 0.97
9.45 135244 16556.80 1.66
11.27 20675 2345.08 0.24
12.74 2932866 946418.88 95.11
18.13 52025 20150.85 2.02
12.74
946418.88
7.71
9641.73
9.45
16556.80
11.27
2345.08
18.13
20150.85
AU
Time

L)

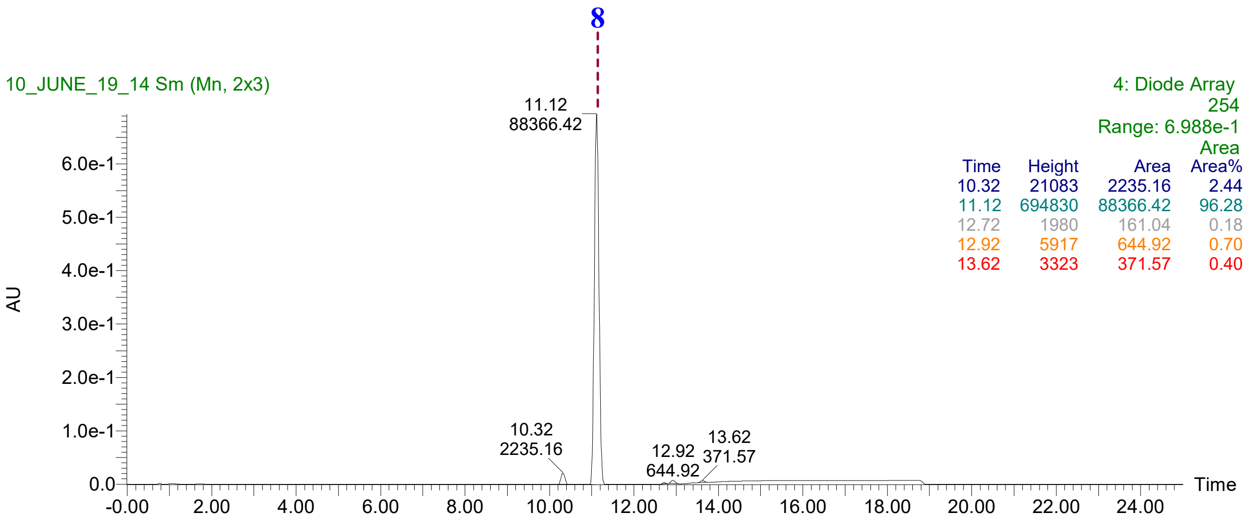
8
10_JUNE_19_14 Sm (Mn, 2x3)
4: Diode Array
254
Range: 6.988e-1
Area
Time Height Area Area%
10.32 21083 2235.16 2.44
11.12 694830 88366.42 96.28
12.72 1980 161.04 0.18
12.92 5917 644.92 0.70
13.62 3323 371.57 0.40
11.12
88366.42
10.32
2235.16
12.92
644.92
13.62
371.57
AU
Time

M)

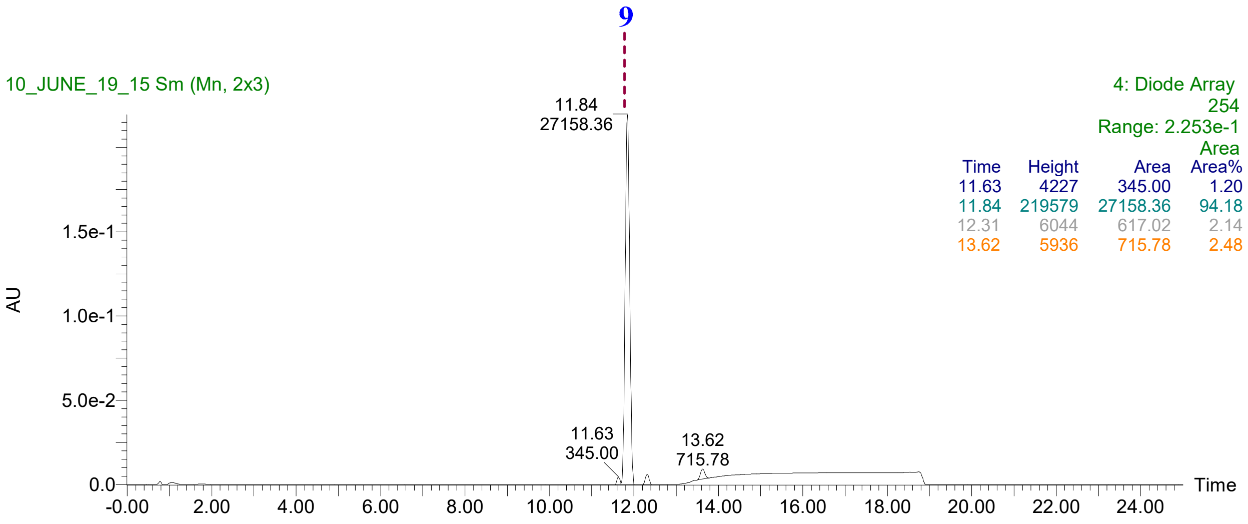
9
10_JUNE_19_15 Sm (Mn, 2x3)
4: Diode Array
254
Range: 2.253e-1
Area
Time Height Area Area%
11.63 4227 345.00 1.20
11.84 219579 27158.36 94.18
12.31 6044 617.02 2.14
13.62 5936 715.78 2.48
11.84
27158.36
11.63
345.00
13.62
715.78
AU
1.5e-1
1.0e-1
5.0e-2
0.0
-0.00 2.00 4.00 6.00 8.00 10.00 12.00 14.00 16.00 18.00 20.00 22.00 24.00
Time

N)

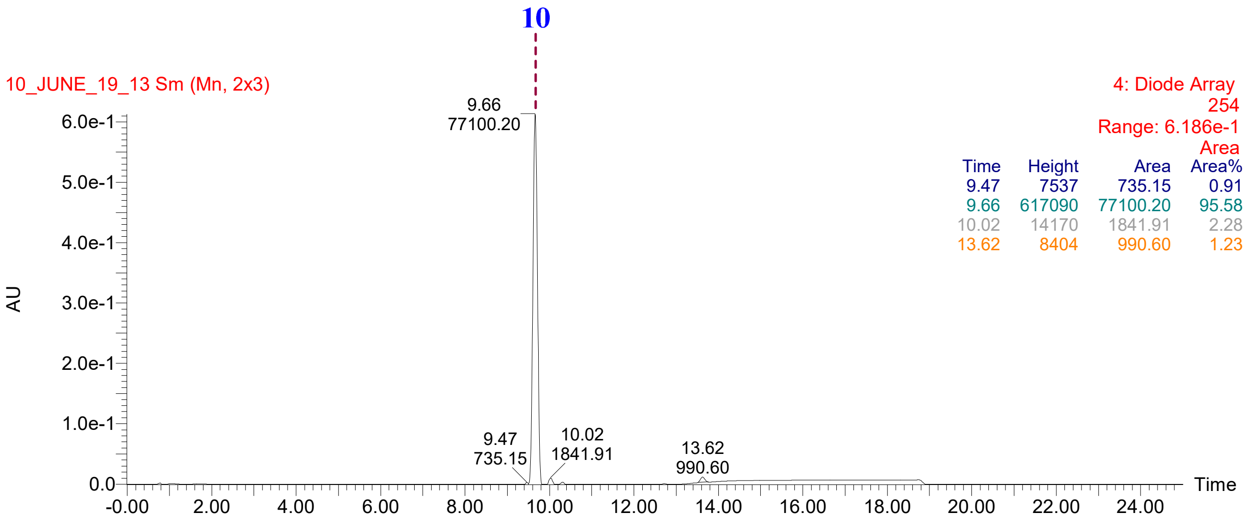
10
10_JUNE_19_13 Sm (Mn, 2x3)
4: Diode Array
254
Range: 6.186e-1
Area
Time Height Area Area%
9.47 7537 735.15 0.91
9.66 617090 77100.20 95.58
10.02 14170 1841.91 2.28
13.62 8404 990.60 1.23
9.66
77100.20
9.47
735.15
10.02
1841.91
13.62
990.60
AU
6.0e-1
5.0e-1
4.0e-1
3.0e-1
2.0e-1
1.0e-1
0.0
-0.00 2.00 4.00 6.00 8.00 10.00 12.00 14.00 16.00 18.00 20.00 22.00 24.00
Time

O)

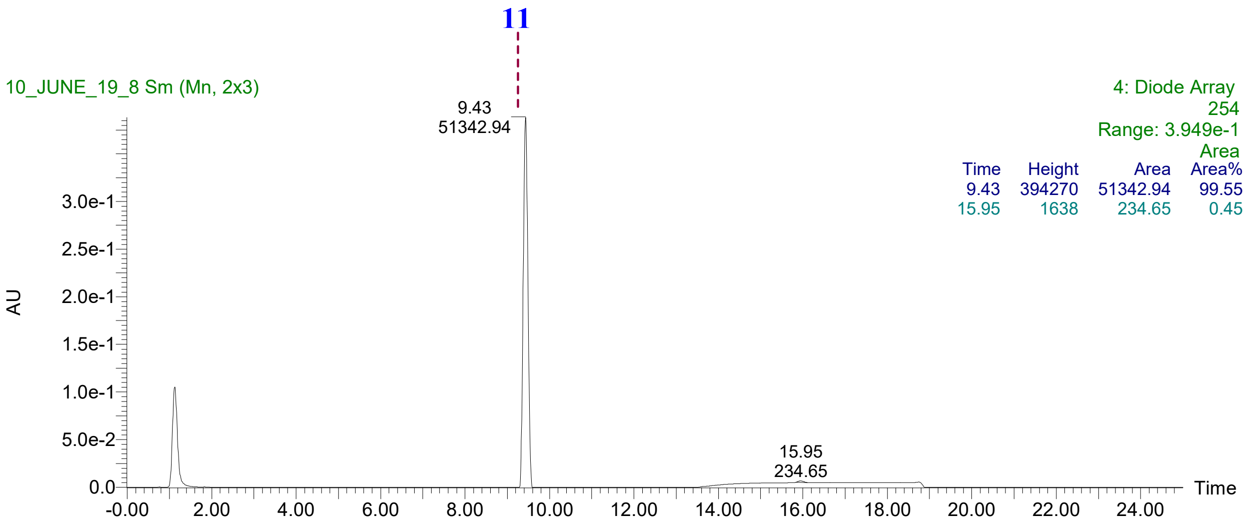
11
10_JUNE_19_8 Sm (Mn, 2x3)
4: Diode Array
254
Range: 3.949e-1
Area
Time Height Area Area%
9.43 394270 51342.94 99.55
15.95 1638 234.65 0.45
9.43
51342.94
15.95
234.65
AU
3.0e-1
2.5e-1
2.0e-1
1.5e-1
1.0e-1
5.0e-2
0.0
-0.00 2.00 4.00 6.00 8.00 10.00 12.00 14.00 16.00 18.00 20.00 22.00 24.00
Time

P)

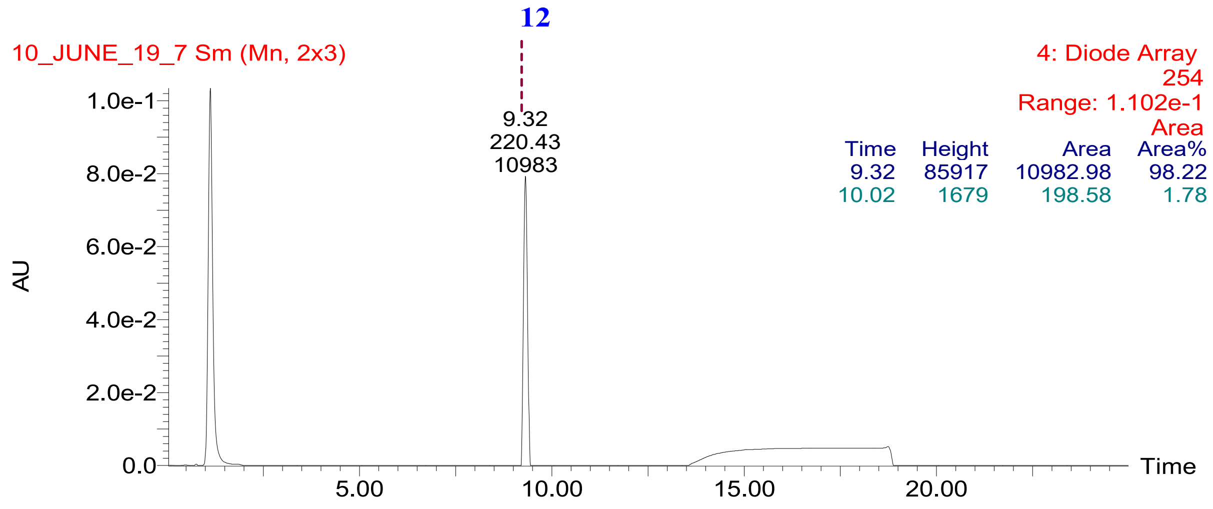

Q)

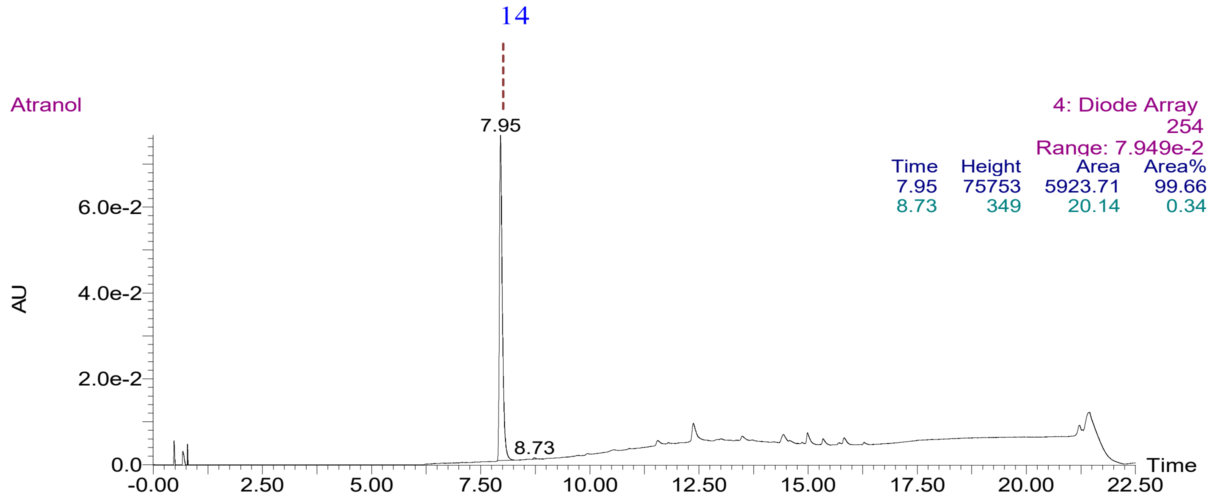

Figure S5: Shows the Total Ion Chromatogram (TIC) of fractions and pure compounds. A = Extract; B = Fraction 3; C = Fraction 4; D = Fraction 5; E = Fraction 6; F = Compound 1; G = Compound 2; H = Compound 3; I = Compound 5; J = Compound 6; K = Compound 7; L = Compound 8; M = Compound 9; N = Compound 10; O = Compound 11; P = Compound 12; Q = Compound 14.

NPL IICT
FTIR REPORT

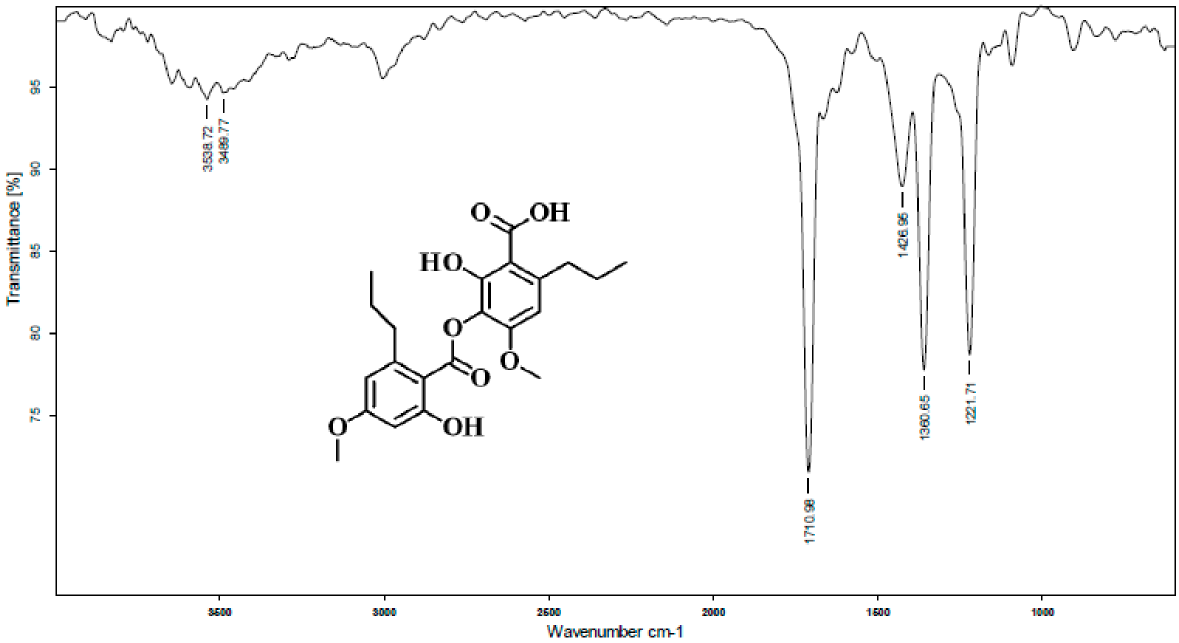

Figure S6: FTIR spectrum of Sekikaic acid (**1**).

	Mass	Calc. Mass	mDa	PPM	DBE	i-FIT	Norm	Conf(%)	Formula
Minimum:					-1.5				
Maximum:			5.0	5.0	50.0				
	417.1553	417.1549	0.4	1.0	10.5	484.1	n/a	n/a	C22 H25 O8

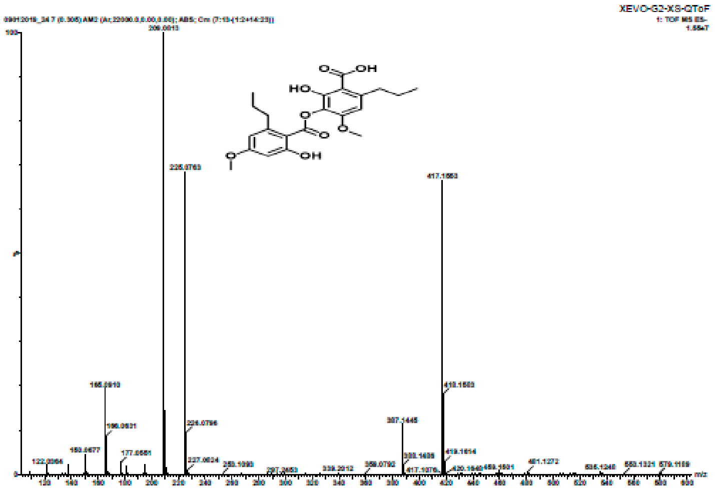

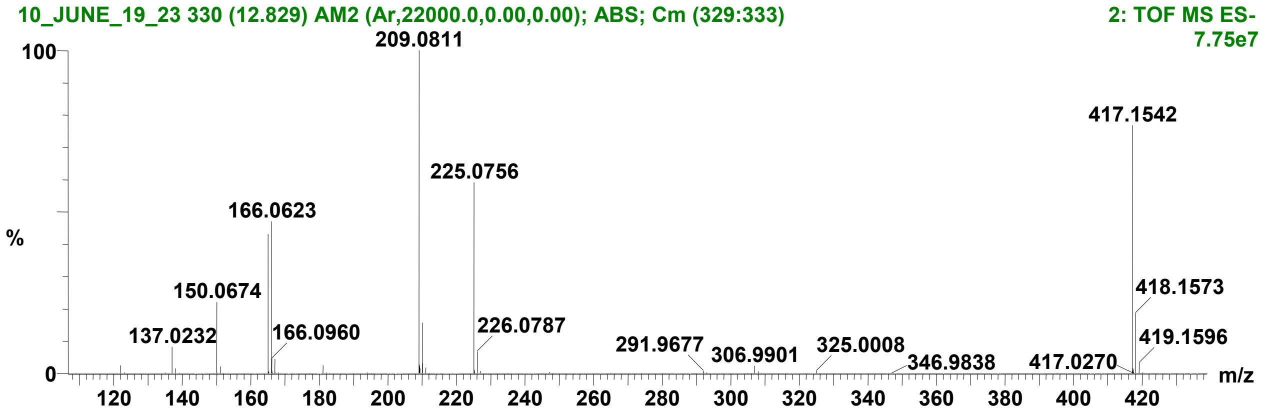

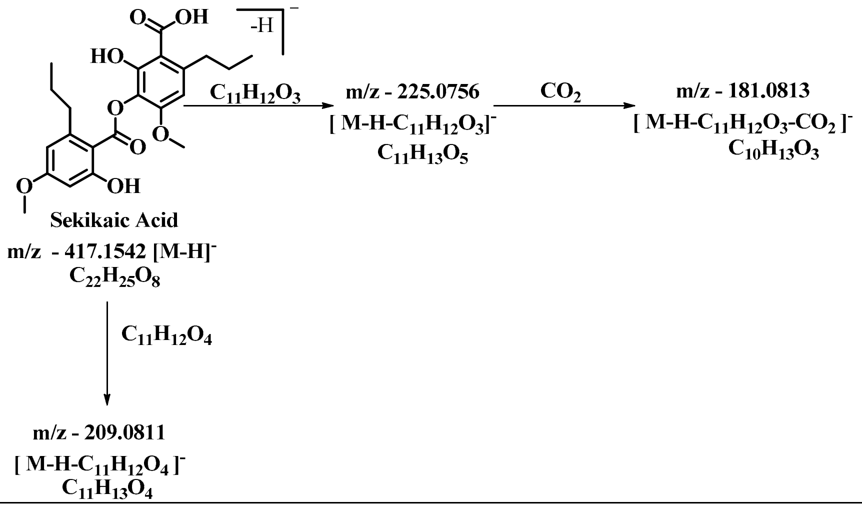

Figure S7: HRESIMS, MS/MS spectra and fragmentation pattern of Sekikaic acid (**1**).

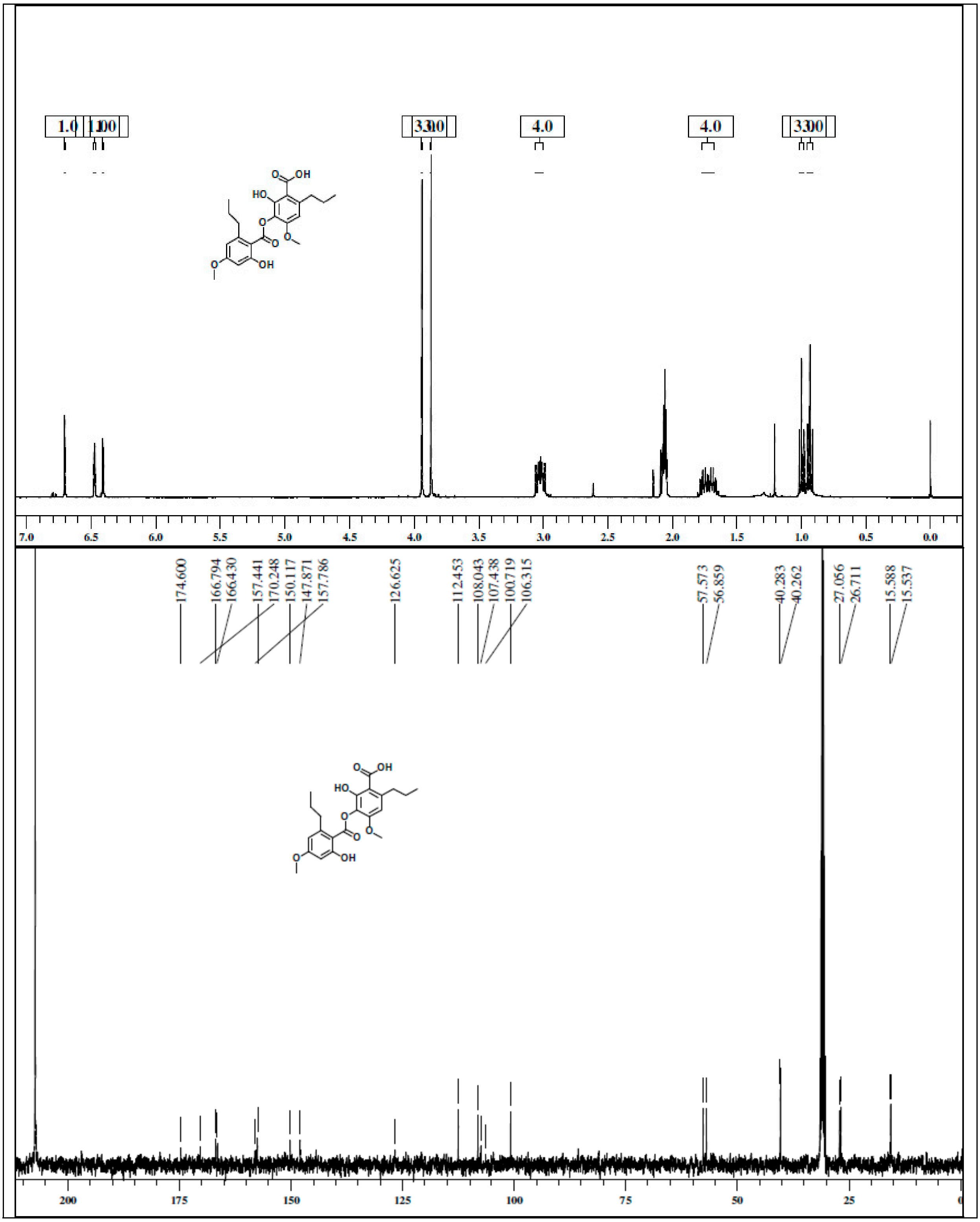

Figure S8: ^{1}H & ^{13}C NMR spectrum of Sekikaic acid (**1**) (400 & 100 MHz, CD_3COCD_3).

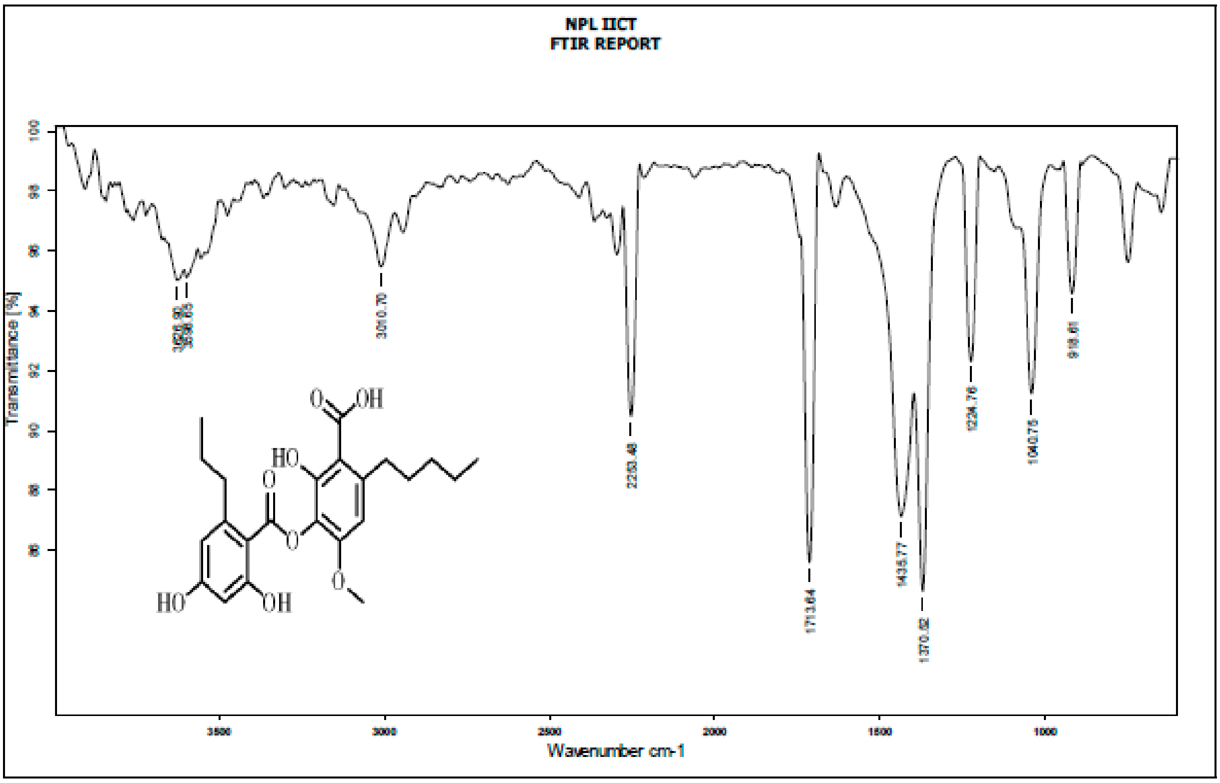

Figure S9: FTIR spectrum of 4’-*O*-Methylnorhomosekikaic acid (**2**).

Minimum:				-1.5				
Maximum:		5.0	5.0	50.0				
Mass	Calc. Mass	mDa	PPM	DBE	i-FIT	Norm	Conf(%)	Formula
431.1706	431.1706	0.0	0.0	10.5	693.9	n/a	n/a	C23 H27 O8

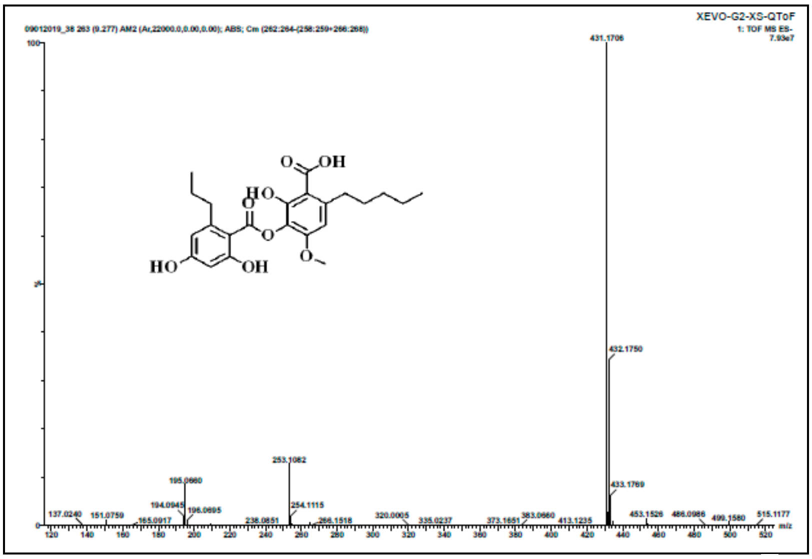

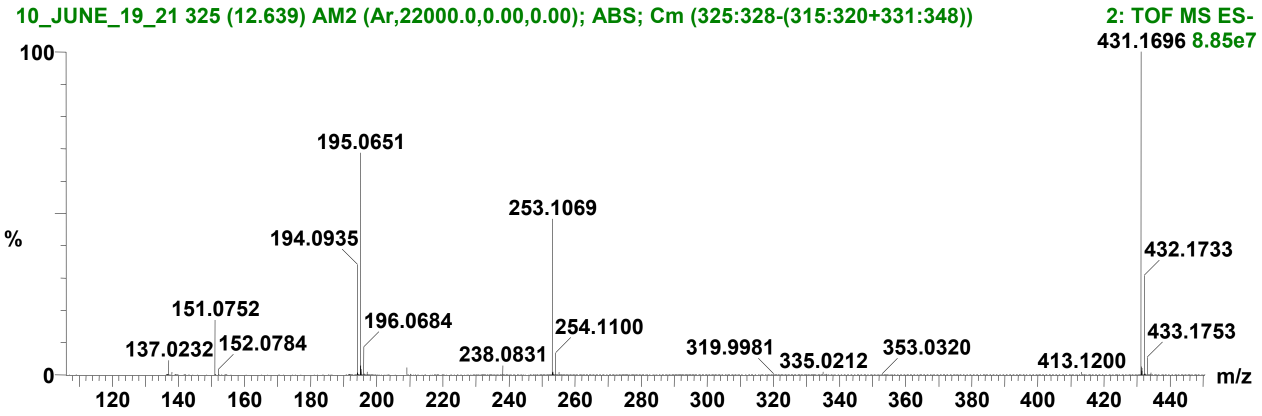

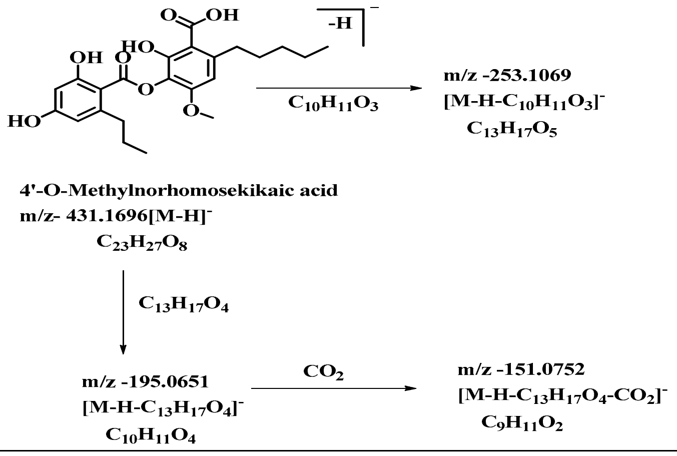

Figure S10: HRESIMS, MS/MS spectra and fragmentation pattern of 4'-O-Methylnorhomosekikaic acid (**2**).

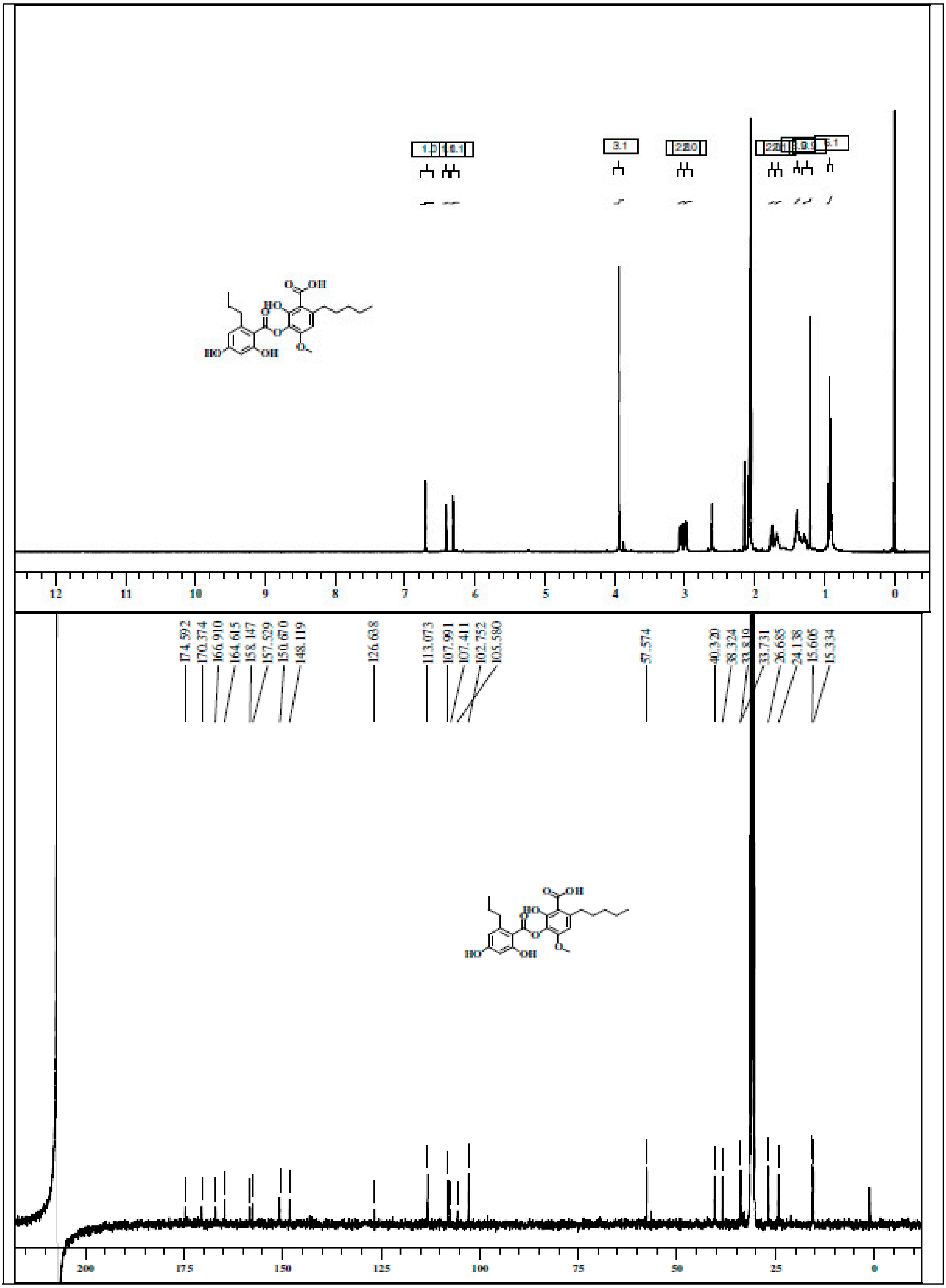

Figure S11: ^{1}H & ^{13}C NMR Spectrum of 4’-O-Methylnorhomosekikaic acid (**2**) (400 & 100 MHz, CD_3COCD_3).

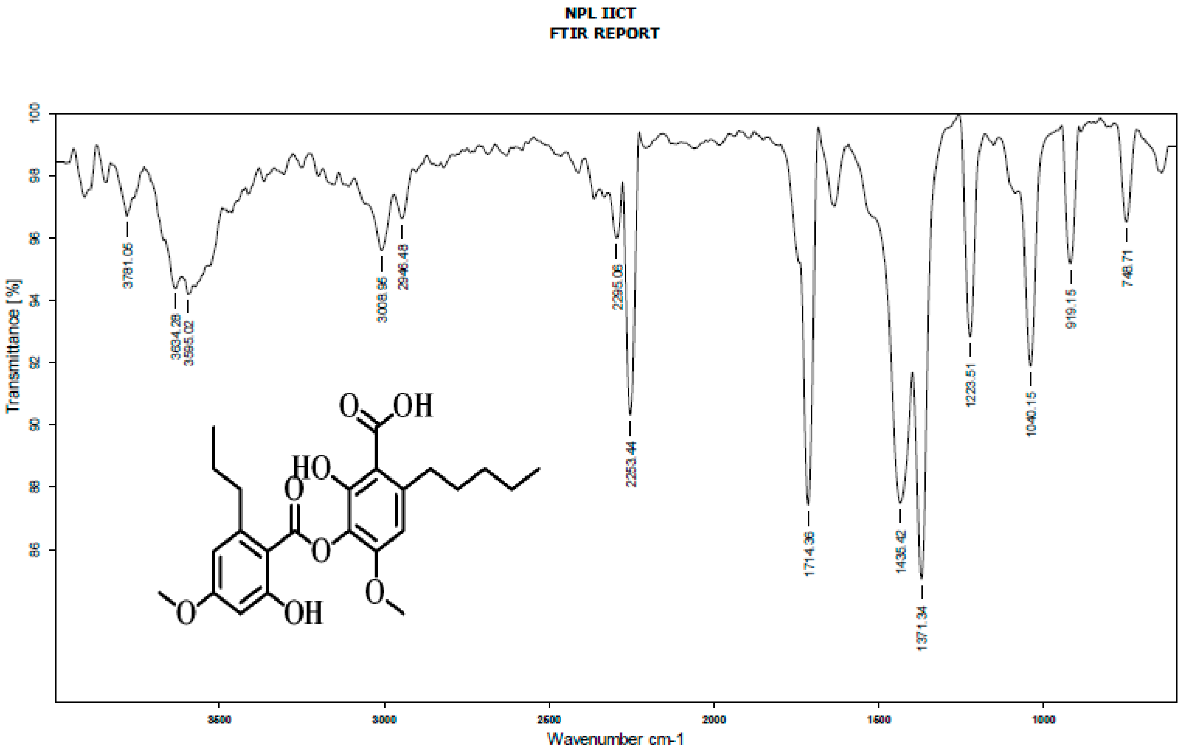

Figure S12: FTIR spectrum of Homosekikaic acid (**3**).

Minimum:				-1.5				
Maximum:		5.0	5.0	50.0				
Mass	Calc. Mass	mDa	PPM	DBE	i-FIT	Norm	Conf(%)	Formula
445.1859	445.1862	-0.3	-0.7	10.5	458.0	n/a	n/a	C24 H29 O8

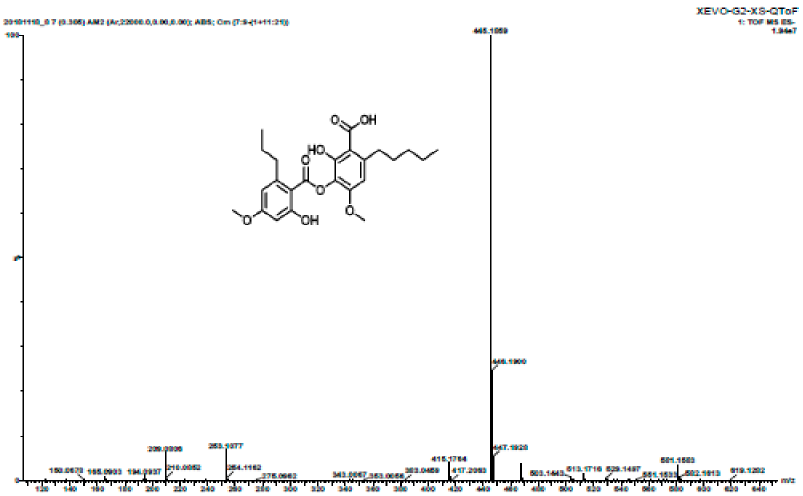

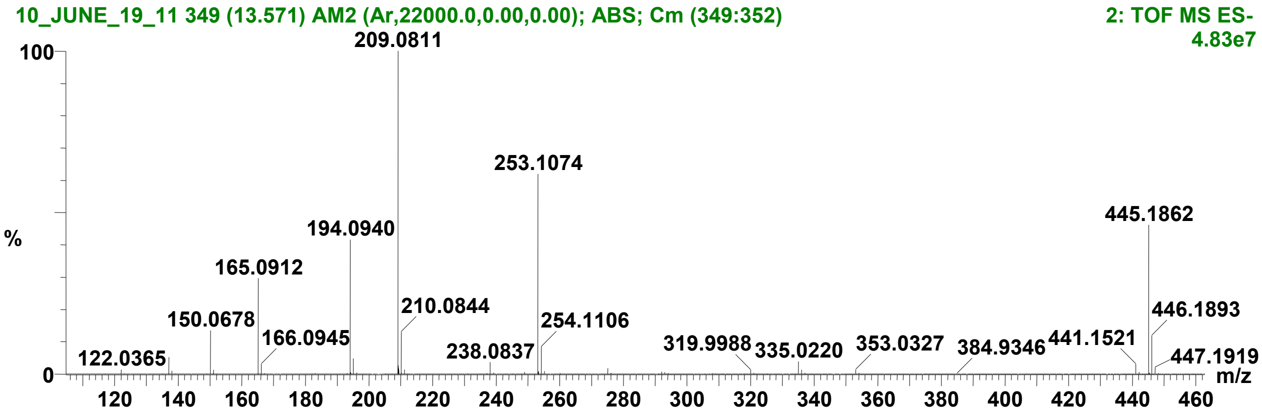

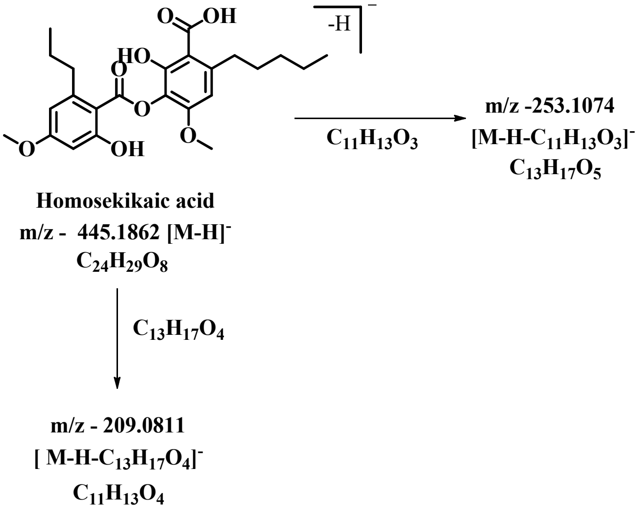

Figure S13: HRESIMS, MS/MS spectra and fragmentation pattern of Homosekikaic acid (**3**).

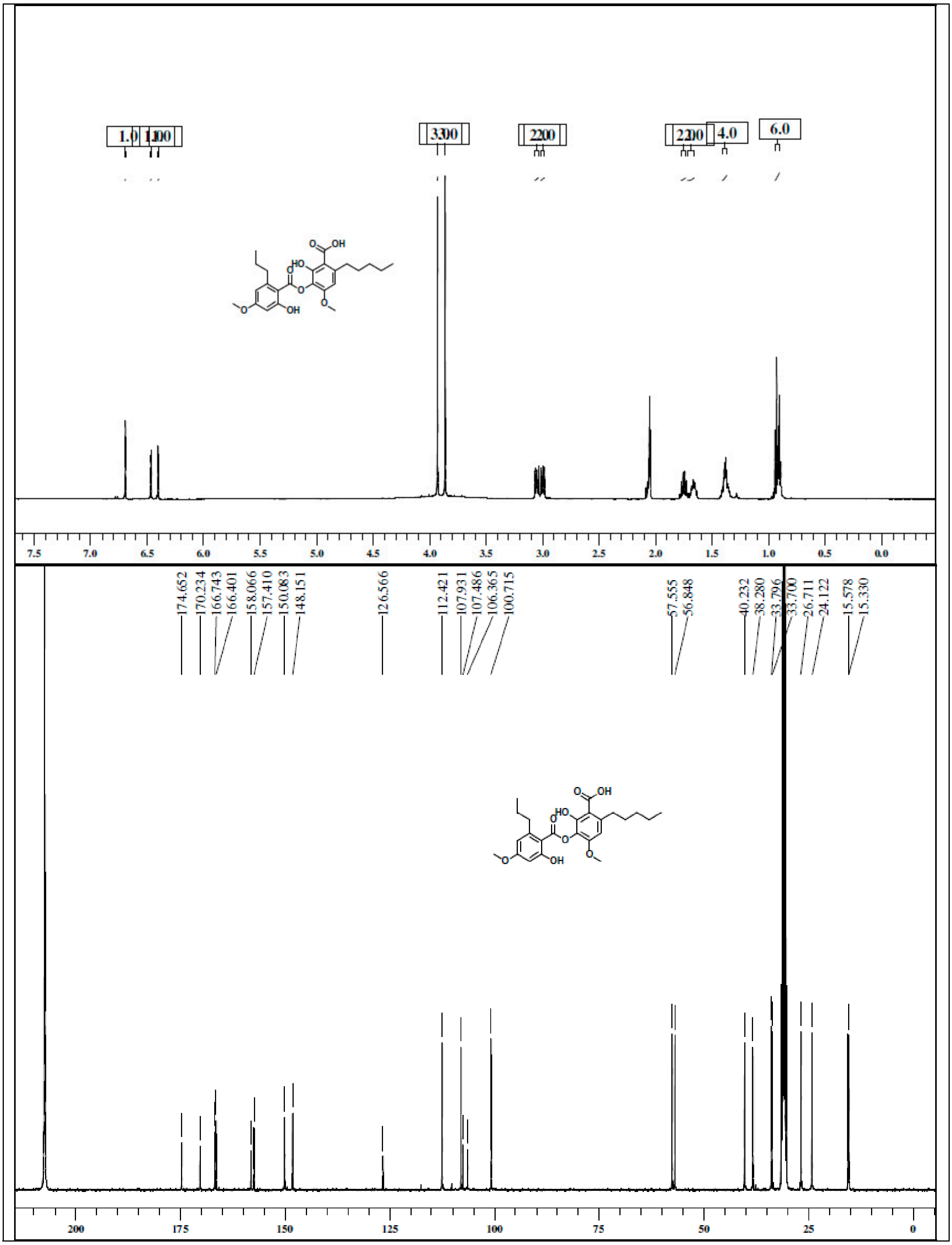

Figure S14: ^{1}H & ^{13}C NMR Spectrum of Homosekikaic acid (**3**) (400 & 100 MHz, CD_3COCD_3).

Minimum:				-1.5				
Maximum:		5.0	10.0	50.0				
Mass	Calc. Mass	mDa	PPM	DBE	i-FIT	Norm	Conf(%)	Formula
473.2176	473.2175	0.1	0.2	10.5	595.0	n/a	n/a	C26 H33 O8

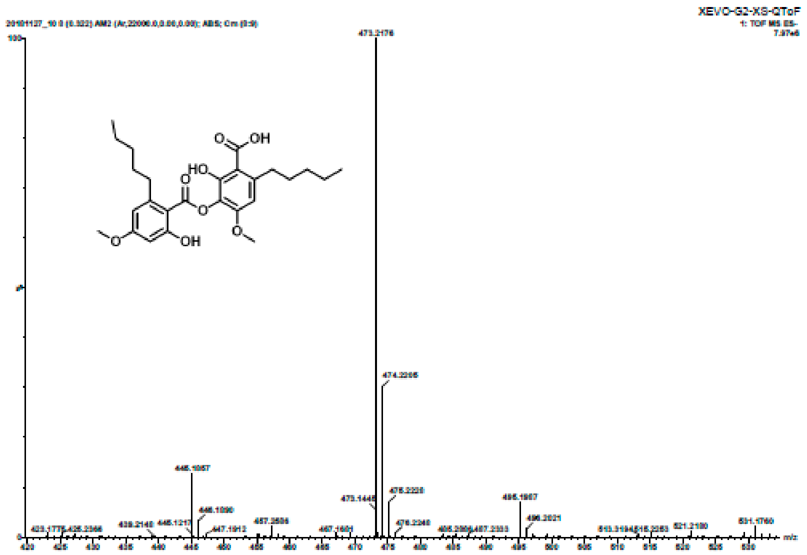

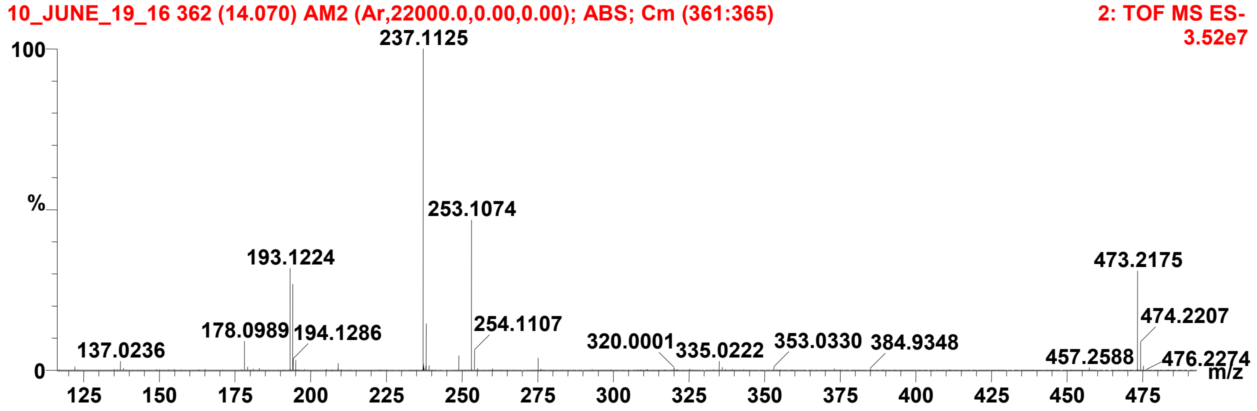

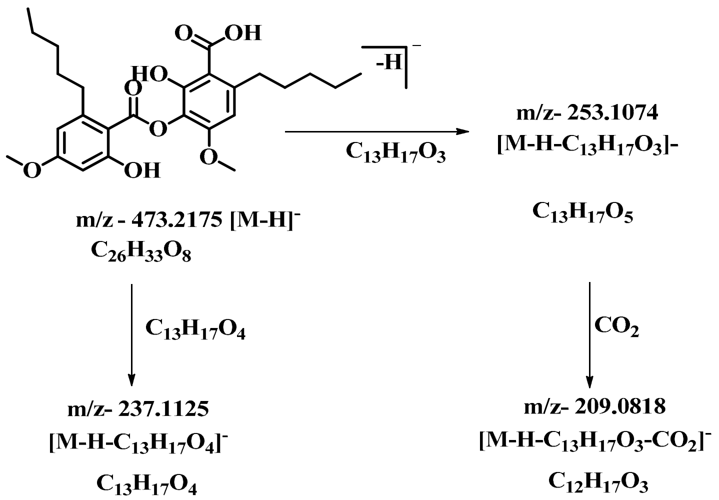

Figure S15: HRESIMS, MS/MS spectra and fragmentation pattern of Hyperhomosekikaic acid (**4**).

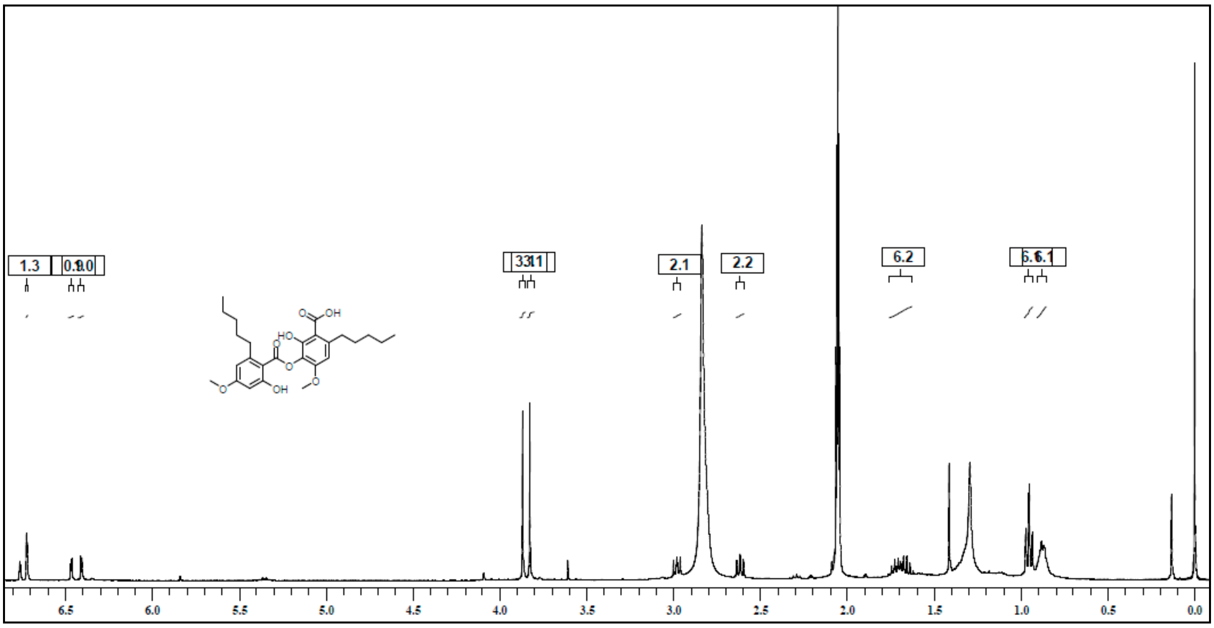

Figure S16: ^{1}H NMR Spectrum of Hyperhomosekikaic acid (**4**) (400 MHz, CD_3COCD_3).

Minimum:				-1.5				
Maximum:		5.0	5.0	50.0				
Mass	Calc. Mass	mDa	PPM	DBE	i-FIT	Norm	Conf(%)	Formula
401.1961	401.1964	-0.3	-0.7	9.5	895.2	n/a	n/a	C23 H29 O6

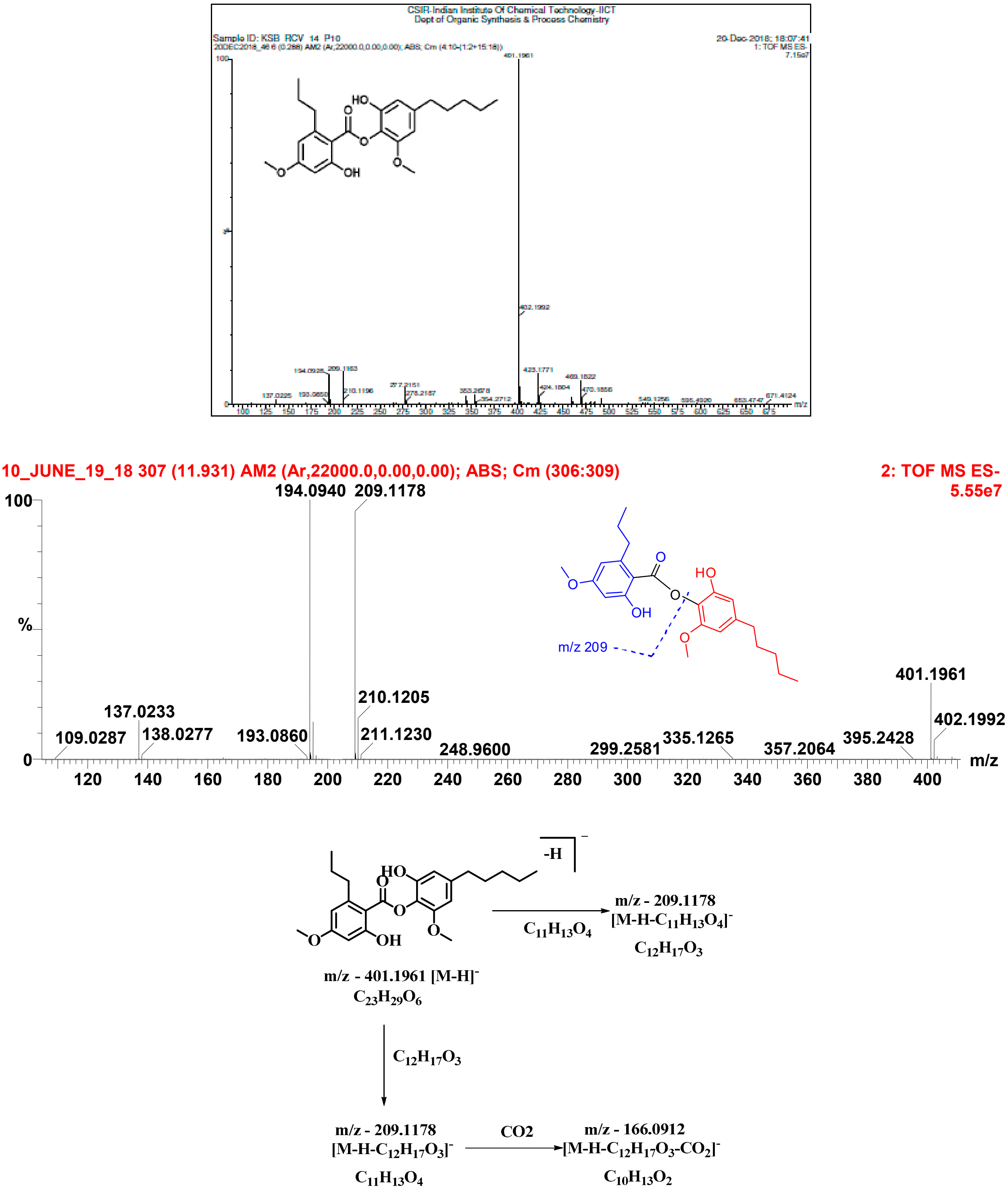

Figure S17: HRESIMS, MS/MS spectra and fragmentation pattern of compound (**5**).

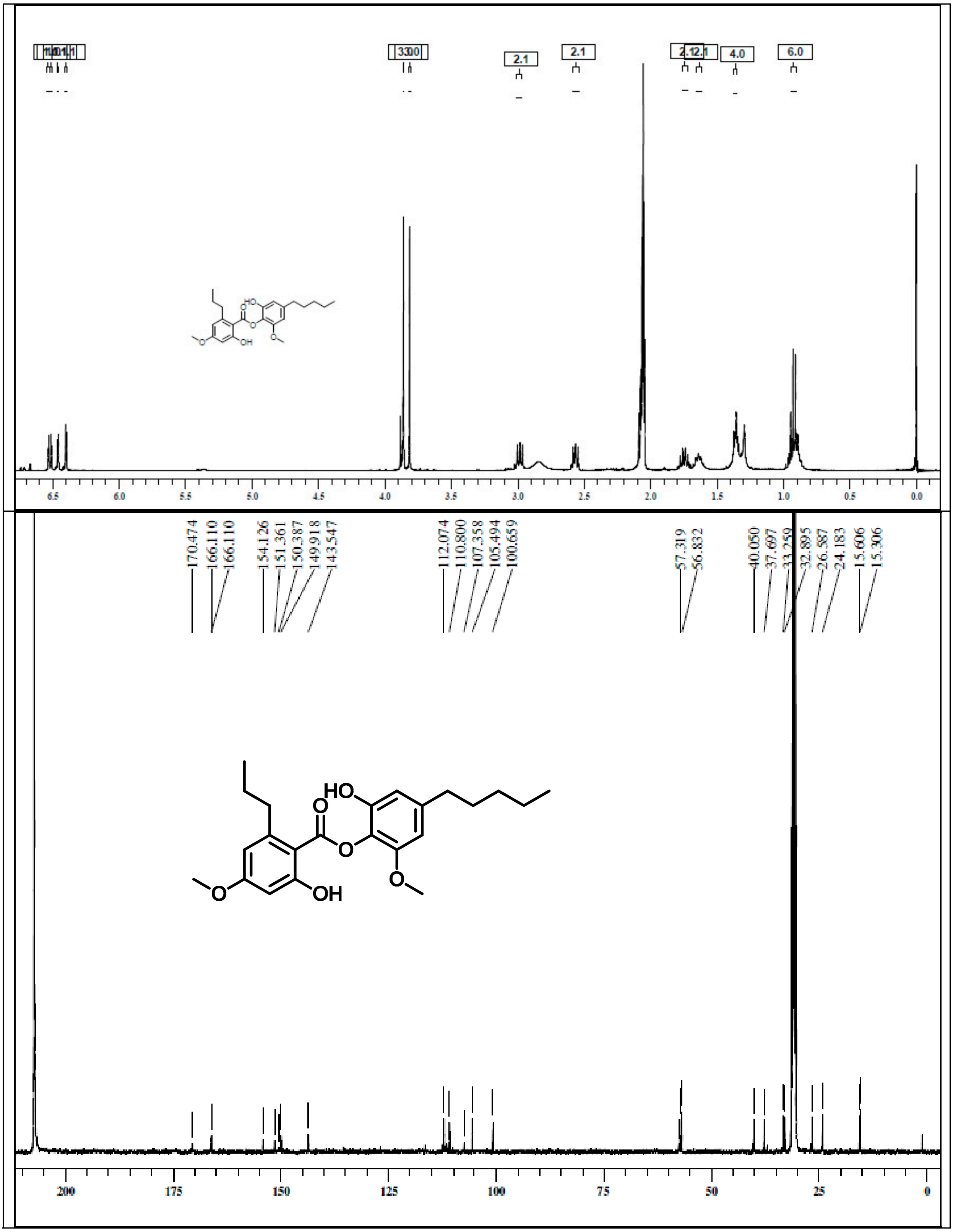

Figure S18: ^{1}H &^{13}C NMR Spectrum of Compound (**5**) (400 & 100 MHz, CD_3COCD_3).

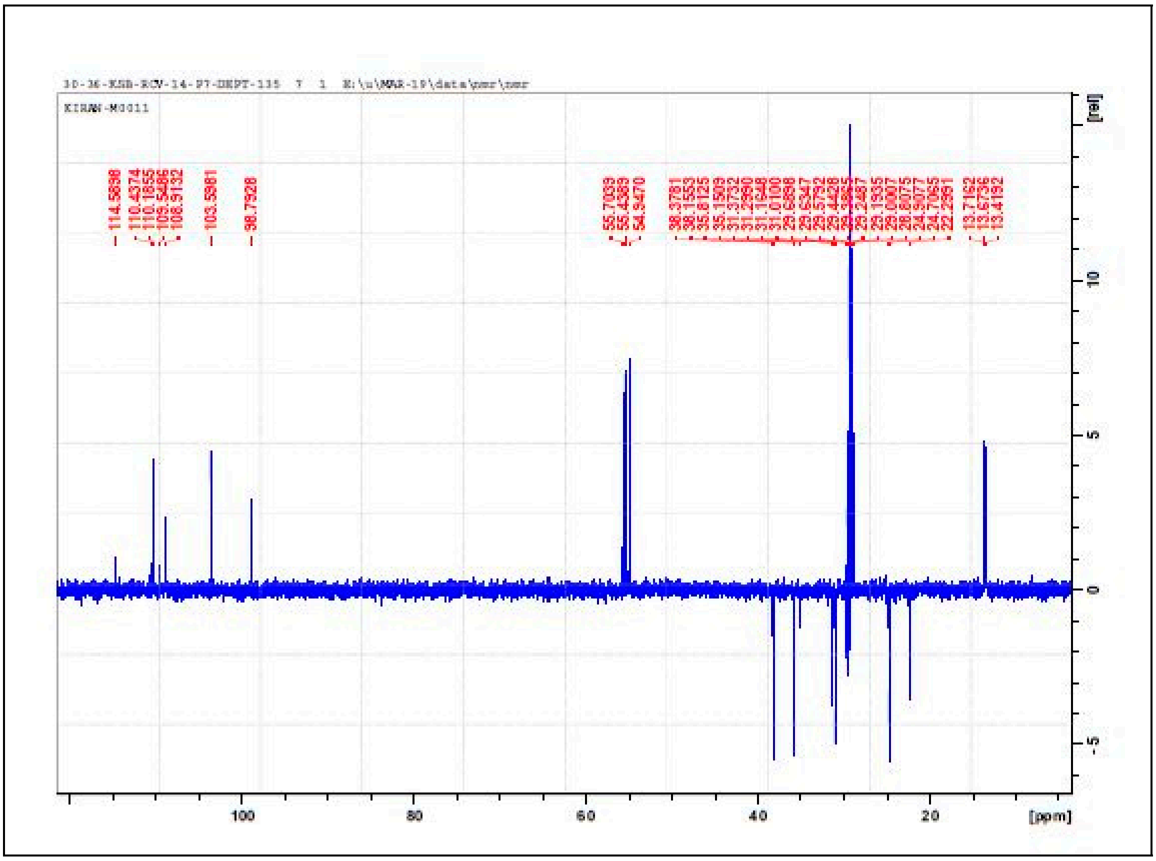

Figure S19: DEPT 135º NMR spectra (CD_3COCD_3) of compound **5**.

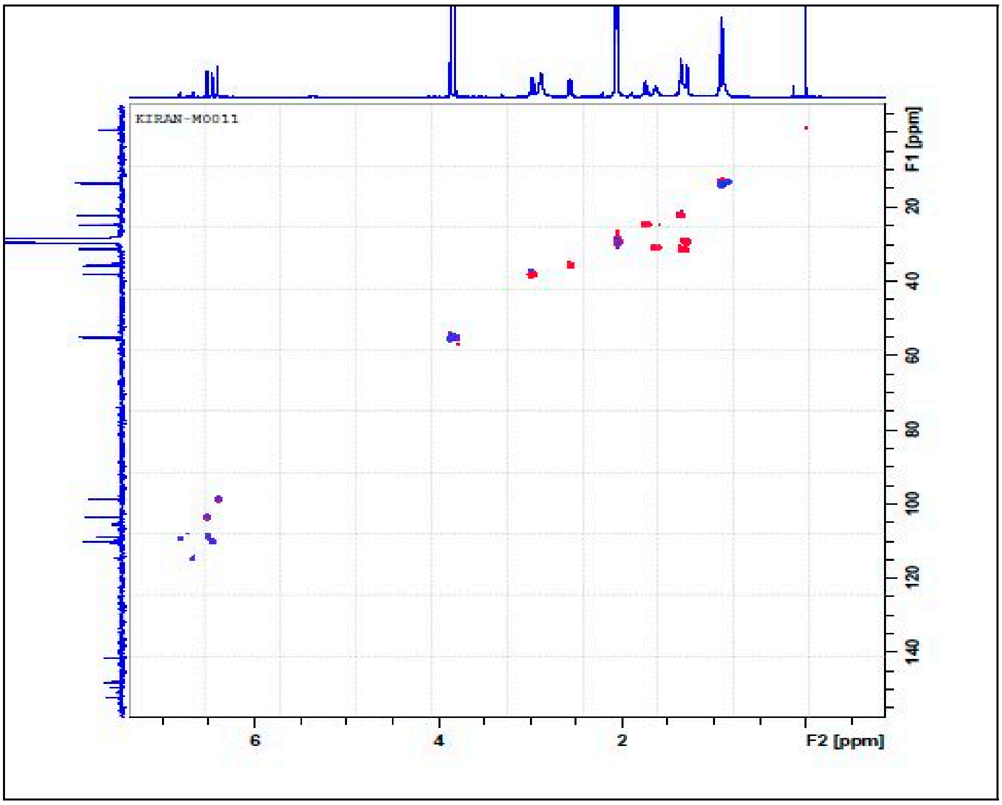

Figure S20: HSQC Spectrum of Compound **5**.

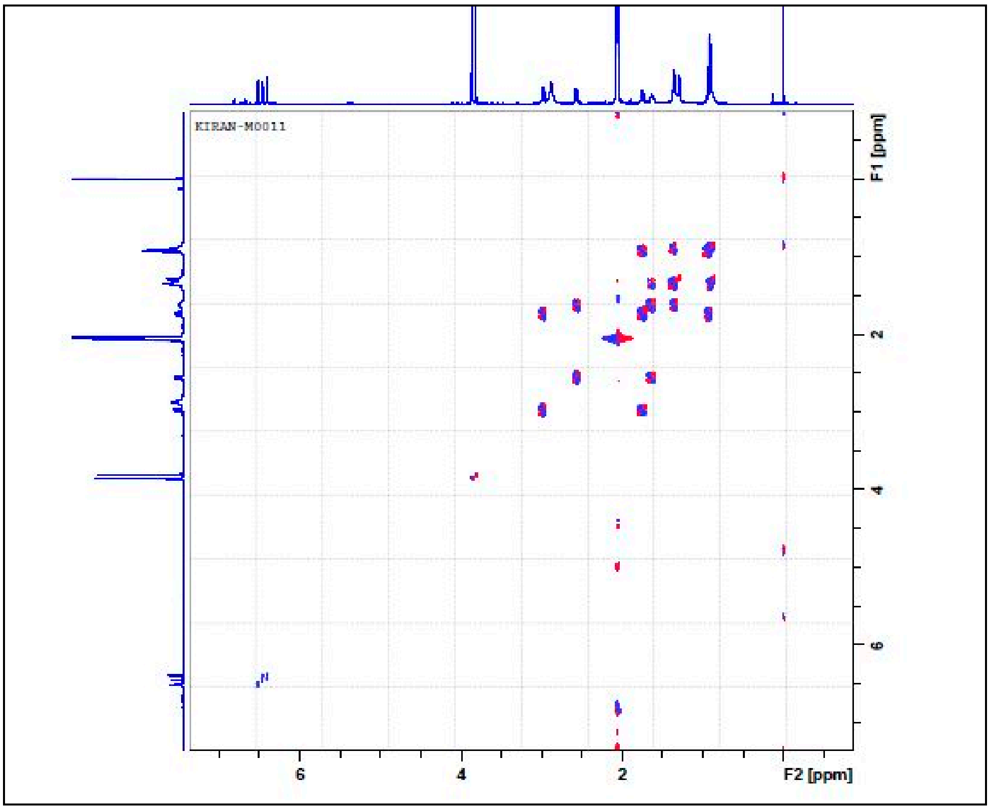

Figure S21: DQF-COSY Spectrum of Compound **5**.

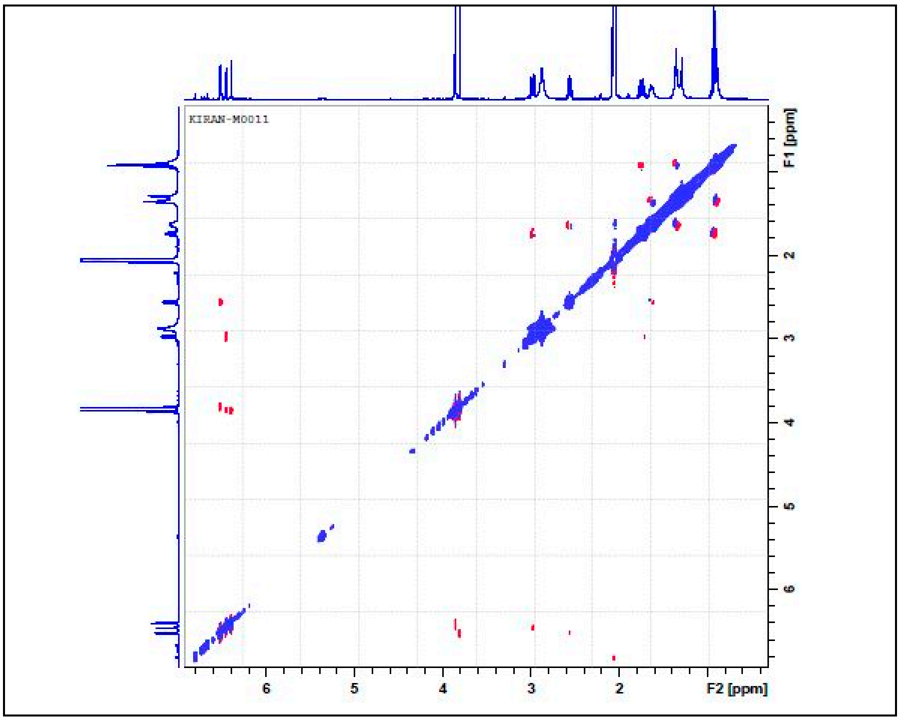

Figure S22: NOESY Spectrum of Compound **5.**

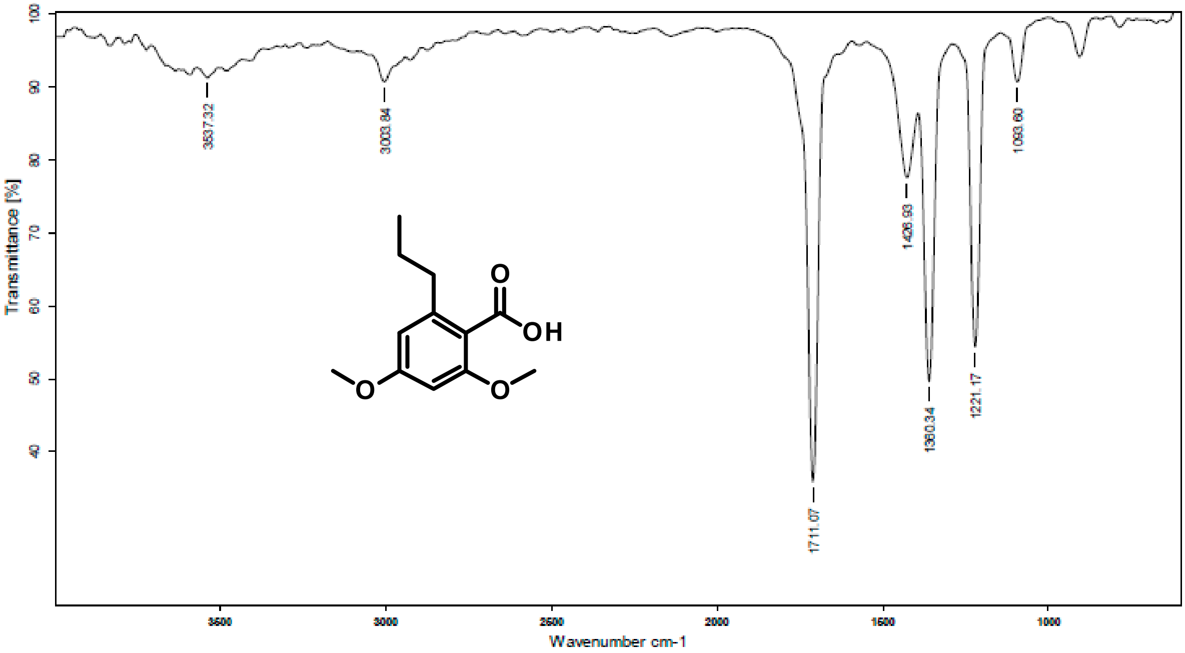

Figure S23: FTIR spectrum of Compound **6**.

Minimum:				-1.5				
Maximum:		5.0	5.0	50.0				
Mass	Calc. Mass	mDa	PPM	DBE	i-FIT	Norm	Conf(%)	Formula
223.0975	223.0970	0.5	2.2	5.5	607.7	n/a	n/a	C12 H15 O4

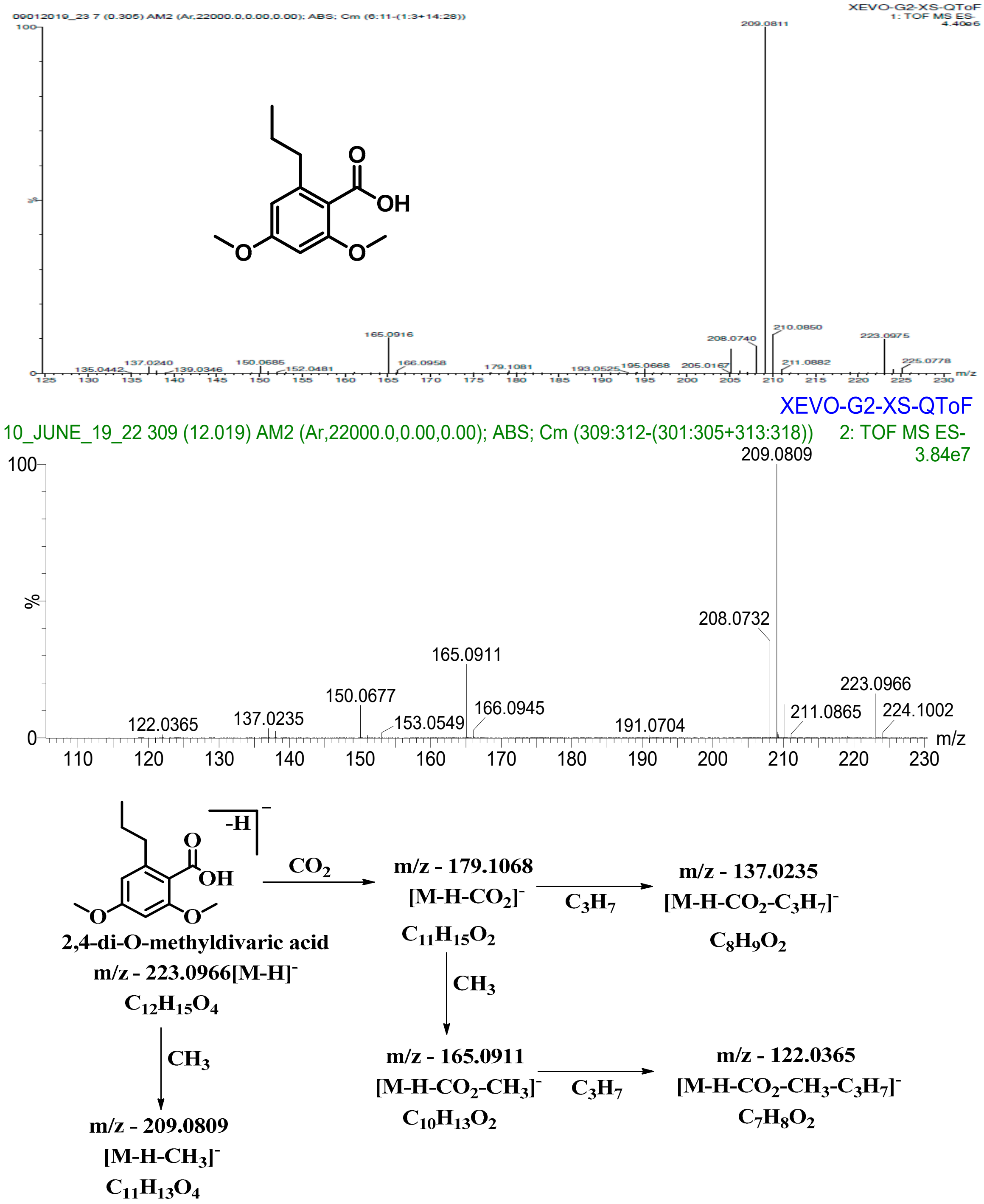

Figure S24: HRESIMS, MS/MS spectra and fragmentation pattern of 2, 4-di-O-Methyldivaic acid (**6**).

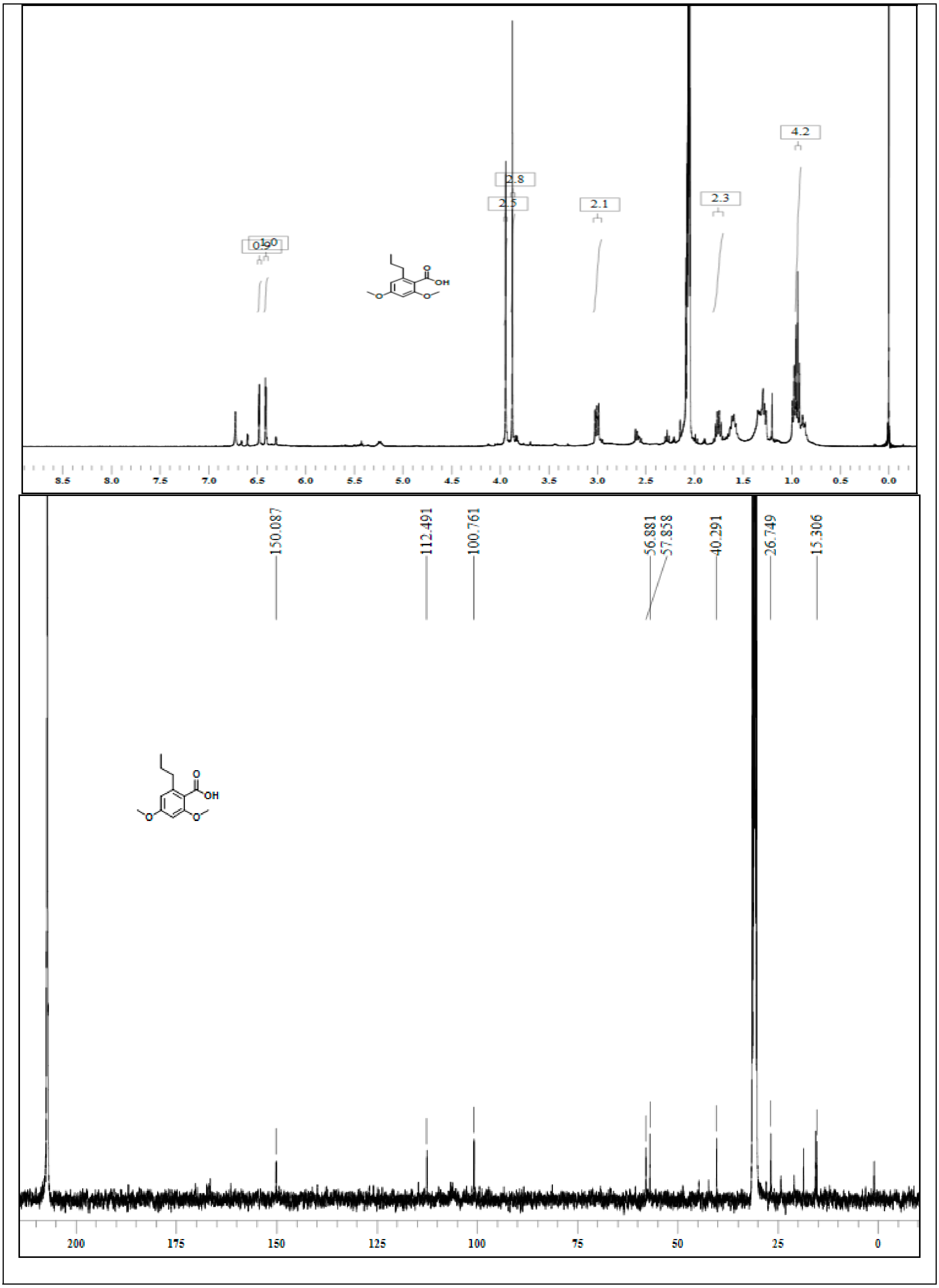

Figure S25: ^{1}H &^{13}C NMR Spectrum of 2, 4-di-O-Methyldivaic acid (**6**) (400 & 100 MHz, CD_3COCD_3).

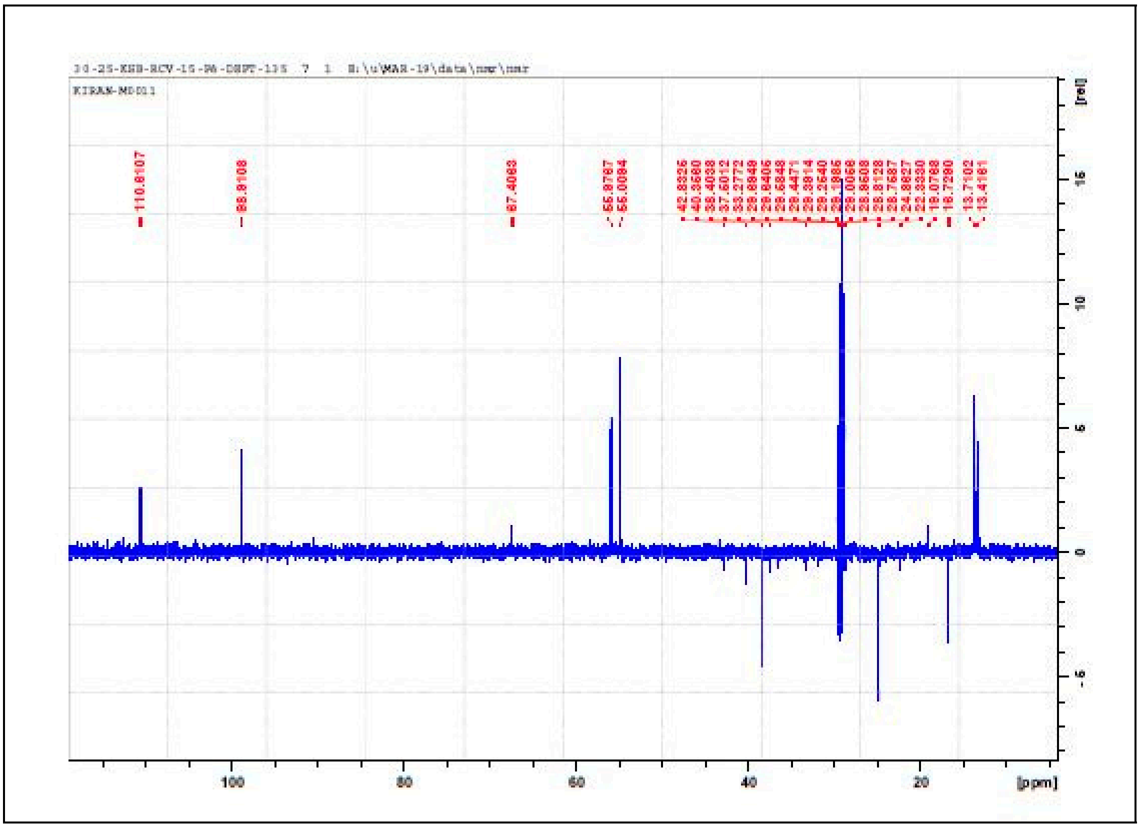

Figure S26: DEPT 135º NMR spectra of 2, 4-di-O-Methyldivaic acid (**6**).

NPL IICT
FTIR REPORT

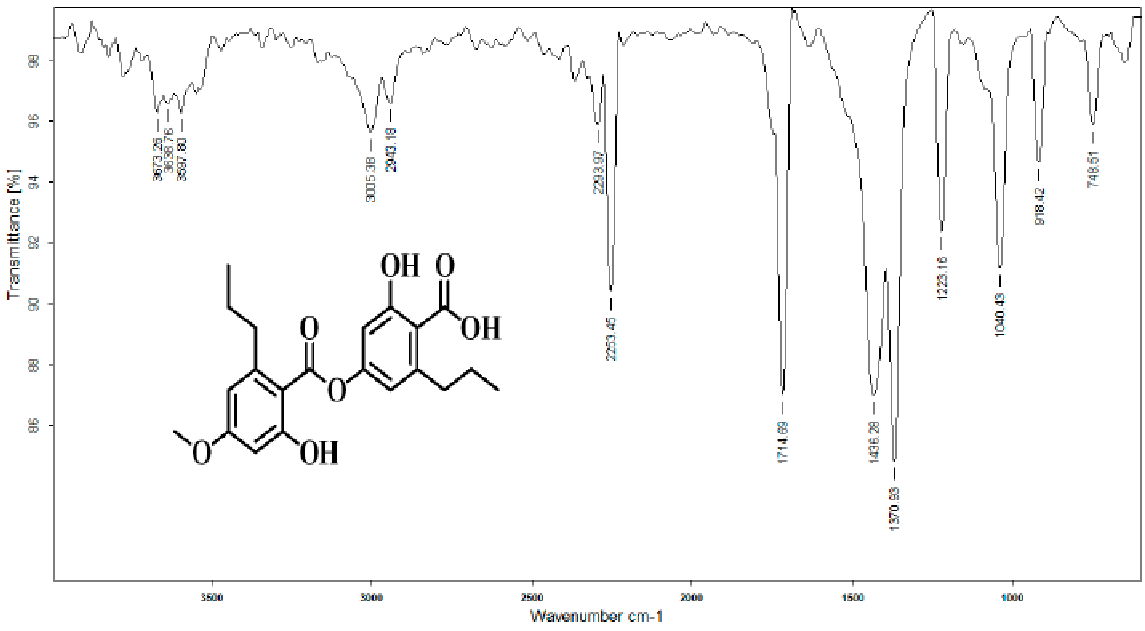

Figure S27: FTIR spectrum of Divaricatic acid (**7**).

Minimum:				-1.5				
Maximum:		5.0	10.0	50.0				
Mass	Calc. Mass	mDa	PPM	DBE	i-FIT	Norm	Conf(%)	Formula
387.1451	387.1444	0.7	1.8	10.5	644.4	n/a	n/a	C21 H23 O7

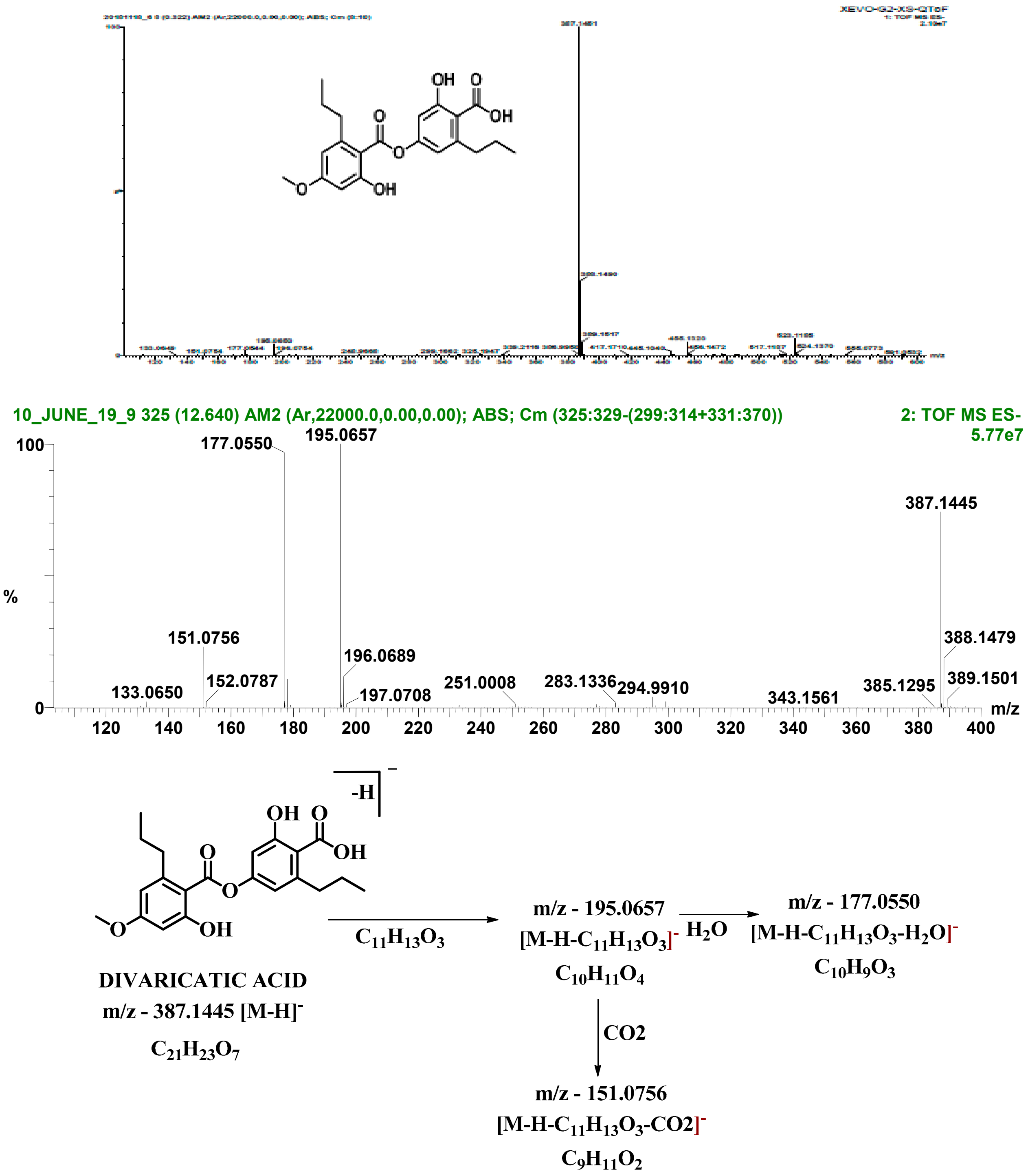

Figure S28: HRESIMS, MS/MS spectra and fragmentation pattern of Divaricatic acid (**7**).

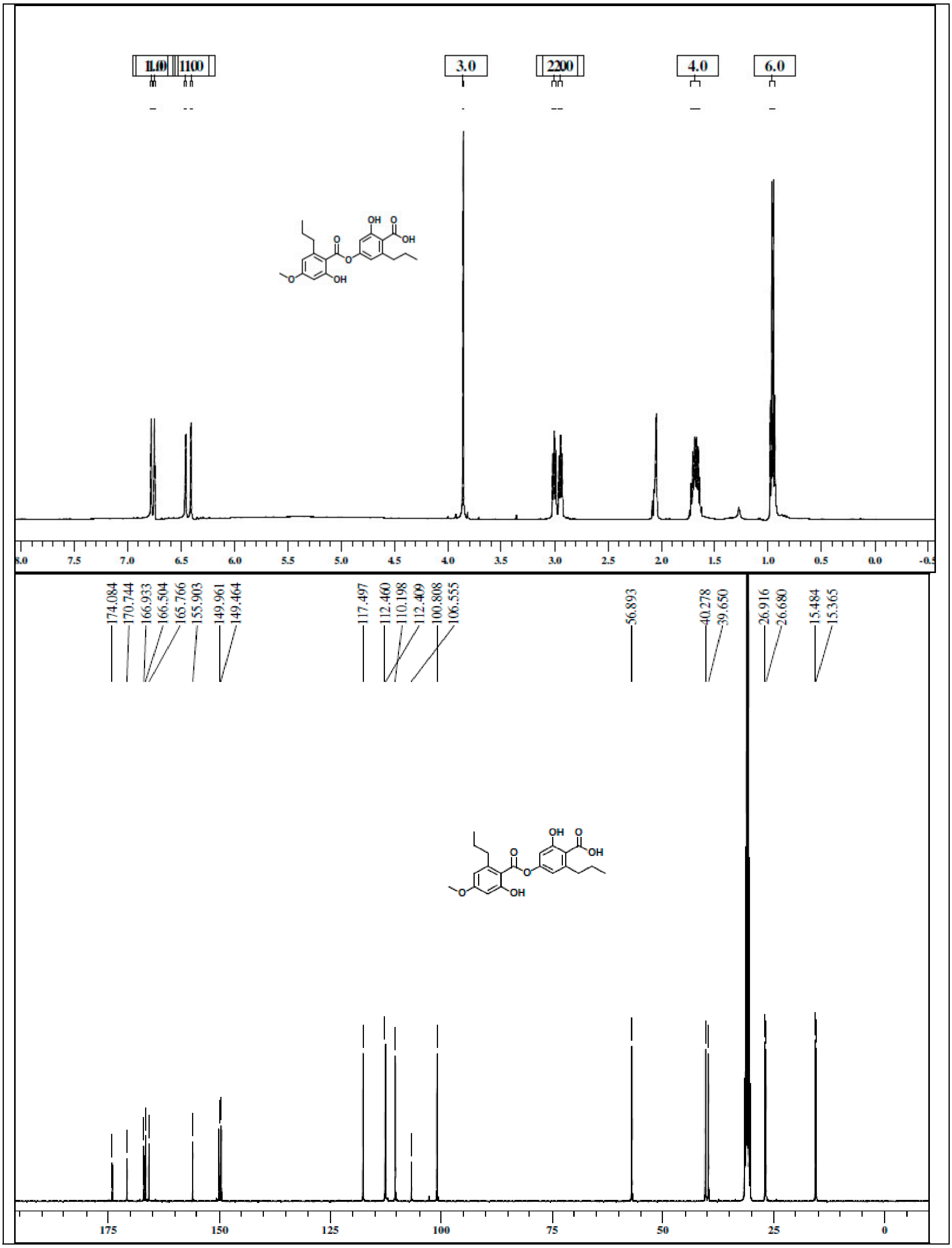

Figure S29: ^{1}H & ^{13}C NMR Spectrum of Divaricatic acid (**7**) (400 & 100 MHz, CD_3COCD_3).

Minimum:				-1.5				
Maximum:		5.0	10.0	50.0				
Mass	Calc. Mass	mDa	PPM	DBE	i-FIT	Norm	Conf(%)	Formula
343.1554	343.1545	0.9	2.6	9.5	942.9	n/a	n/a	C20 H23 O5

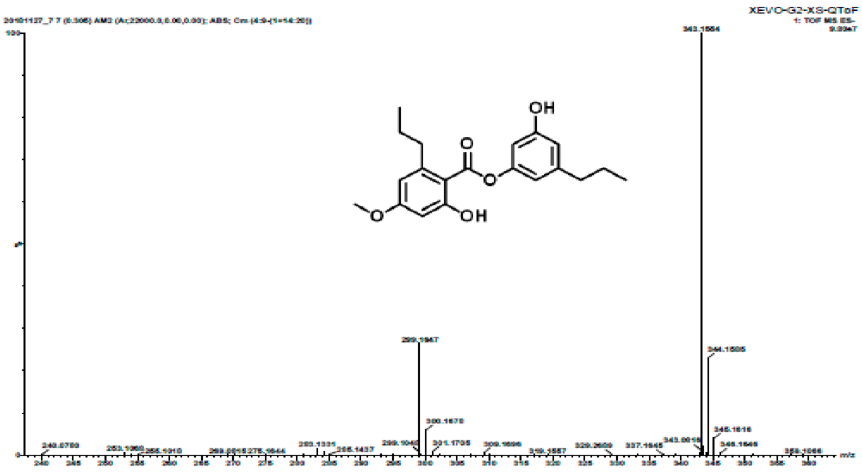

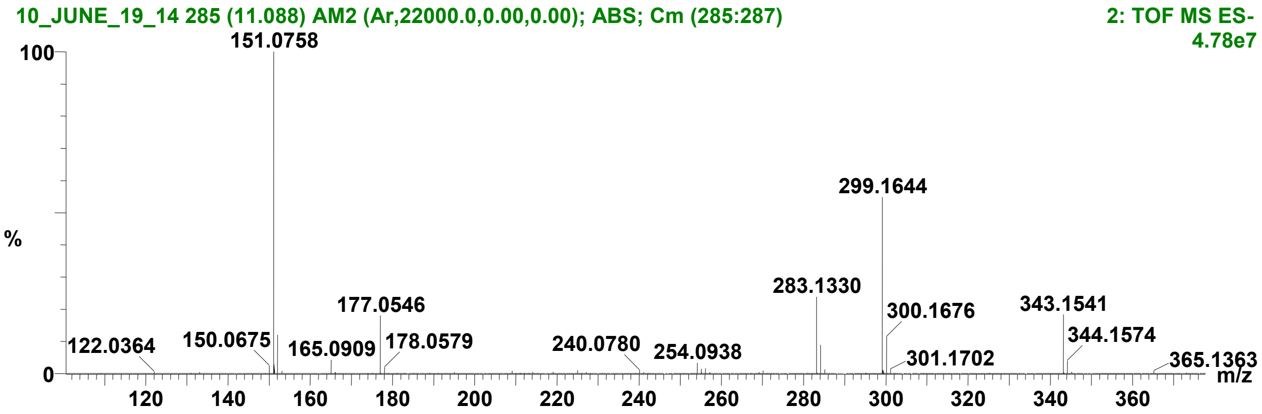

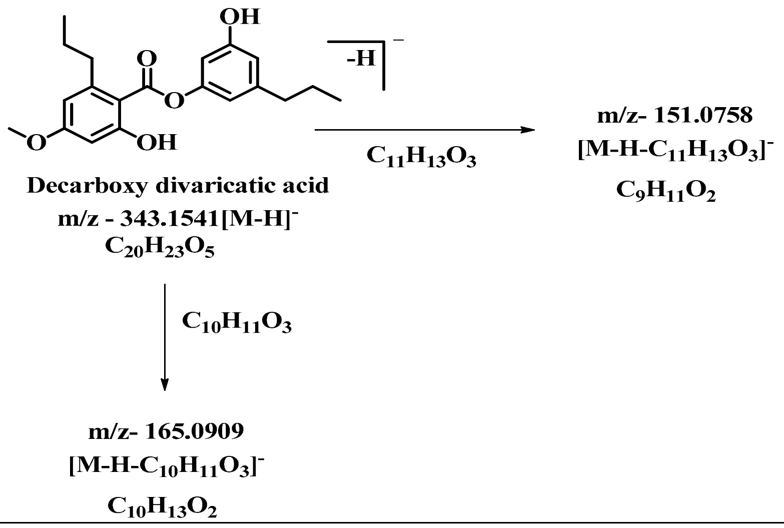

Figure S30: HRESIMS, MS/MS spectra and fragmentation pattern of Decarboxydivaricatic acid (**8**).

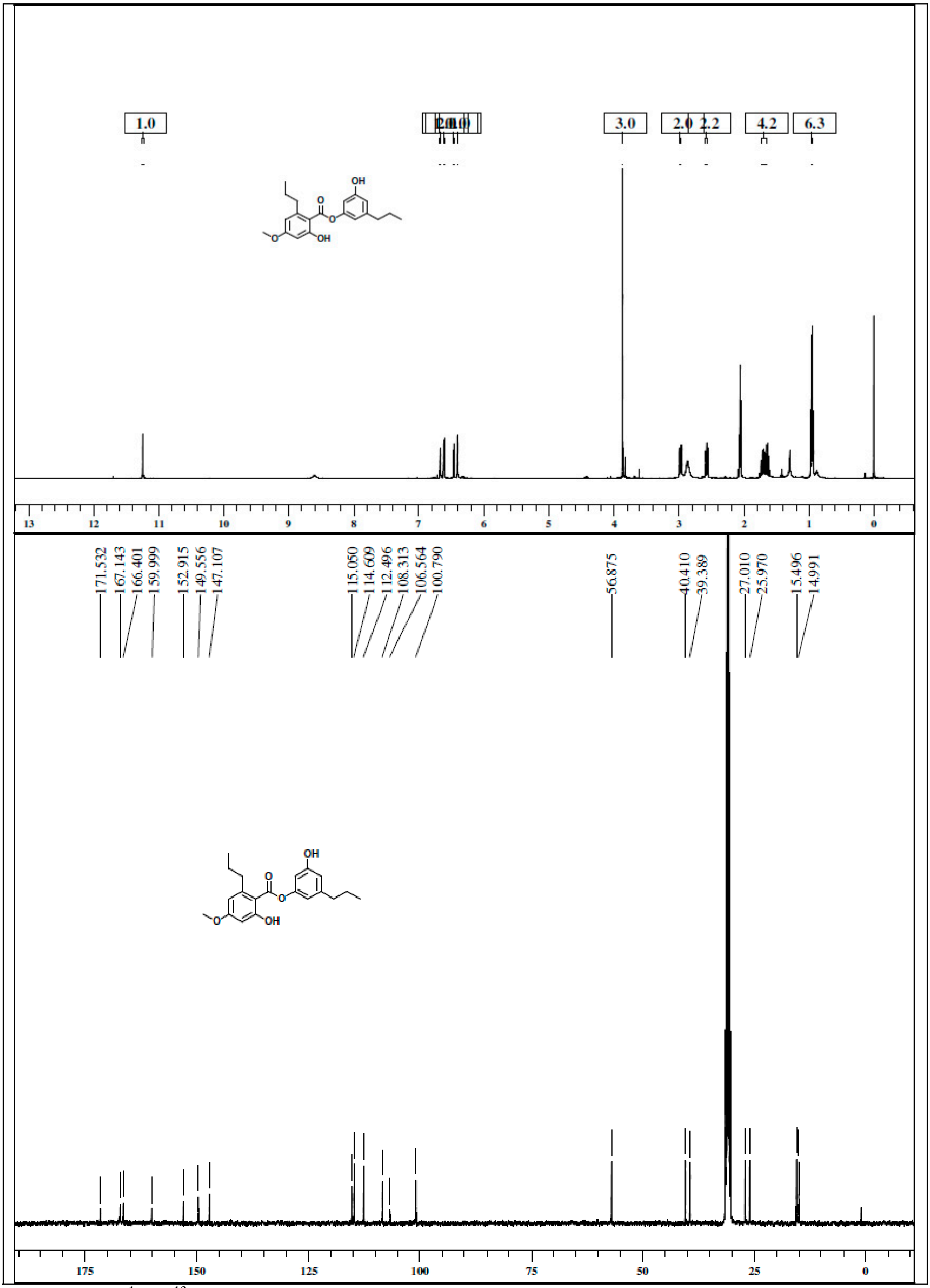

Figure S31: ^{1}H &^{13}C NMR Spectrum of Decarboxydivaricatic acid (**8**) (400 & 100 MHz, CD_3COCD_3).

Minimum:				-1.5				
Maximum:		5.0	5.0	50.0				
Mass	Calc. Mass	mDa	PPM	DBE	i-FIT	Norm	Conf(%)	Formula
371.1862	371.1858	0.4	1.1	9.5	764.4	n/a	n/a	C22 H27 O5

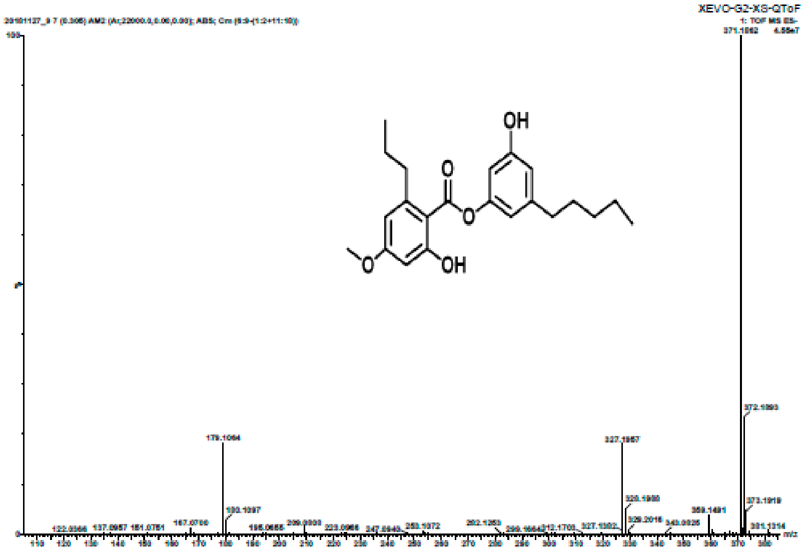

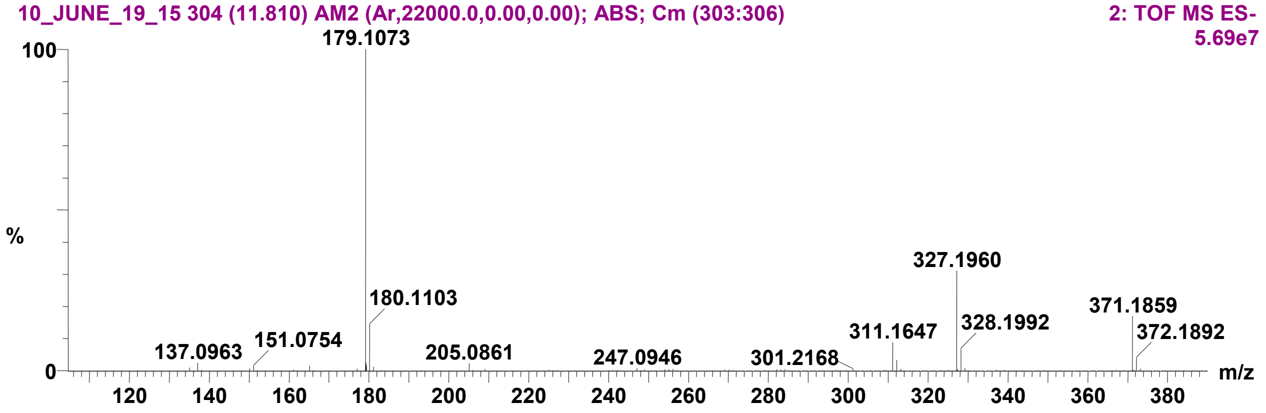

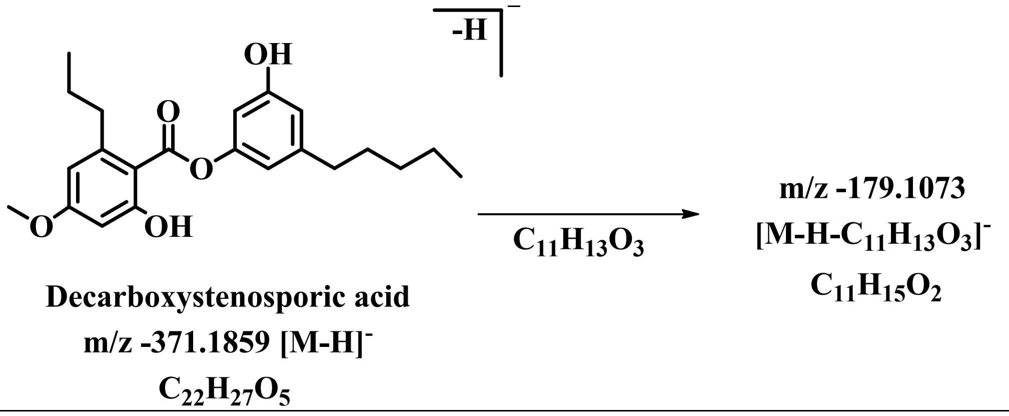

Figure S32: HRESIMS, MS/MS spectra and fragmentation pattern of Decarboxystenosporic acid (**9**).

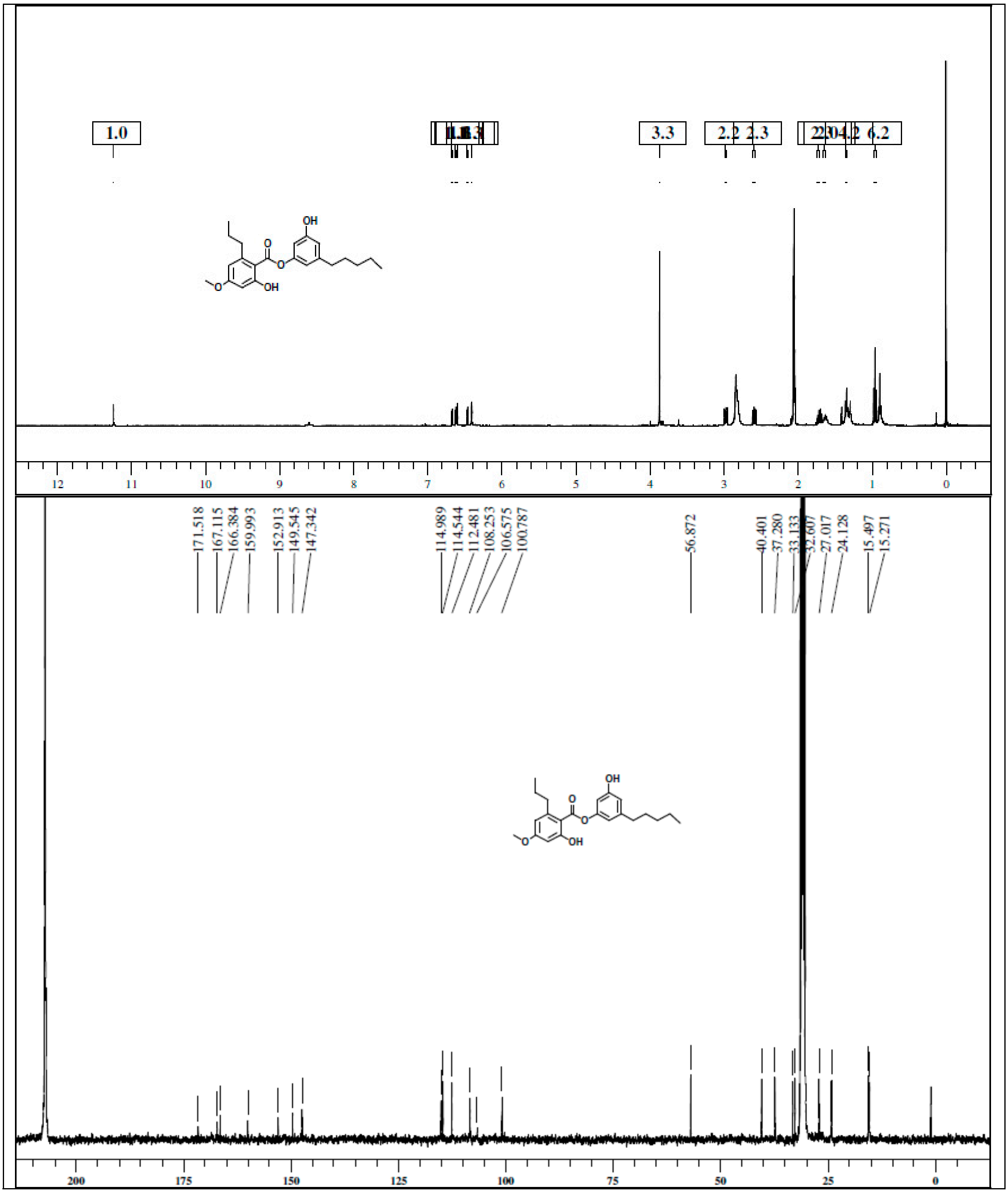

Figure S33: ^{1}H & ^{13}C NMR Spectrum of Decarboxystenosporic acid (**9**) (400 & 100 MHz, CD_3COCD_3).

Minimum:				-1.5				
Maximum:		5.0	10.0	50.0				
Mass	Calc. Mass	mDa	PPM	DBE	i-FIT	Norm	Conf(%)	Formula
223.0964	223.0970	-0.6	-2.7	5.5	533.5	n/a	n/a	C12 H15 O4

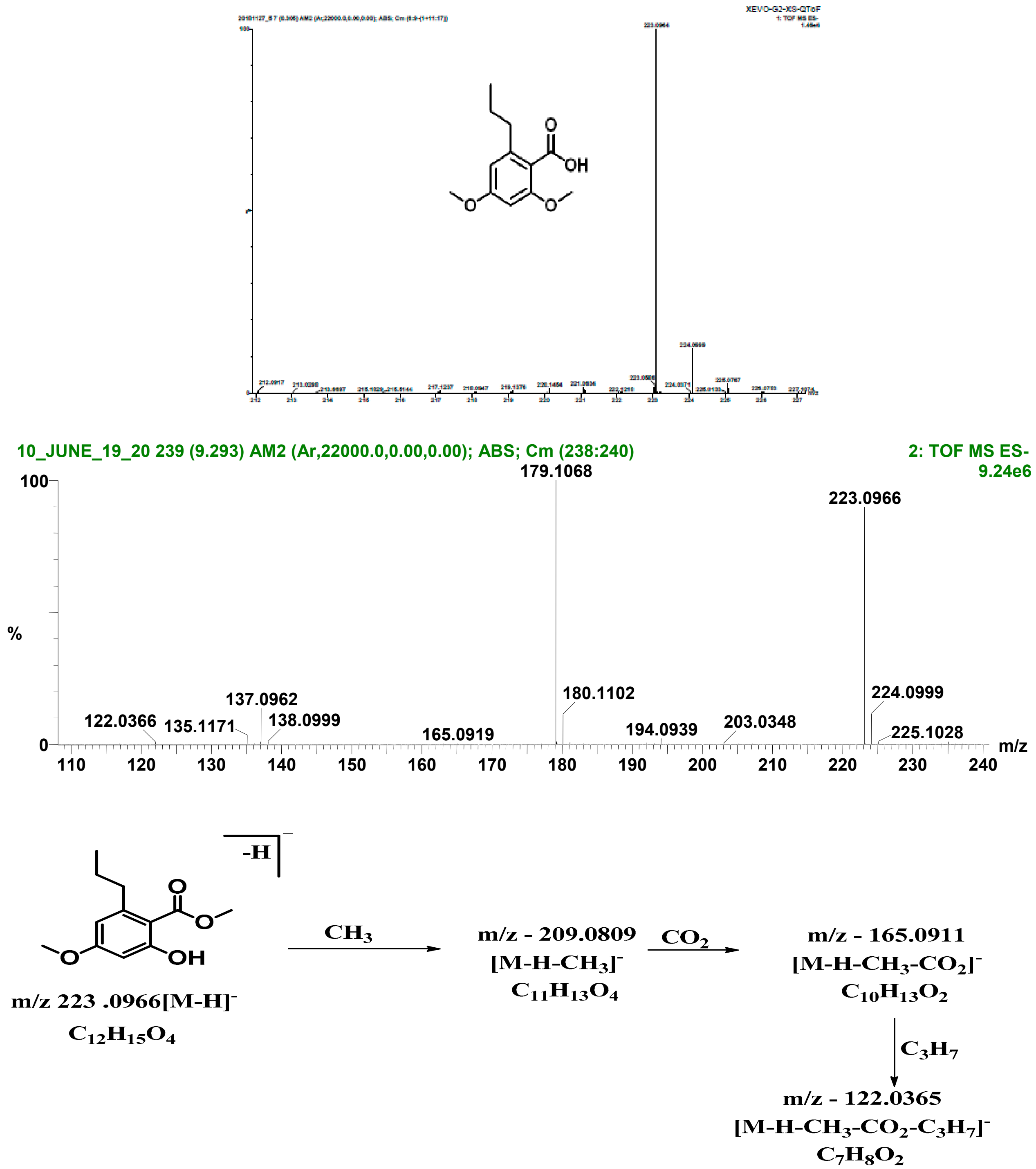

Figure S34: HRESIMS, MS/MS spectra and fragmentation pattern of Methyldivaricatinate (**10**).

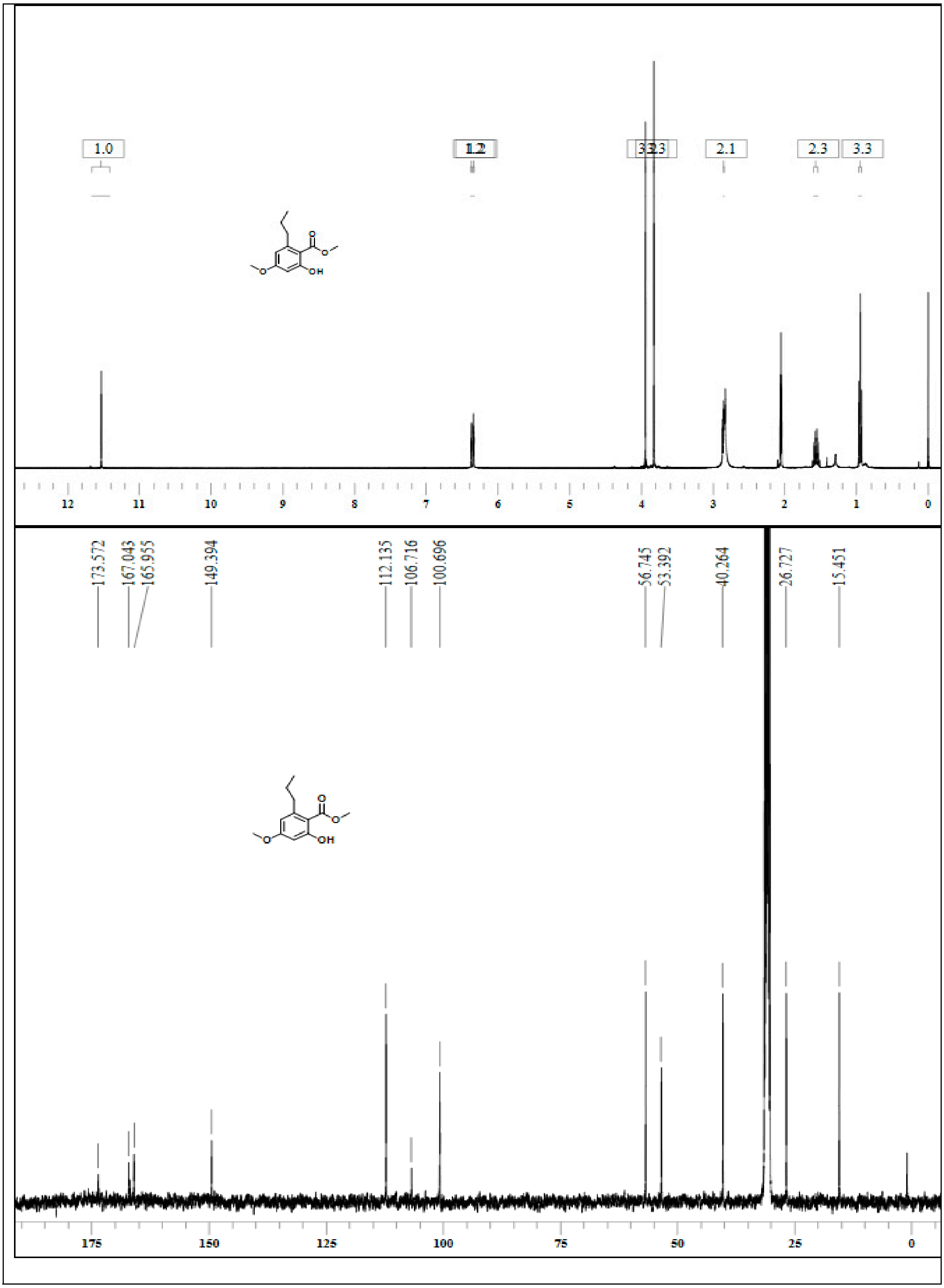

Figure S35: ^{1}H &^{13}C NMR Spectrum Methyldivaricatinate (**10**) (400 & 100 MHz, CD_3COCD_3).

	Calc. Mass	mDa	PPM	DBE	i-FIT	Norm	Conf(%)	Formula
Minimum:				-1.5				
Maximum:		5.0	10.0	50.0				
Mass	Calc. Mass	mDa	PPM	DBE	i-FIT	Norm	Conf(%)	Formula
209.0805	209.0814	-0.9	-4.3	5.5	690.4	n/a	n/a	C11 H13 O4

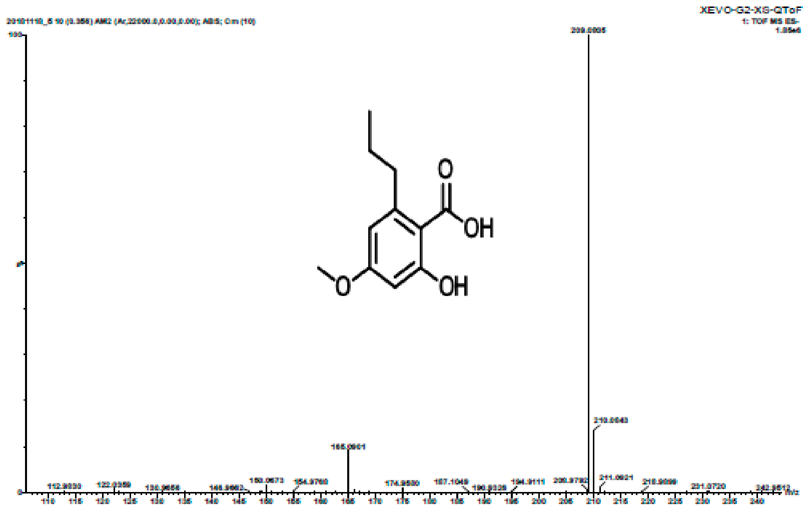

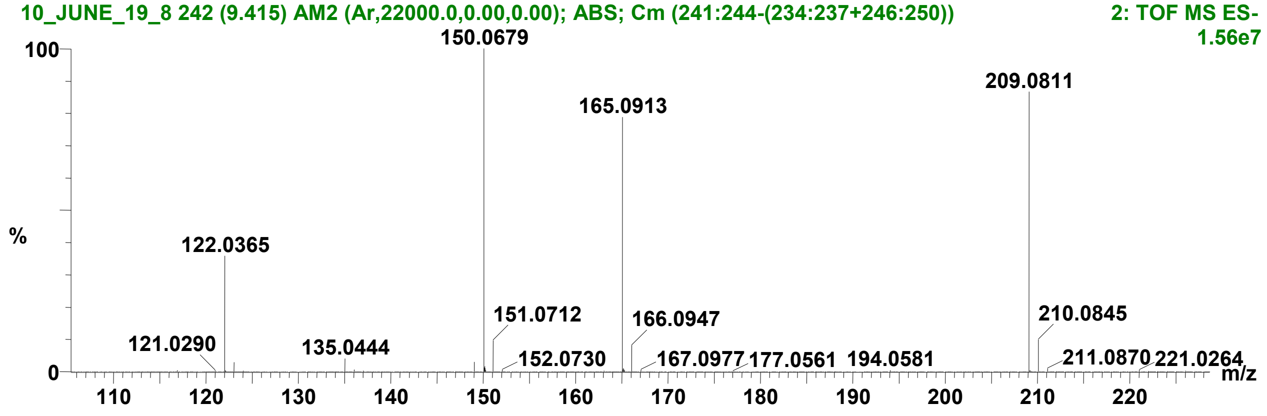

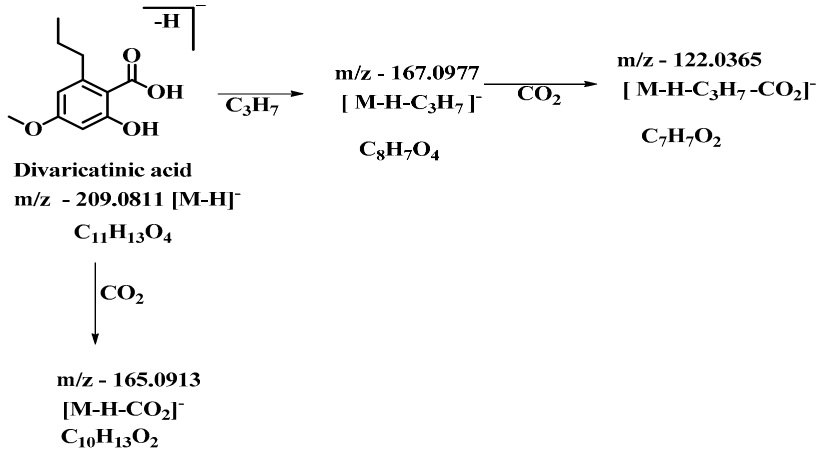

Figure S36: HRESIMS, MS/MS spectra and fragmentation pattern of Divaricatinic acid (**11**).

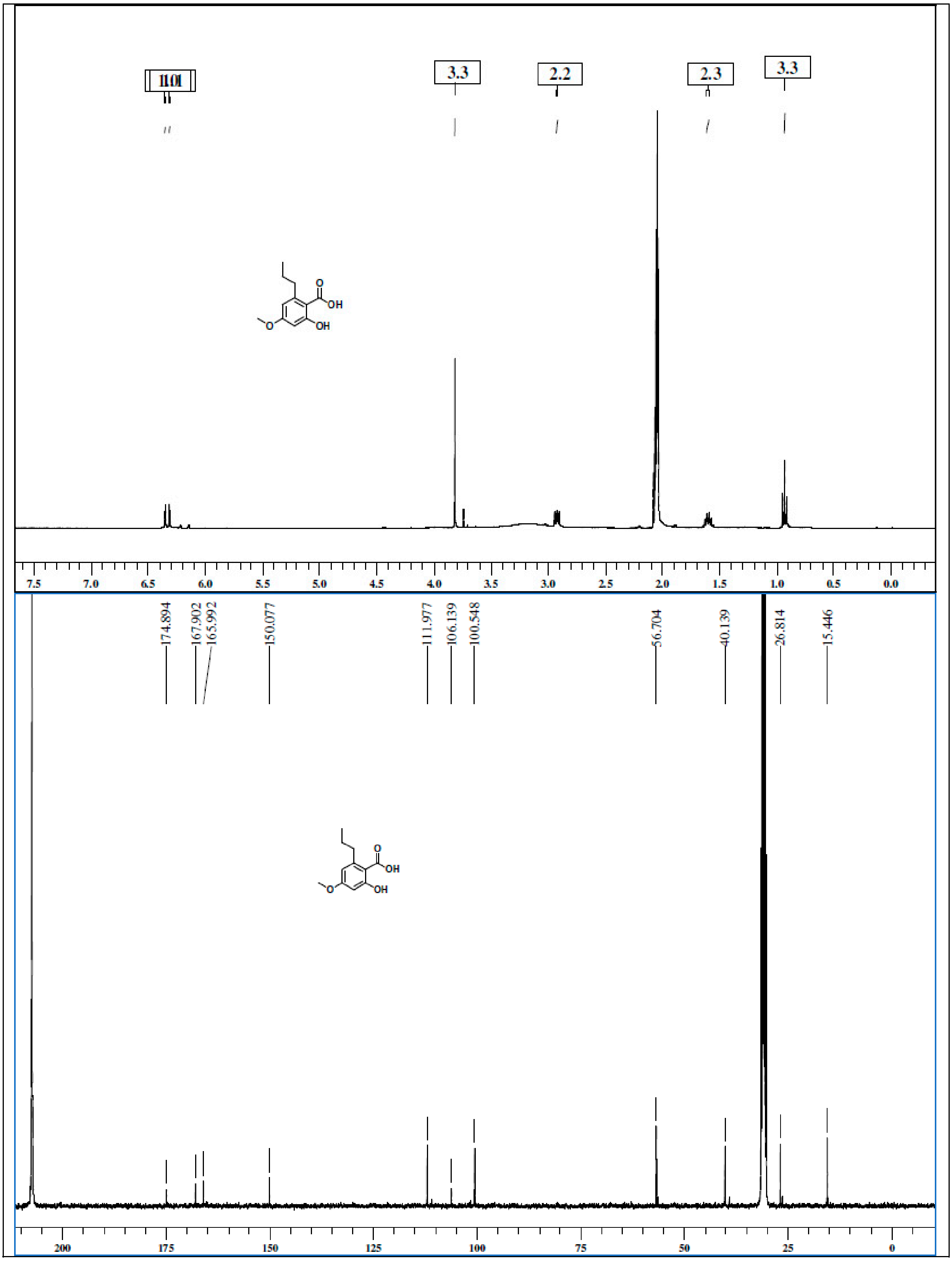

Figure S37: ^{1}H &^{13}C NMR Spectrum Divaricatinic acid (**11**) (400 & 100 MHz, CD_3COCD_3).

Minimum:				-1.5				
Maximum:		5.0	5.0	50.0				
Mass	Calc. Mass	mDa	PPM	DBE	i-FIT	Norm	Conf(%)	Formula
223.0961	223.0970	-0.9	-4.0	5.5	623.5	n/a	n/a	C12 H15 O4

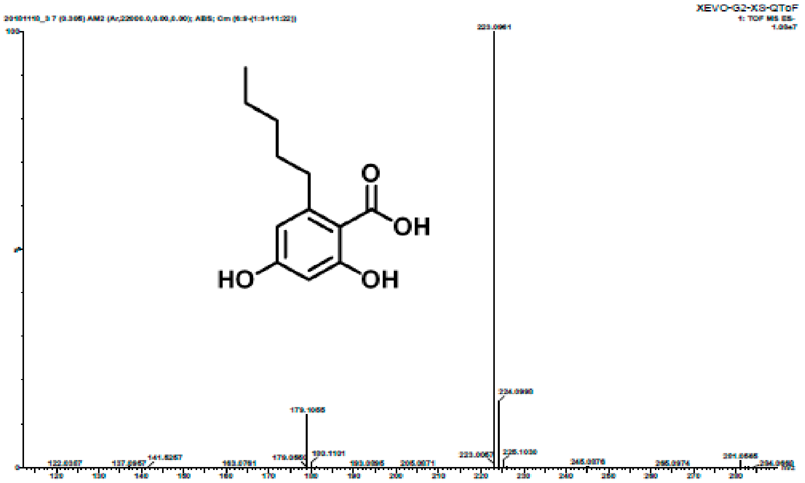

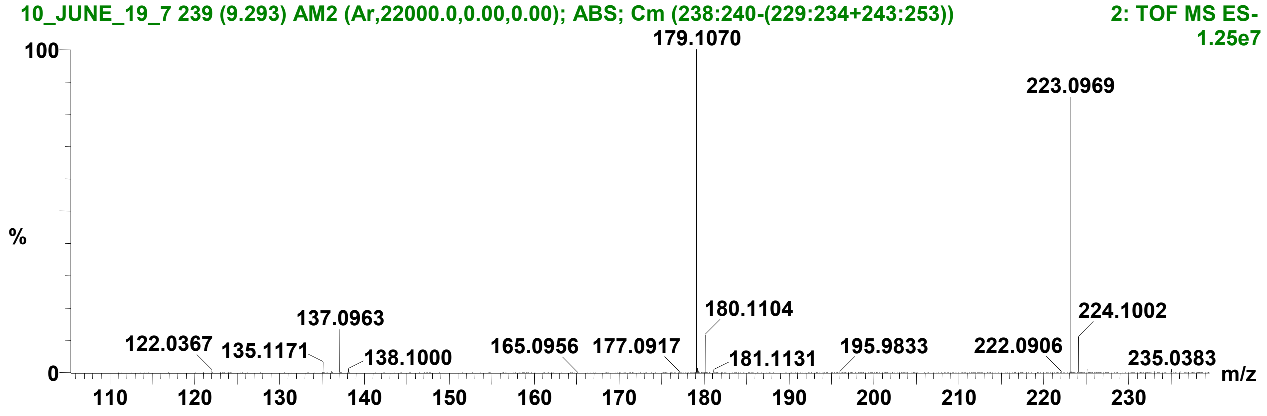

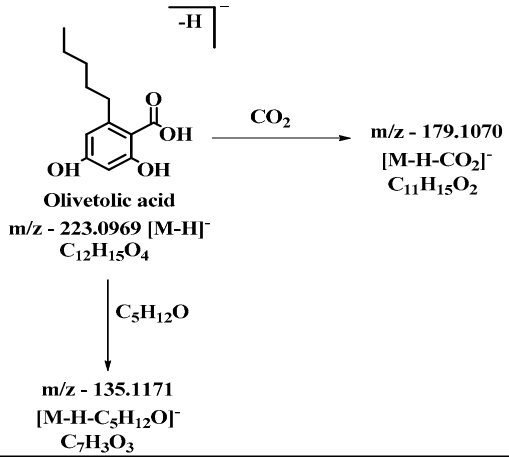

Figure S38: HRESIMS, MS/MS spectra and fragmentation pattern of Olivetolic acid (**12**).

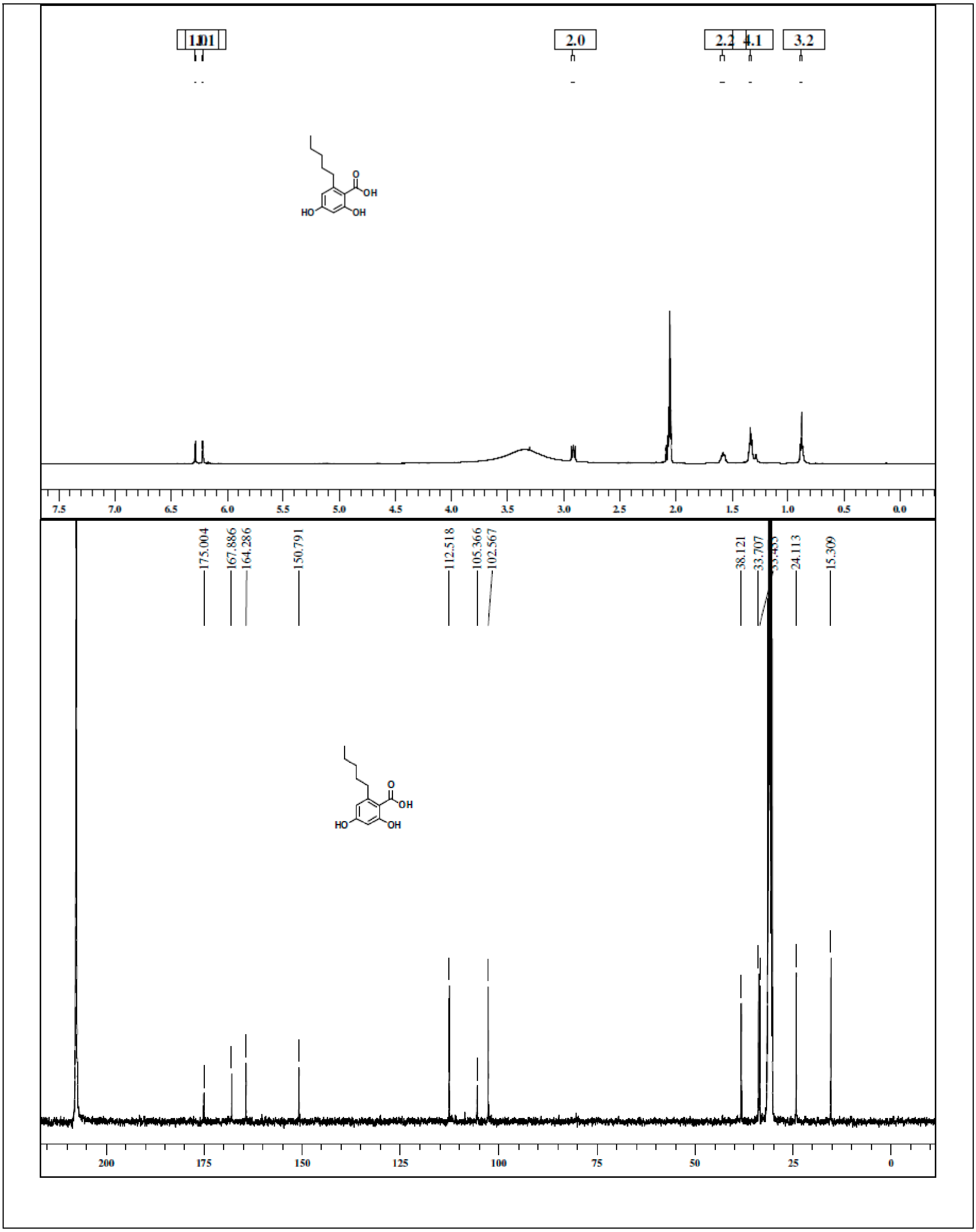

Figure S39: ^{1}H &^{13}C NMR Spectrum of Olivetolic acid (**12**) (400 & 100 MHz, CD_3COCD_3).

Minimum:				-1.5				
Maximum:		5.0	5.0	50.0				
Mass	Calc. Mass	mDa	PPM	DBE	i-FIT	Norm	Conf(%)	Formula
165.0916	165.0916	0.0	0.0	4.5	512.3	n/a	n/a	C10 H13 O2

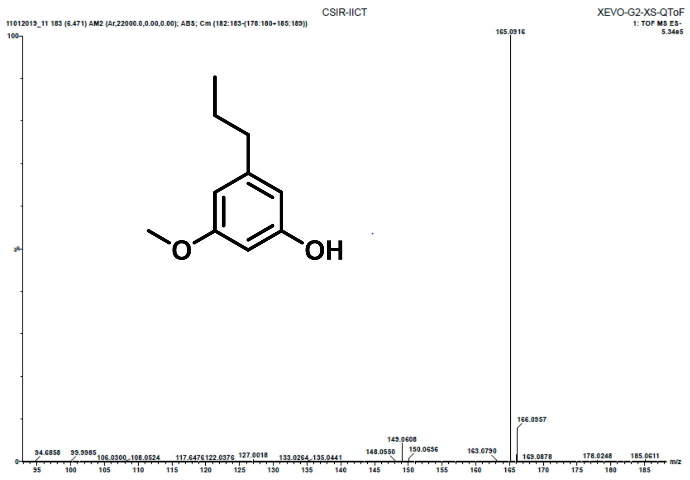

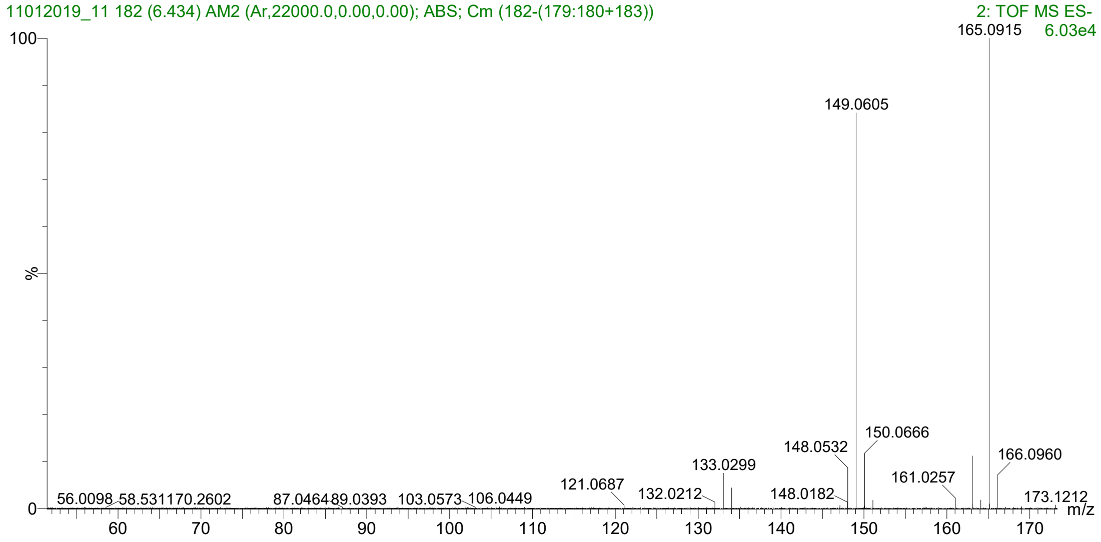

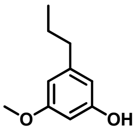

Divarinolmonomethylether
m/z - 165.0916 [M-H]$^-$
$C_{10}H_{13}O_2$

Figure S40: HRESIMS, MS/MS Spectra and fragmentation pattern of Divarinolmonomethylether (**13**).

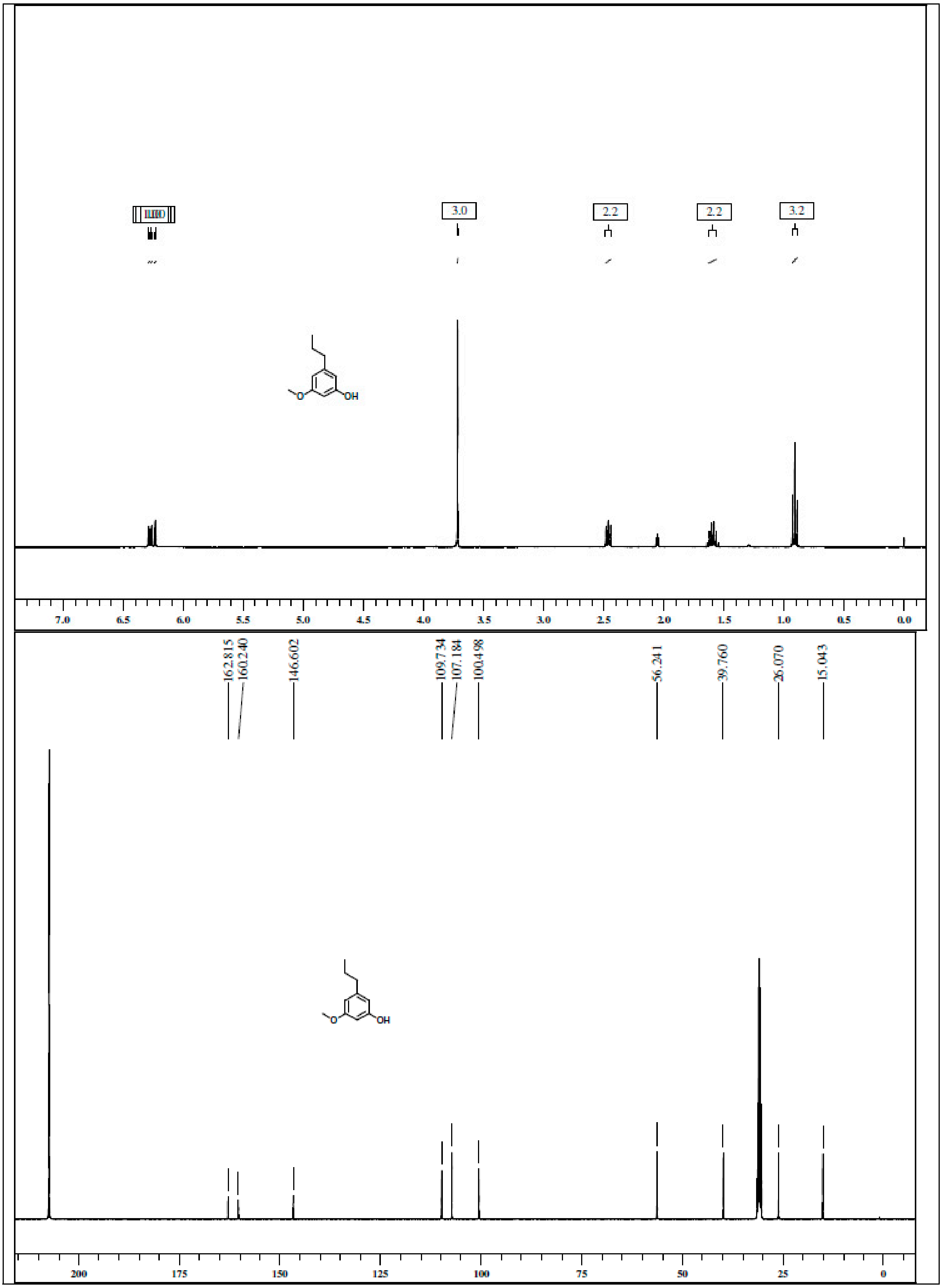

Figure S41: ^{1}H &^{13}C NMR Spectrum of Divarinolmonomethylether (**13**) (400 & 100 MHz, CD_3COCD_3).

Minimum:				-1.5				
Maximum:		5.0	5.0	50.0				
Mass	Calc. Mass	mDa	PPM	DBE	i-FIT	Norm	Conf(%)	Formula
151.0399	151.0395	0.4	2.6	5.5	924.3	n/a	n/a	C8 H7 O3

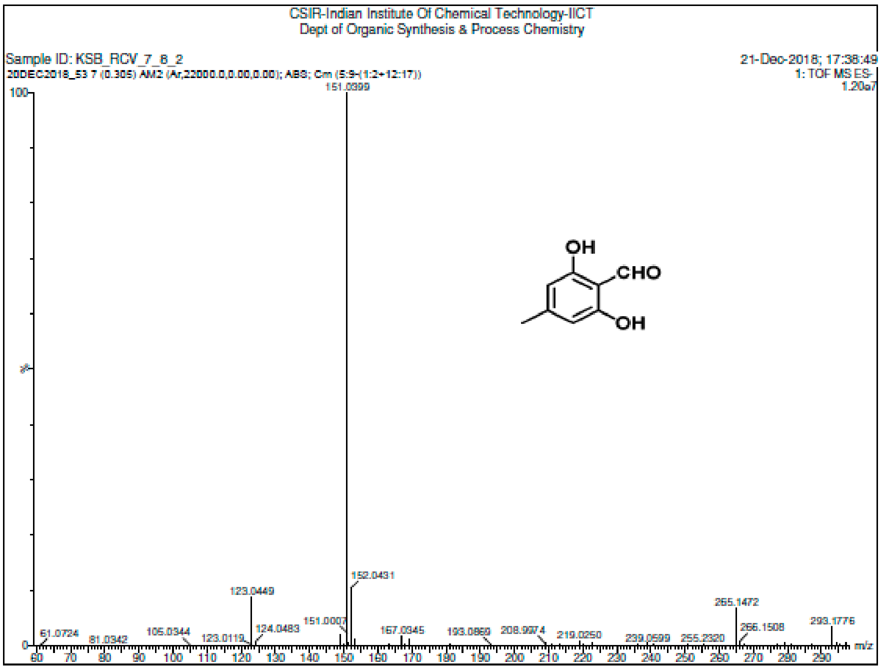

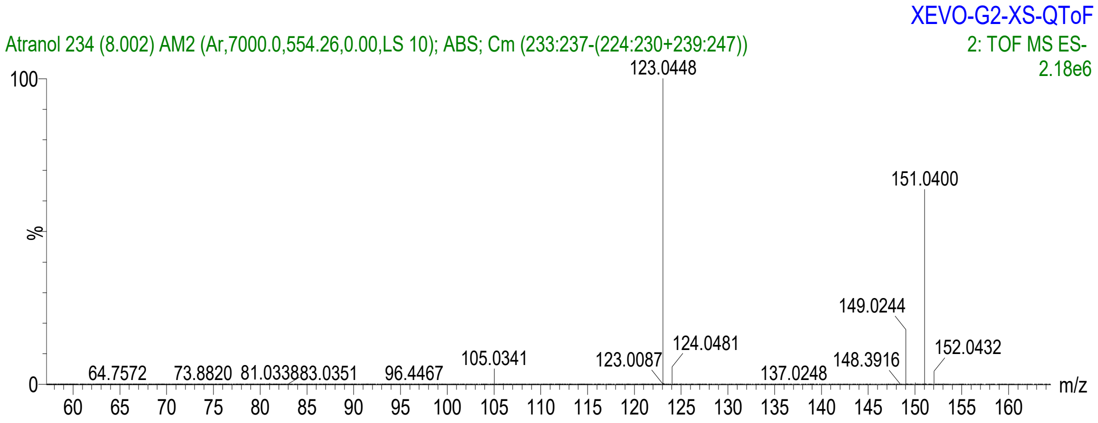

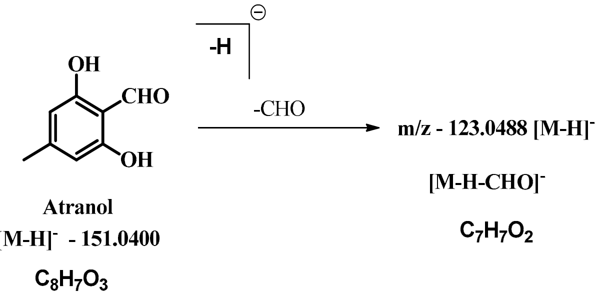

Figure S42: HRESIMS, MS/MS Spectra and fragmentation pattern of Atranol (**14**).

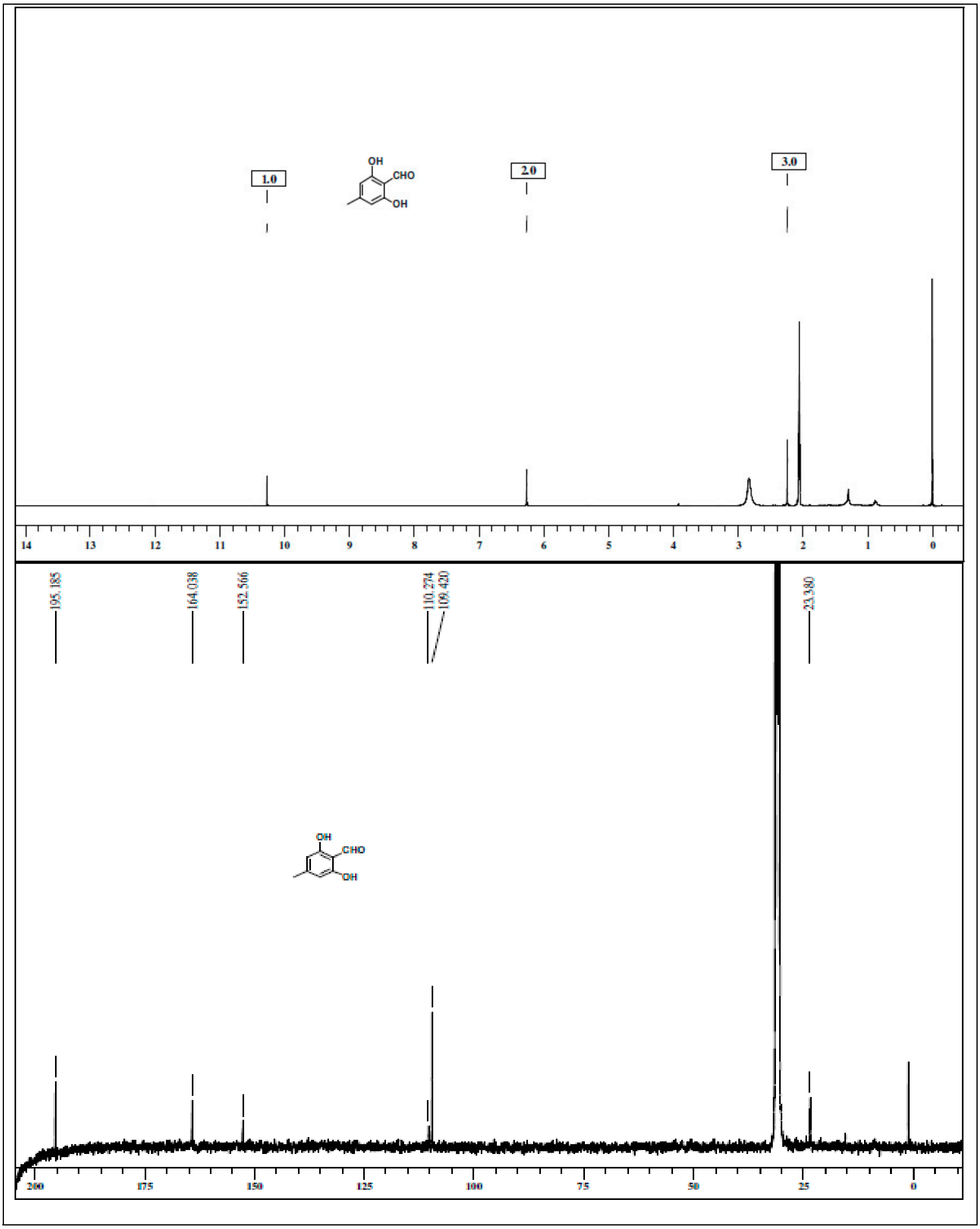

Figure S43: ^{1}H &^{13}C NMR Spectrum of Atranol (**14**) (400 & 100 MHz, CD_3COCD_3).

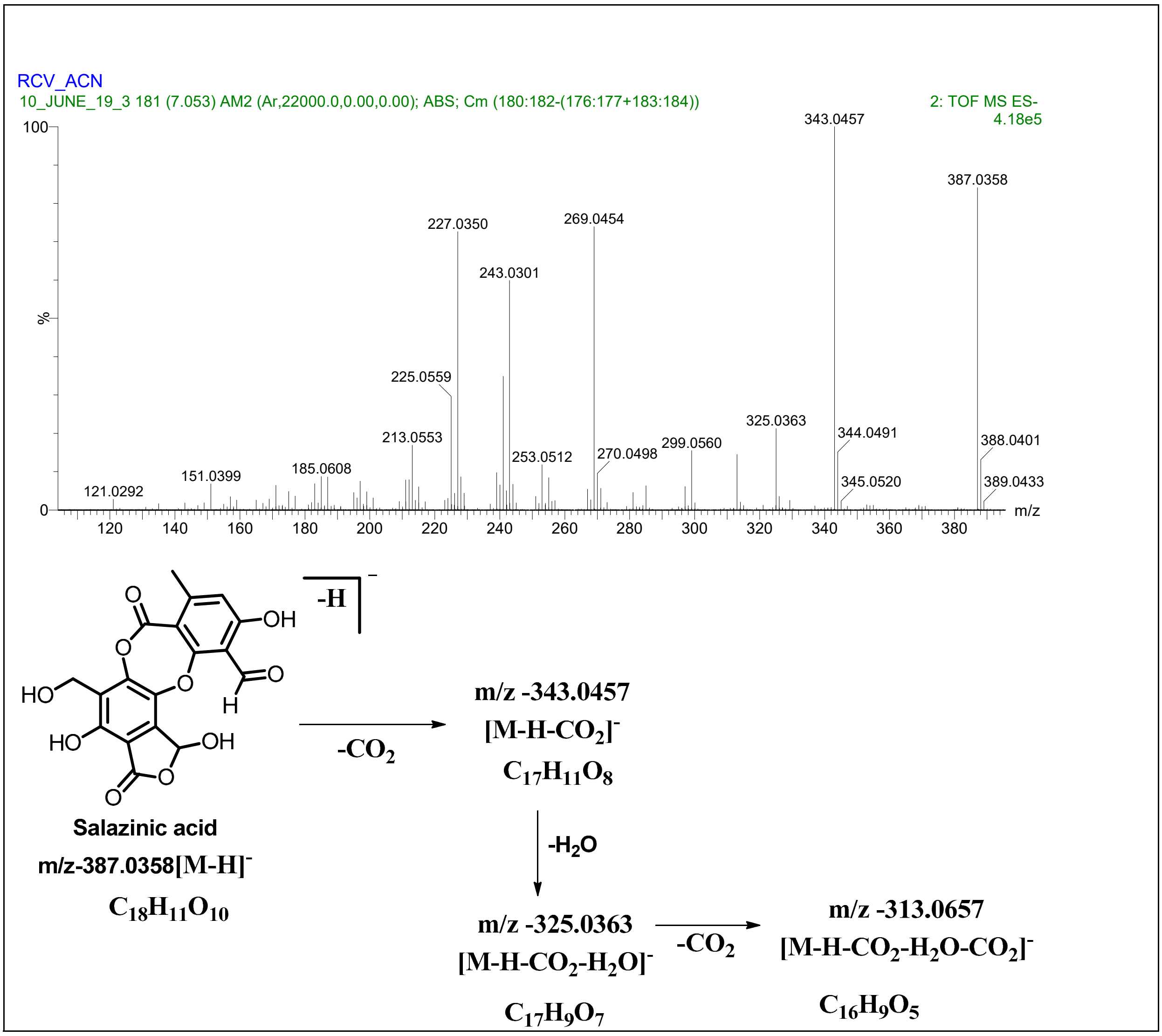

Figure S44: MS/MS Spectra and fragmentation pattern of Salazinic acid.

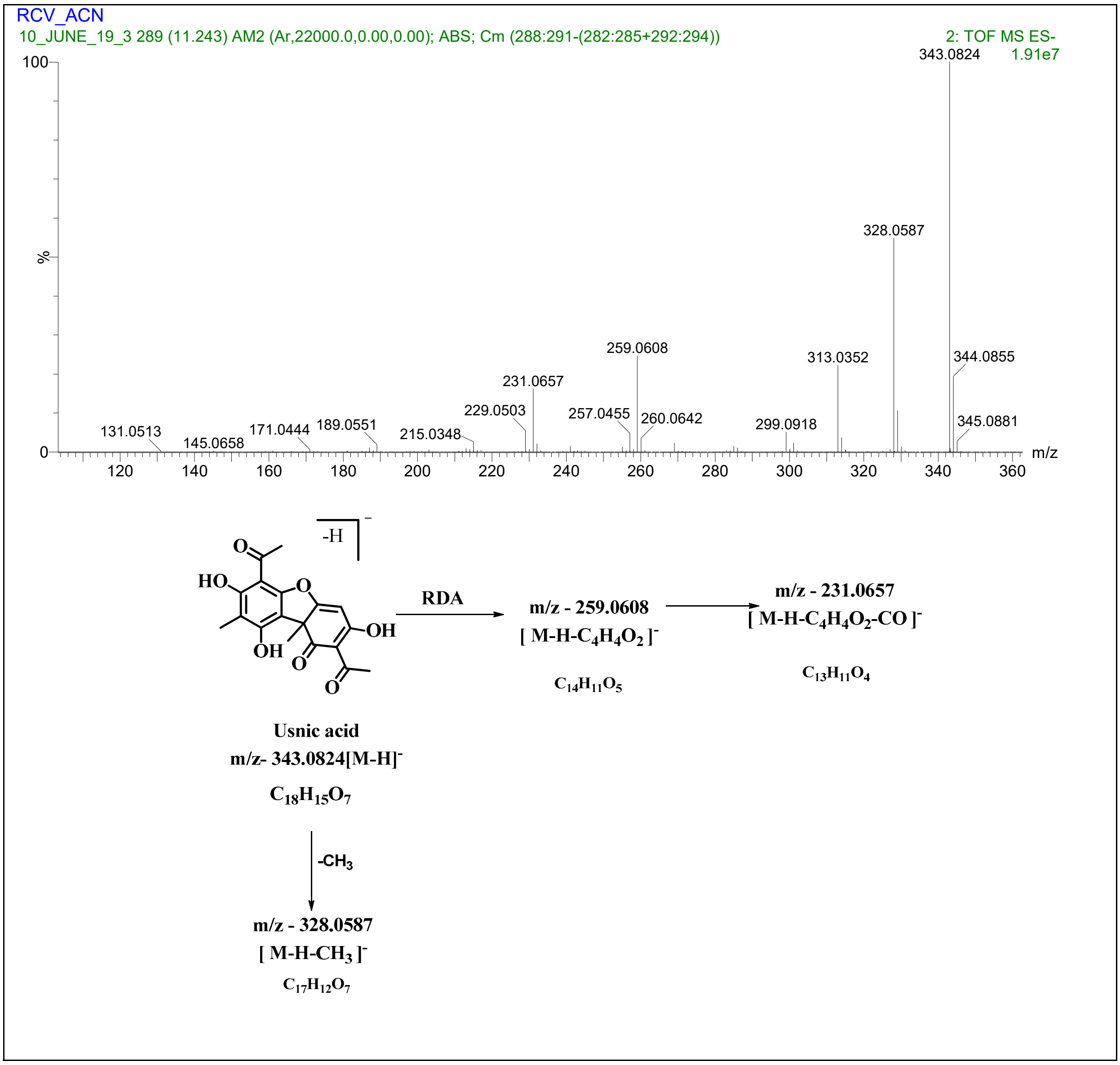

Figure S45: MS/MS Spectra and fragmentation pattern of Usnic acid.

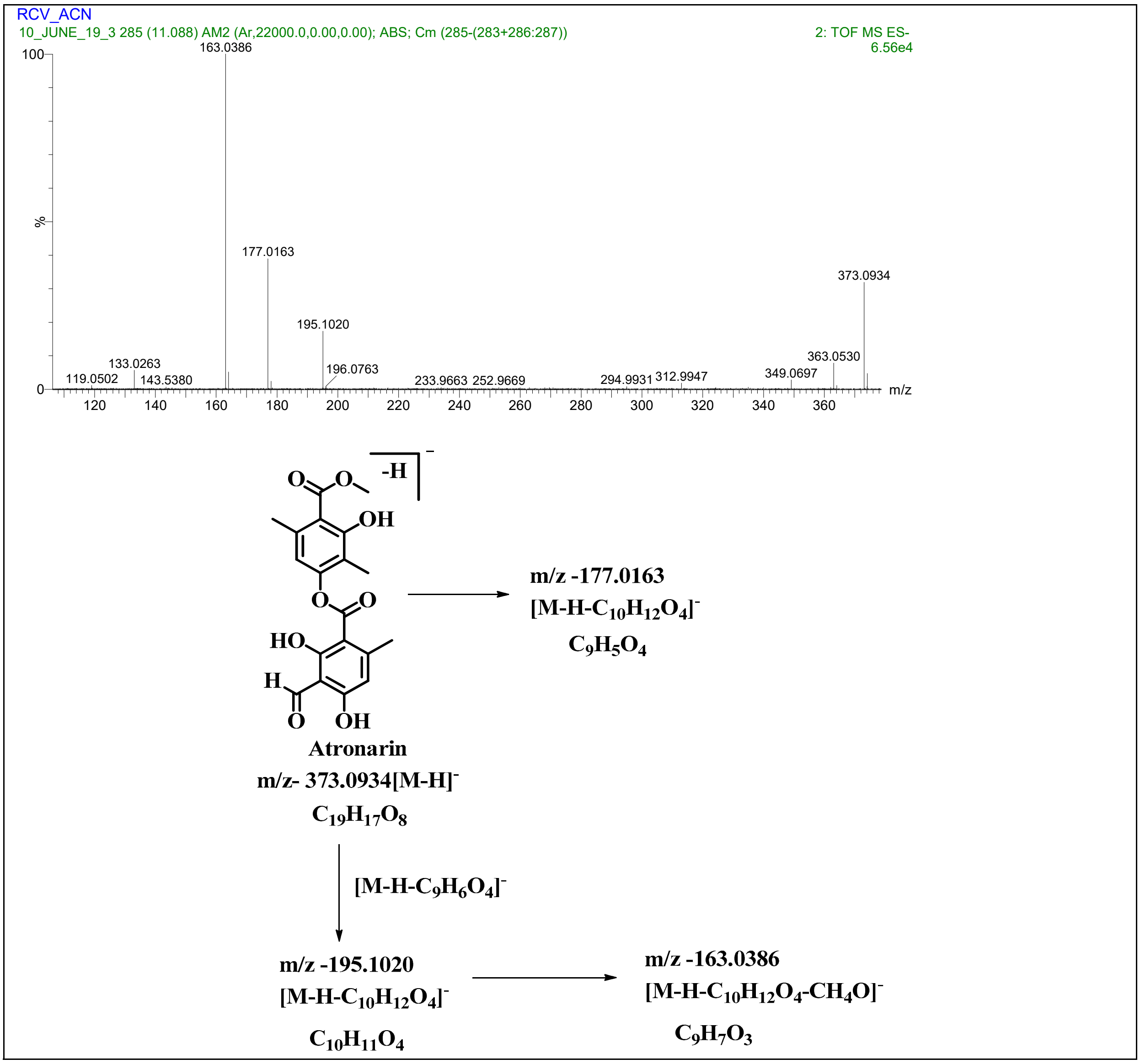

Figure S46: MS/MS Spectra and fragmentation pattern of Atronarin.

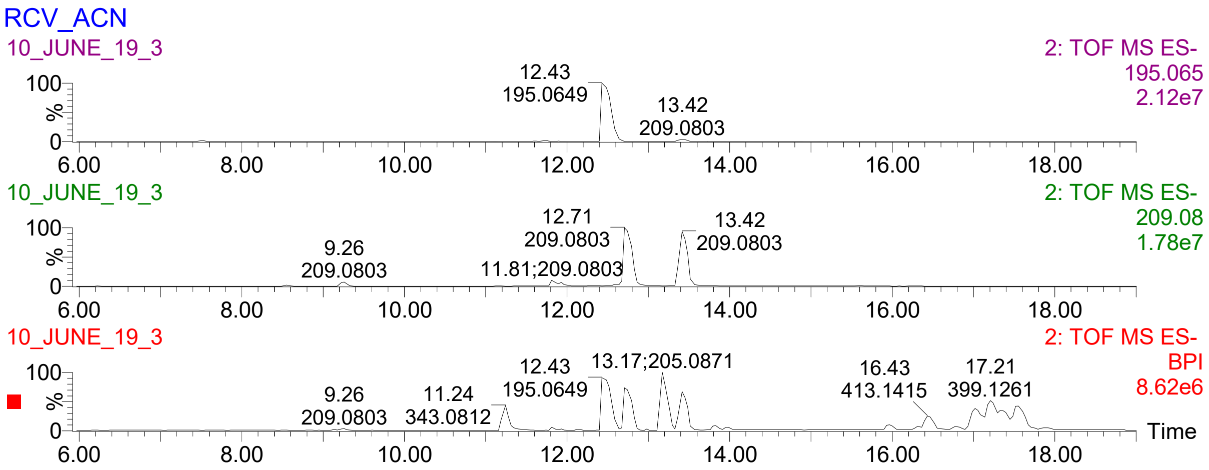

Figure S47: TIC of *R.conduplicans:* a) 209 ion extracted chromatogram*:* b) 195 ion extracted chromatogram.

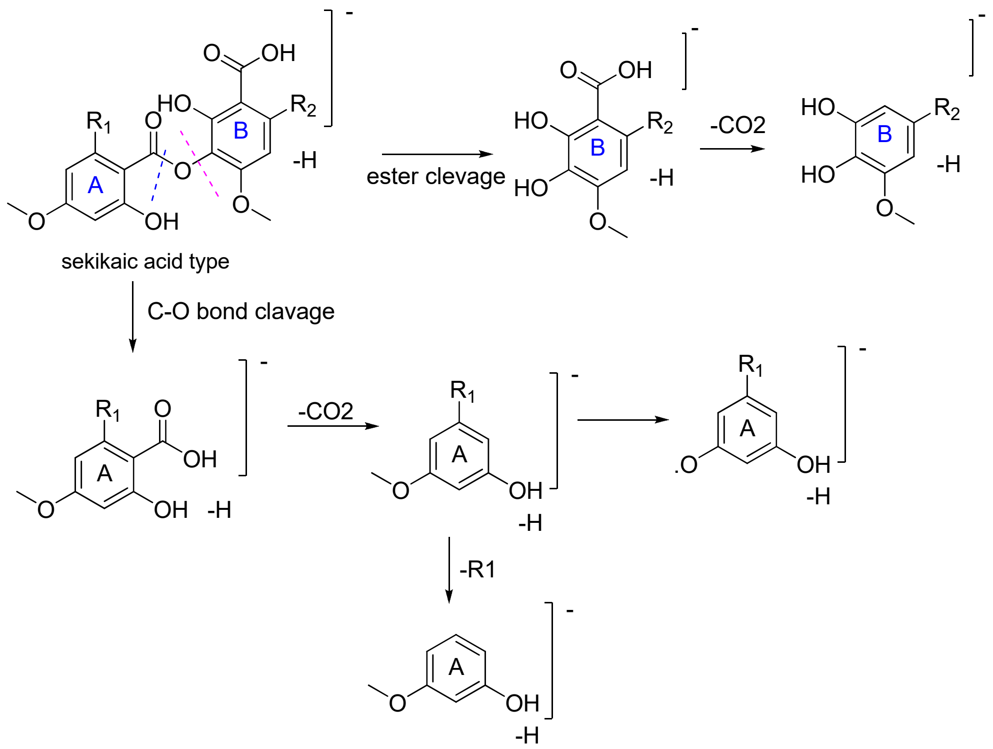

Figure S48: Common fragmentation of depsides, based on ESI (-) MS/MS of Sekikaic acid (**1**).

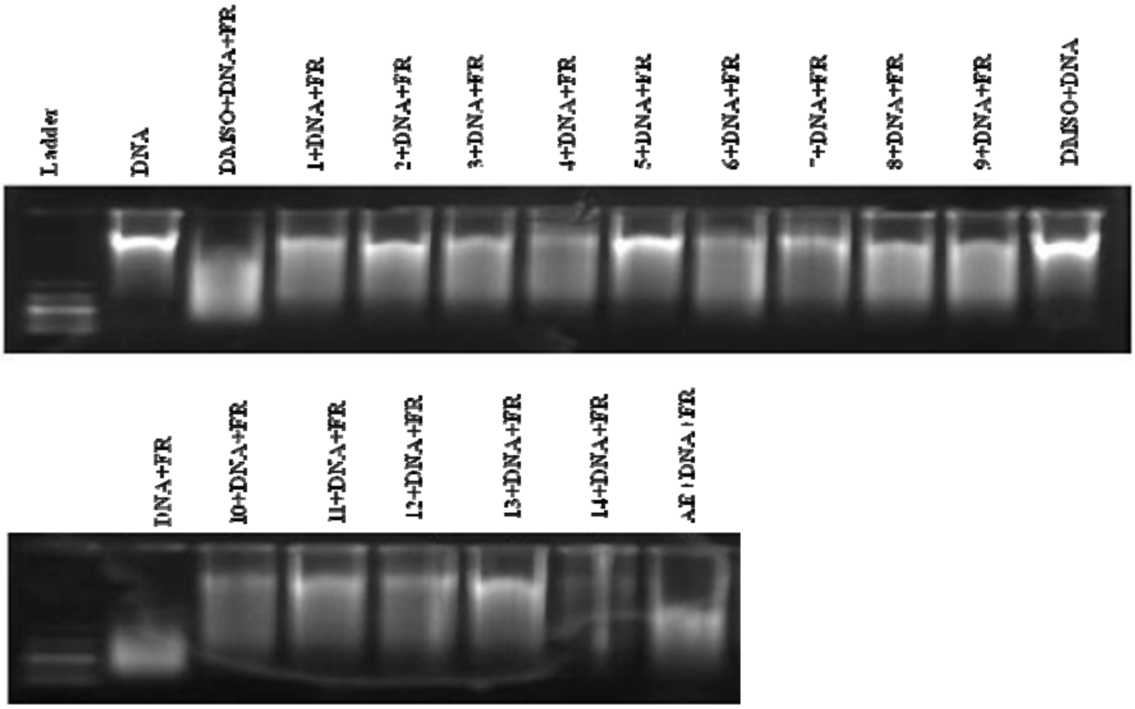

Figure S49. *In vitro* DNA damage assay. Compounds (**1-14**) and *R. conduplicans.* AE were incubated with DNA and Fenton Reagent and DNA damage was recorded with Agarose Gel electrophoresis.
